# KIF18A induces the EMT process of hepatoma cells through the 5-LOX-dependent arachidonic acid pathway

**DOI:** 10.1371/journal.pone.0333385

**Published:** 2025-10-13

**Authors:** Yibo Wang, Tao Cai, Yajie Chen, Wenpeng Liu, Baowang Liu, Xin Zhao, Yang Wang, Jinglin Cao, Qiang Zeng

**Affiliations:** Department of Hepatobiliary Surgery, Hebei Medical University Third Hospital, Shijiazhuang, China; Longgang Otorhinolaryngology Hospital & Shenzhen Key Laboratory of Otorhinolaryngology, Shenzhen Institute of Otorhinolaryngology, CHINA

## Abstract

The high morbidity and mortality rates of liver cancer prompt us to constantly explore new therapeutic strategies. The arachidonic acid metabolism-related pathways play a crucial regulatory role in the growth, invasion and angiogenesis of liver cancer cells, thus becoming a key entry point in liver cancer research. This study aims to explore the role of KIF18A in the epithelial-mesenchymal transition (EMT) of liver cancer cells through the 5-LOX-dependent arachidonic acid pathway, and to construct a prognostic model to predict the prognostic risk of patients with hepatocellular carcinoma (HCC). Through bioinformatics techniques, we screened out prognosis genes related to arachidonic acid metabolism in HCC and constructed a prognostic model of HCC. We evaluated and verified the model, and analyzed the functional enrichment analysis and PPI network analysis of key arachidonic acid metabolism-related genes, and screened out the key prognostic gene KIF18A. Experimental results showed that the expression level of KIF18A in liver cancer cells was significantly higher than that in normal liver cells, and the high expression of KIF18A was associated with the poor prognosis of patients with liver cancer. The overexpression of KIF18A could significantly promote the proliferation, invasion and migration abilities of liver cancer cells, while the knockdown of KIF18A inhibited these cellular behaviors. In addition, KIF18A could promote EMT of liver cancer cells, downregulate E-cadherin and upregulate the expression of N-cadherin. We also found that KIF18A could induce the upregulation of 5-LOX expression and increase the levels of arachidonic acid metabolites, while the 5-LOX inhibitor U73122 could reverse this effect. Therefore, KIF18A may promote the proliferation, invasion, and EMT of liver cancer cells by activating the 5-LOX-dependent arachidonic acid pathway. This study provides a new strategy for the prognostic assessment and targeted therapy of HCC and reveals the important role of KIF18A in the development of liver cancer.

## Introduction

Primary liver cancer (PLC), one of the common malignant tumors, poses a serious risk to human health. According to the latest global cancer burden data of the World Health Organization, there were over 1 million new cases of PLC and about 900,000 deaths in 2022, making it one of most lethal cancers worldwide. In the past five years, China has witnessed an average of approximately 450,000 new liver cancer cases each year, accounting for over 40% of the incidence. The overall survival (OS) of PLC is approximately 20 months, with a 5-year survival rate of only 10–13% [1,2]. Currently, PLC is ranked the fourth most common malignant tumor and is the second leading cause of cancer death in China. PLC mainly includes three different pathological types: hepatocellular carcinoma (HCC), intrahepatic cholangiocarcinoma (ICC), and combined hepatocellular-cholangiocarcinoma (cHCC-CCA), among which HCC is the most common subtype, accounting for approximately 75–85% of cases [3,4]. As a highly heterogeneous cancer, the occurrence and development of HCC is a complex process. Despite significant progress in the monitoring, diagnosis, and management of HCC, accurately predicting prognosis and treatment outcomes remains a challenge, and the exploration of new therapeutic targets and pathogenesis is urgent, among which research related to arachidonic acid metabolism holds great potential.

Arachidonic acid (AA), a polyunsaturated fatty acid, is widely present in the cell membranes of mammals, detaching upon the activation of phospholipase A2 by neural signals [5]. AA can be metabolized into active metabolites, such as hydroxyeicosapentaenoic acid, epoxy eicosatrienoic acid, and prostaglandins, through the cytochrome P450 (CYP450), lipoxygenase (LOX), and cyclooxygenase (COX) pathways [6–8]. It should be noted that when the lipoxygenase (LOX) and cyclooxygenase (COX) pathways are inhibited, AA is metabolized into lipoxin A4, which is an anti – inflammatory substance. This has also been involved in the research related to hepatocellular carcinoma (HCC). In addition, AA and its metabolites promote tumor development by regulating processes such as cell carcinogenesis, progression, and differentiation, including cell proliferation, chemotaxis, mitosis, migration, and apoptosis [8–10]. Studies have shown that the AA metabolism-related gene CYP4F2 is associated with the occurrence of HCC [11]. Therefore, arachidonic acid metabolism related genes (AAMRGs) may be involved in the occurrence and development of HCC. However, the specific mechanisms underlying their action require further research.

This study constructed a new prognostic model of arachidonic acid metabolism-related genes in hepatocellular carcinoma to predict the prognostic risk of patients with HCC, providing a basis for further research on the role of AA metabolism-related genes in the occurrence and development of HCC. In doing so, this work provides a new direction for clinical treatment of patients to support the prevention and treatment of HCC. In this study, bioinformatics techniques were used to screen prognosis genes related to AA metabolism in HCC and construct a prognostic model of HCC. At the same time, the model was evaluated and verified. The key factor KIF18A was screened out. By constructing KIF18A overexpression and knockdown cell lines, the effects on the invasion and migration of liver cancer cells and the epithelial-mesenchymal transition (EMT) were verified to elucidate the preliminary mechanism underlying the induction of abnormal proliferation and EMT of liver cancer cells by the KIF18A-mediated arachidonic acid-dependent pathway.

## Materials and methods

### Screening of DEG1 and DEG2

In the training set TCGA-HCC, with the conditions of adjusted P < 0.05 and |log2FC| > 1, differentially expressed genes (DEG) between normal samples and HCC patient samples were screened. The results were plotted as a volcano plot, and the expression heat map of the differential genes was displayed using the “pheatmap” package in R.

### Acquisition of candidate genes, enrichment analysis of candidate genes, and construction of PPI network

The differentially expressed genes between HCC and normal samples (DEG1), the differentially expressed genes in the high and low score groups of AAMGs (DEG2), and the WGCNA module genes were intersected. The genes obtained were denoted as the candidate genes for subsequent analysis. A total of 22 candidate genes were obtained, the results of which were plotted as a Venn diagram. For the intersection genes obtained in the previous section, GO and KEGG functional enrichments were performed using the “clusterProfiler” package in R, and the top-ranked results were displayed.

### Construction of candidate gene PPI network

Based on the STRING database, this study constructed and analyzed the protein–protein interaction network (PPI) of 22 candidate genes (interaction degree threshold: 0.4). Among them, 11 genes were outliers. As a result, interaction networks were constructed for 11 proteins, including the PPI network and interaction pairs, such as IGF2 BP1-IGF2 BP3, TRIP13-PIMREG, and G6PD-SPHK1. This network diagram contains 11 nodes and 11 edges.

### Univariate Cox regression analysis

Univariate Cox proportional hazards regression analysis was conducted on the survival information of the 22 candidate genes and their samples. The univariate cutoff was set to p < 0.05, and a total of 18 genes were screened. A total of 12 genes were screened after the PH (Proportional Hazards) assumption test, denoted as the significant results of the univariate analysis and plotted as a forest plot. To construct the prognostic model, based on the above univariate analysis results, multivariate Cox analysis was performed on the 12 genes and their expression levels and survival information. The step function was used to screen the model genes. The resulting prognostic model contained three genes (TRIP13, KIF18A, and PIMREG). The risk value of each patient was obtained as follows: riskScore = 0.45*TRIP13 + 0.56*KIF18A – 0.35*PIMREG.

The expression of the three prognostic genes in the HCC and normal groups in TCGA-HCC was compared, the results of which were plotted as box plots. The three prognostic genes were all significantly highly expressed in HCC samples (p < 0.05).

### Differential expression of KIF18A in clinical samples

In the online database of The Human Protein Atlas (HPA, https://www.proteinatlas.org/), search for the KIF18A gene. Select either normal tissues or tumor tissues, choose the specific category of normal or tumor tissues, and then view the online immunohistochemistry data.

### Cell culture

The human liver normal cell line THLE-2 and the human liver cancer cell lines HepG2, SNU423, JHH2, JHH7, and LI7 were purchased from Procell Life Science & Technology Co., Ltd. (Wuhan, China). The cells were cultured in DMEM medium containing 10% fetal bovine serum (G8003; Wuhan Servicebio Technology Co., Ltd, China) and 1% penicillin and streptomycin (G4003; Wuhan Servicebio Technology Co., Ltd, China), and placed in an incubator at 37 °C and 5% CO_2_ for culture. The cells were cultured until they grew to 90% confluence. The medium was removed, the cells were washed twice with PBS, 2 mL of trypsin (G4022; Wuhan Servicebio Technology Co., Ltd, China) was added for digestion. When the cells became round and detached, 2 mL of the medium containing serum was added to terminate the digestion. The cell suspension was transferred to a centrifuge tube, centrifuged (1000 × *g*, 5 min), the supernatant was discarded, and the cell pellet was collected for subsequent experiments.

### Cell transfection

The HepG2 and SNU423 cell pellets were collected and resuspended using 3 mL of medium. The cell density was calculated using a cell counter (SCC-M630; Wuhan Servicebio Technology Co., Ltd, China), and the cells were inoculated in a six-well plate at 1 × 10^5^ per well and incubated overnight. Two sterile 1.5 mL EP tubes were prepared respectively. In tube 1, 250 μL of serum-free medium was added, and then the interfering RNA sequence or overexpression vector was added. In tube 2, the same volume of serum-free medium and 500 μL of transfection reagent (G1802; Wuhan Servicebio Technology Co., Ltd, China) were added. The liquid in tube 2 was slowly dropped into tube 1, mixed gently, and incubated at room temperature for 20 min to form the transfection complex. After 48 h of transfection, HepG2 cells were divided into three groups: vector group (transfected with empty vector), oe-KIF18A group (overexpression vector), oe-KIF18A + U73122 group. Among them, the oe-KIF18A + U73122 group was added U73122 for continued incubation for 24 h after the transfection was completed. SNU423 cells were divided into three groups, including si-NC (transfected with ineffective sequence), si-KIF18–1 (transfected with interfering RNA sequence 1), and si-KIF18–2 (transfected with interfering RNA sequence 2).

### ELISA

The supernatant of each cell culture was aspirated and transferred to a centrifuge tube and centrifuged at 4 °C and 3000 × *g* for 10 min to remove cell debris and impurities. The resulting supernatant was collected, and the standard curve was prepared by diluting the standard product with the standard product diluent according to the ELISA kit instructions. Standard products, blank controls, and the supernatant of the samples to be tested were added to the corresponding wells. The plate was sealed with a sealing membrane and incubated in an incubator at 37 °C for 2 h. The sealing membrane was carefully removed, the liquid in the wells was discarded, and the wells were washed three times with the washing solution and patted dry. Diluted detection antibodies were added and the plates were sealed and incubated (as per to the requirements of the different kits). Then, the plates were washed, the substrate solution was added, and the plates were incubated in the dark until color had developed, after which a stop solution was added to stop the reaction. The absorbance (OD value) of each well was immediately measured using a microplate reader. Details of the ELISA protocol are provided in S1 Table.

### Western blotting

HepG2 and SNU423 cells were treated as previously described. Briefly, 3 mL of pre-cooled cell lysis buffer (G2002; Wuhan Servicebio Technology Co., Ltd, China) was added, and the cells were lysed on ice for 30 min. The cells were scraped off the wall of the culture flask with a cell scraper, and the lysate was collected in a centrifuge tube. The cells were centrifuged at 4 °C and 12000 × *g* for 15 min, and the supernatant was collected as the total protein of the cells. The protein concentration was determined according to the instructions of the BCA protein quantification kit (G2026; Wuhan Servicebio Technology Co., Ltd, China). According to the quantitative results, the protein sample concentrations of each group were adjusted to be consistent. Next, 40 μg of protein samples were added to each well for electrophoresis. Electrophoresis was performed at a constant voltage of 80 V until the samples entered the separation gel, and then the voltage was adjusted to 120 V for constant voltage electrophoresis until the bromophenol blue reached the bottom of the gel. After electrophoresis, the membrane was transferred at a constant current of 260 mA for 1 h. After the membrane transfer was completed, the membrane was placed in a blocking solution containing 5% skimmed milk powder and blocked on a shaker at room temperature for 2 h. The membrane was placed in the diluted primary antibody solution and incubated overnight on a shaker at 4 °C. The membrane was washed three times with TBST buffer for 10 min each time. Then, the membrane was placed in the diluted secondary antibody solution and incubated on a shaker at room temperature for 2 h. The excess liquid on the membrane was absorbed, and the chemiluminescence reagent was evenly dropped on the membrane for a reaction time of 2 min. Images were collected using a chemiluminescence imaging system (SCG-W3000; Wuhan Servicebio Technology Co., Ltd, China) to obtain the signal intensity of the protein bands. The gray value of the protein bands was quantitatively analyzed using image analysis software (ImageJ 1.52a). Antibody information and antibody dilution ratios are provided in S2 Table.

### RT-qPCR

The total RNA of treated HepG2 and SNU423 cells was extracted using RNA extraction kits (G3013; Wuhan Servicebio Technology Co., Ltd, China). The operation was carried out according to the kit instructions. After the extraction was completed, the concentration and purity of RNA were determined using a NanoDrop spectrophotometer (Thermo Fisher Scientific, China). The extracted RNA was reverse transcribed into cDNA using the reverse transcription kit name (G3332; Wuhan Servicebio Technology Co., Ltd, China). The reaction system included RNA templates, random primers, reverse transcriptase, and dNTPs. The reaction conditions were set according to the kit instructions, and the reverse transcription reaction was performed on a PCR instrument. RT-qPCR reactions were performed on a CFX Opus Real-Time PCR Systems (Bio-Rad Laboratories, USA) using the Universal One-Step RT-qPCR Kit (G3345; Wuhan Servicebio Technology Co., Ltd, China). The reaction system included cDNA templates, upstream and downstream primers, SYBR Green fluorescent dye, and PCR buffer. The reaction was performed under the following conditions: pre-denaturation at 95 °C for 30 s; denaturation at 95 °C for 5 s, annealing at 55 °C for 30 s, for a total of 40 cycles. Details of the RT-qPCR sequences are provided in S3 Table.

### Cell scratch assay

The cells were counted and inoculated in a six-well plate at a density of 1 × 10^6^. Briefly, 2 mL of medium was added to each well to evenly distribute the cells, followed by incubation overnight to form a monolayer of cells. On the following day, a 10 μL sterile pipette tip was used to make a vertical scratch on the monolayer of cells, ensuring that the scratch width was consistent. The cells were gently washed 3 times with PBS to remove the scratched cell debris, and serum-free medium was added. The six-well plate was placed in an incubator for continued culture, and observations and photographs were taken under an inverted microscope at 0 h and 48 h, respectively. The width of the scratch at different time points was measured using ImageJ 1.5.2a software, and the cell migration rate was calculated.

### Transwell assay

The matrix gel (G4130; Wuhan Servicebio Technology Co., Ltd, China) was stored overnight in a refrigerator at 4 °C and diluted 10 times with serum-free medium. Next, 100 μL of the diluted matrix gel were added to the upper chamber of the Transwell chamber before placing it in an incubator for incubation for 4 h to solidify the matrix gel. The cells were treated as required by different groups, and the cell concentration was adjusted to 5 × 10⁵ cells/mL. Then, 200 μL of the cell suspension was added to the upper chamber, and 600 μL of the medium containing 20% FBS was added to the lower chamber as the chemotactic factor. The Transwell plate was placed in an incubator for 48 h. The Transwell chamber was removed, and the non-invasive cells in the upper chamber were gently wiped off with a cotton swab. The invasive cells in the lower chamber were fixed with 4% paraformaldehyde (GC1101; Wuhan Servicebio Technology Co., Ltd, China) for 20 min, followed by staining with 0.1% crystal violet (GC307002; Wuhan Servicebio Technology Co., Ltd, China) for 20 min. The cells were washed with PBS, and the number of invasive cells was counted in five randomly selected fields under a microscope. The number of invasive cells was calculated in Image J 1.52a.

### Immunofluorescence assay

Cells were treated, counted, and inoculated in six-well plates at a density of 1 × 10^5^. Briefly, 2 mL of medium was added to each well and placed in an incubator for overnight culture. The medium was aspirated, and the cells were gently washed twice with PBS. An appropriate amount of 4% paraformaldehyde was added and fixed at room temperature for 20 min. The cells were washed three times with PBS for 5 min each time. Next, 0.2% Triton X-100 was added and incubated at 25 °C for 10 min, followed by the addition of blocking solution. After incubation for 1 h, the blocking solution was removed and anti-E-cadherin and anti-N-cadherin antibodies (1:100) were added, followed by incubation overnight at 4 °C. On the following day, the cells were washed three times with PBS for 5 min each time. The corresponding fluorescently labeled secondary antibody (operation in the dark) was added and incubated for 1 h. DAPI staining solution was added and incubated in the dark for 10 min. The cells were washed three times with PBS for 5 min each time. The double staining of E-cadherin and DAPI, and N-cadherin and DAPI was observed using a fluorescence microscope, and images were collected. Details on the antibodies used is provided in S2 Table.

### Statistical analysis

Data processing and statistical analysis were performed in R (version 4.3.2), using the relevant software packages to obtain statistical graphs. The Wilcoxon test was used to compare the differences between two groups, and the t-test was used for the comparison between continuous variables. Survival analysis was performed using Kaplan-Meier analysis, and the difference analysis was performed using the Log-rank test. For *in vitro* cell experiments, all data were evaluated using one-way ANOVA, followed by post-hoc comparisons using Tukey’s honest significant difference test. P < 0.05 indicated statistically significant differences. All experiments were repeated more than three times.

## Results

### Screening of differentially expressed genes and key module genes of WGCNA

**Fig 1:**
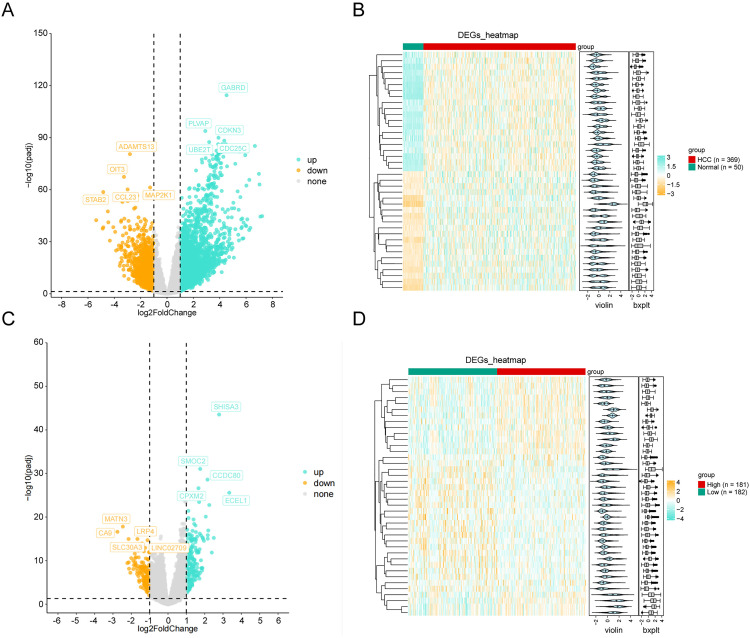
Differential gene analysis of hepatocellular carcinoma in the GSE136247 data set. (A) Volcano plot of the DEG1 dataset. Orange, gray, and green dots represent upregulated, stable genes, and downregulated genes, respectively. (B) Heat map of DEG1. Each column represents a sample, and each row represents the expression level of a gene sample (showing the top 40 genes with |FC|). Green and red denote the control and disease groups, respectively. The darker the color, the higher the expression level; the violin plots and box plots show the expression of genes in different samples. (C) Volcano plot of the DEG2 dataset. The orange dots represent upregulated genes, the gray dots represent stable genes, and the green dots represent downregulated genes. (D) Heat map of DEG2. Each column represents a sample, and each row represents the expression level of a gene sample (showing the top 40 genes with |FC|). Green and red denote the control and disease groups, respectively. The darker the color, the higher the expression level; the violin plots and box plots show the expression of genes in different samples.

**Fig 2:**
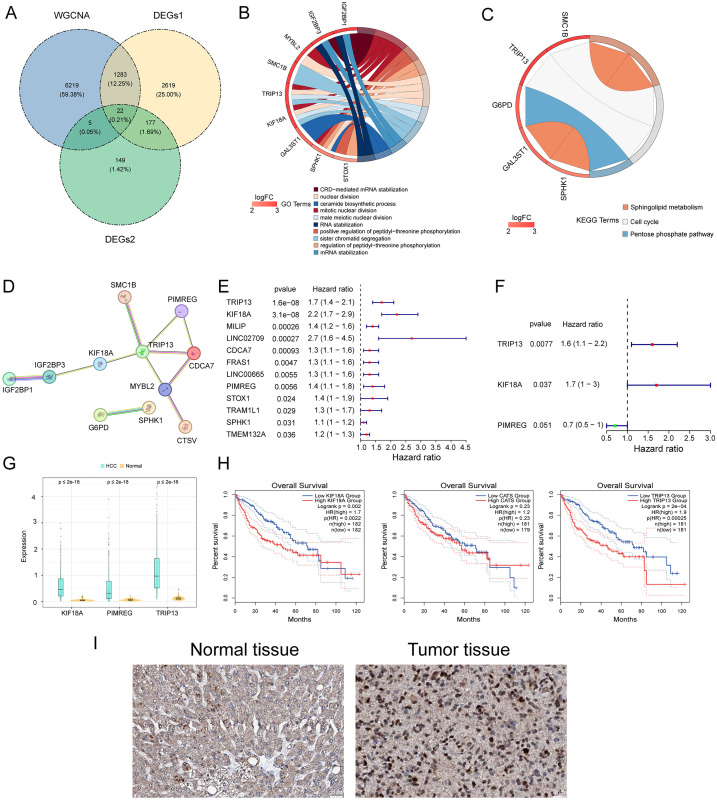
Potential target gene prediction for PHI treatment in HCC. (A) Venn diagram of candidate genes. (B) GO enrichment analysis of candidate genes. (C) KEGG enrichment analysis of candidate genes. (D) PPI network of candidate genes. (E) Forest plot for univariate Cox analysis. The left represents genes and corresponding P and HR values; the red squares on the right indicate HR values greater than 1, and the line segments on either side of the squares are the 95% confidence intervals. (F) Forest plot for multivariate Cox analysis. The left represents the genes and the corresponding P and HR values; the red squares on the right indicate HR values greater than 1, the green squares indicate that the HR value is less than 1, and the line segments on either side of the squares are the 95% confidence intervals for that HR value. (G) Boxplots of the expression levels of the three prognostic genes. (H) Prognostic survival curve analysis of candidate genes in the liver cancer ATGC cohort. (I) The differential expression of KIF18A between normal tissues and liver cancer tumors was observed through the HPA database.

A total of 4,101 DEG1 genes were identified (Fig 1A). Among them, 2,912 were upregulated and 1,189 were downregulated differentially expressed genes. A heat map of the differential gene clustering is provided in Fig 2B. A total of 353 DEG2 genes were obtained, and the results were plotted as a volcano plot (Fig 2C). Among them, 196 were upregulated and 157 were downregulated differentially expressed genes. The heat map of differential gene clustering is shown in detail in Fig 2D. In the training set TCGA-HCC, a co-expression network of WGCNA was constructed. Firstly, the samples were clustered to obtain the sample clustering diagram (S1A Fig). This demonstrated that the general clustering effect of the samples was good with no obvious outlier samples. Thus, no samples needed to be excluded. Then, to ensure that the interaction between genes conformed to the scale-free distribution to the maximum extent, the soft threshold of the data was determined (S1B Fig). The horizontal axes of S1 Fig represent the weight parameters. The vertical axis of S1A Fig represents the scale-free topology model fit, that is, the signed R^2^, which is the square of the correlation coefficient between log(k) and log(p(k)) in the corresponding network. The higher the square of the correlation coefficient, the closer the network was to the scale-free distribution. Generally, R^2^ was selected at 0.85. The vertical axis in S1B Fig represents the average of all gene adjacency functions in the corresponding gene module. Based on the position of the red line and the two indicators of average connectivity, the soft threshold was determined to be 13, that is, the network approximated the scale-free distribution at this time (S1B Fig). Based on the optimal soft threshold, the minimum number of genes in each gene module was set to 100, according to the standard of the mixed dynamic shear tree algorithm (dynamic tree cutting), and a total of 16 modules were segmented to obtain the module clustering tree diagram (S1C Fig). Then, the correlation between each module and the HCC trait was calculated (S1D Fig). Among these 16 modules, the blue (R^2^ = 0.57, P < 0.05) and turquoise modules (R^2^ = 0.51, P < 0.05) showed significantly positive correlations with HCC. Therefore, the turquoise and blue modules were identified as key gene modules. Among them, the turquoise module contained 6,194 genes, while the blue module contained 1,335 genes. After merging, 7,529 key module genes related to the HCC phenotype could be obtained.

### Acquisition of candidate genes, enrichment analysis of candidate genes, and construction of PPI network

The differentially expressed genes between HCC and normal samples (DEG1), the differentially expressed genes in the high and low score groups of AAMGs (DEG2), and the WGCNA module genes were intersected in this study. The genes obtained were denoted as candidate genes for subsequent analysis, and a total of 22 candidate genes were obtained, the results of which were plotted as a Venn diagram (Fig 2A). In terms of biological processes, genes were found to be significantly enriched in pathways such as CRD-mediated mRNA stabilization, mitotic nuclear division, and positive regulation of peptidyl-threonine phosphorylation (Fig 2B). In this study, the screening condition of KEGG enrichment analysis was p < 0.05, the results of which are shown in Fig 2C. Candidate genes were found to be significantly enriched in pathways such as sphingolipid metabolism, cell cycle, and pentose phosphate pathway. Based on the STRING database, this study constructed and analyzed the protein–protein interaction network (PPI) of 22 candidate genes, including the PPI network, with interaction pairs such as IGF2 BP1-IGF2 BP3, TRIP13-PIMREG, and G6PD-SPHK1 (Fig 2D). A total of 12 genes were screened after univariate Cox regression analysis (Fig 2E). Multivariate Cox regression analysis (Fig 2F) was comprised of three genes (TRIP13, KIF18A, and PIMREG) in the prognostic model. In TCGA-HCC, compared with the normal group, the relative expression levels of the KIF18A, PIMREG, and TRIP3 genes in the HCC group were all higher (Fig 2G), and the prognosis was worse (Fig 2H). The immunohistochemistry results of KIF18A from the HPA database verified the differential expression of KIF18A between normal tissues and liver cancer tumors. Specifically, the expression level of KIF18A was higher in tumor tissues than in normal tissues (Fig 2I).

### KIF18A overexpression induces liver cancer cell proliferation

**Fig 3:**
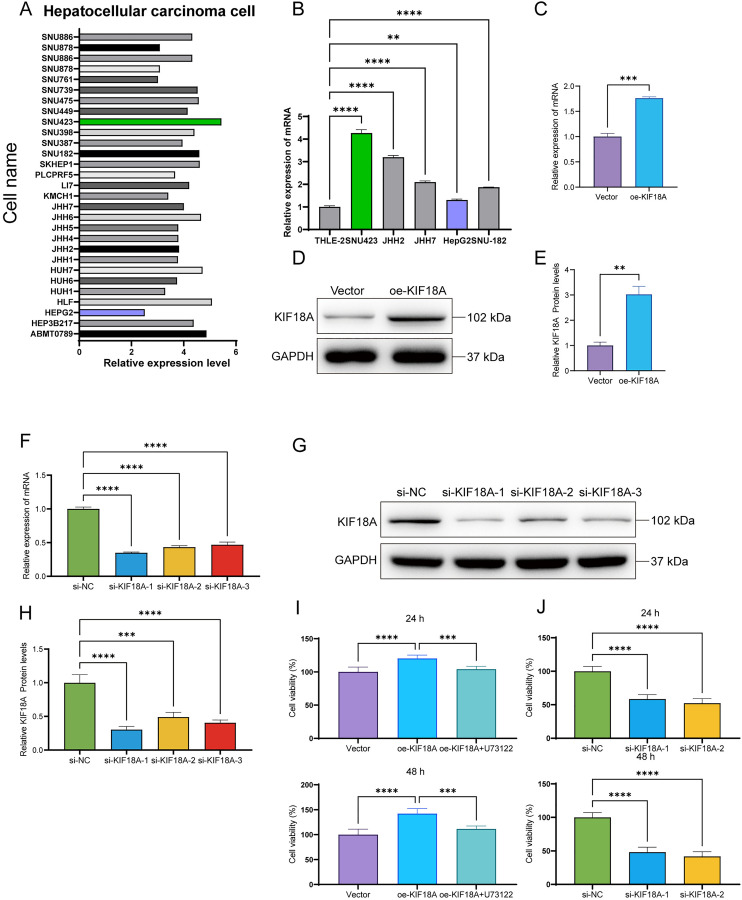
Screening and construction of liver cancer cell lines. **(A)** Search for the relative expression levels of different KIF18A in liver cancer cells in the online database (https://sites.broadinstitute.org/ccle/). **(B)** The relative expression levels of mRNA in normal liver cell THLE-2 and liver cancer cells SNU423, JHH2, JHH7, HepG2, and LI7 were detected by RT-qPCR. **(C)** After transfection with the overexpression KIF18A vector for 48 h in liver cancer HepG2 cells, the relative expression level of KIF18A mRNA was detected by RT-qPCR. **(D)** The relative expression level of KIF18A protein was detected by western blotting in HepG2 cells. **(E)** Column diagram of the relative expression level of KIF18A in HepG2 cells compared to GAPDH by western blotting. **(F)** After transfection with the overexpression KIF18A vector for 48 h in liver cancer SNU423 cells, the relative expression level of KIF18A mRNA was detected by RT-qPCR. **(G)** Column diagram of the relative expression level of KIF18A compared to GAPDH by western blotting. **(H)** Column diagram of the relative expression level of KIF18A in SNU423 cells compared to GAPDH by western blotting. **(I)** Changes in cell viability were detected by CCK8 after transfection with the KIF18A overexpression vector in HepG2 cells and addition of U73122 after incubation for 24 h. **(J)** Changes in cell viability were detected by CCK8 after transfection with the KIF18A interfering RNA in SNU423 cells. (Compared between the two groups, * P < 0.05, ** P < 0.01, *** P < 0.001, **** P < 0.0001).

Using the CCLE (Cancer Cell Line Encyclopedia) database, the expression of KIF18A in different liver cancer cell lines was screened (Fig 3A). A total of five liver cancer and normal liver cells were used to detect the KIF18A mRNA expression levels using RT-qPCR. Among the liver cancer cells, the expression of KIF18A mRNA was highest in SNU423 and lowest in HepG2 compared with normal liver cells, consistent with the prediction results of the CCLE database (Fig 3B). Therefore, a KIF18A overexpression cell line was established in HepG2 with low levels of KIF18A expression, and a KIF18A knockdown cell line was constructed in SNU423 cells with high KIF18A expression. The expression levels of KIF18A protein and mRNA were detected by western blotting and RT-qPCR, confirming the successful construction of the cell model (Fig 3C–H). All three siRNA interference sequences showed a good knockdown performance. In subsequent experiments, siRNA1 and siRNA2 were selected. To confirm the effect of KIF18A on the proliferation ability of liver cancer cells, changes in cell viability were detected using CCK8 assays. The results showed that the viability of HepG2 cells was significantly enhanced after KIF18 overexpression, and that U73122 could counteract abnormal proliferation (Fig 3I). When the KIF gene was knocked down, the survival rate of SNU423 cells decreased significantly. This indicates that KIF18A is an oncogene that promotes cell proliferation (Fig 3J).

### KIF18A overexpression induces the invasion and migration of liver cancer cells

**Fig 4:**
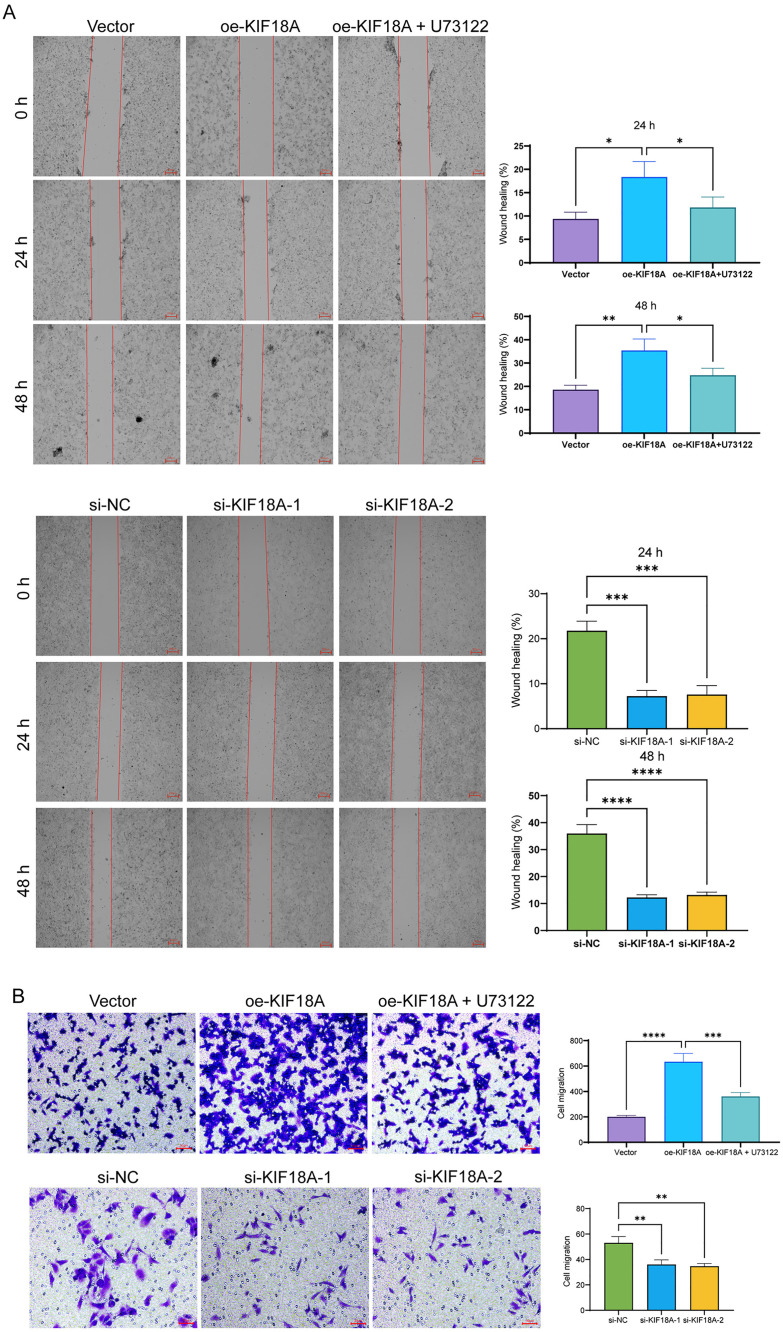
KIF18A can induce invasion and migration of liver cancer cells. (A) Transfection with KIF18 overexpression vector and interfering RNA, and U73122 was added after overexpression vector transfection. Changes in the migration ability of liver cancer cells HepG2 and SNU423 were detected by cell scratch assay. (B) Changes in the invasion ability of liver cancer cells HepG2 and SNU423 were detected by Transwell assay. (Compared between the two groups, * P < 0.05, ** P < 0.01, *** P < 0.001, **** P < 0.0001).

After confirming the effect of KIF18A on cell proliferation ability of liver cancer cells, whether it also affected cell invasion and migration ability was evaluated. The results of the cell scratch and Transwell assays indicated that KIF18A could induce an enhanced migration and invasion ability in HepG2 cells, which was counteracted by the addition of U73122. KIF18A knockdown significantly weakened the invasion and migration ability of SNU423 cells (Fig 4A and 4B). In conclusion, KIF18A can induce the invasion and migration of liver cancer cells.

### KIF18A overexpression promotes the EMT of liver cancer cells

**Fig 5:**
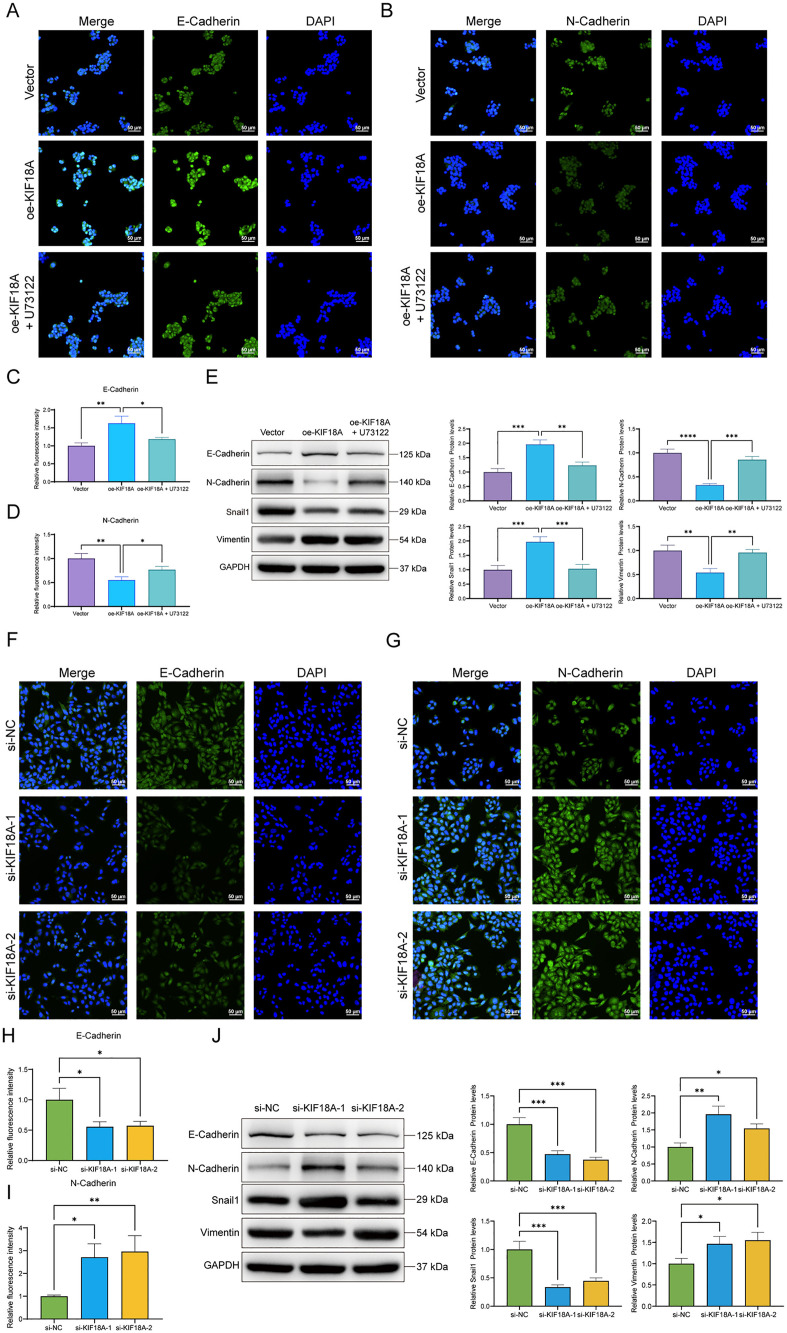
KIF18A can inhibit the EMT. (A) Detection of the localization and expression level changes of E-cadherin in HepG2 cells by immunofluorescence. (B) Detection of the localization and expression level changes of N-cadherin in HepG2 cells by immunofluorescence. (C) Relative expression level of E-cadherin relative to DAPI fluorescence intensity. (D) Relative expression level of N-cadherin relative to DAPI fluorescence intensity. (E) Detection of the relative expression levels of E-cadherin, N-cadherin, Snail1, and Vimentin proteins in HepG2 cells by western blotting. (F) Detection of the localization and expression level changes of E-cadherin in SNU423 cells by immunofluorescence. (G) Detection of the localization and expression level changes of N-cadherin in SNU423 cells by immunofluorescence. (H) Relative expression level of E-cadherin relative to DAPI fluorescence intensity. (I) Relative expression level of N-cadherin relative to DAPI fluorescence intensity. (J) Detection of the relative expression levels of E-cadherin, N-cadherin, Snail1, and Vimentin proteins in SNU423 cells by western blotting. (Compared between the two groups, * P < 0.05, ** P < 0.01, *** P < 0.001, **** P < 0.0001).

To confirm the role of KIF18A in the EMT of liver cancer cells, the key indicators of EMT, including E-cadherin, N-cadherin, Snail1, and Vimentin, were evaluated using immunofluorescence and western blotting. The results of the assays showed that KIF18A downregulated the relative expression levels of E-cadherin protein and upregulated that of N-cadherin protein, while U73122 reversed this effect. After the knockdown of KIF18A, the relative expression level of E-cadherin protein was upregulated, while that of N-cadherin protein was downregulated. In addition, the western blotting results also showed that KIF18A could downregulate the protein expression level of Snail1 and upregulate that of Vimentin, whose regulatory effect was flipped after knockdown (Fig 5A–J).

### KIF18A overexpression inhibits the 5-LOX-dependent arachidonic acid pathway

**Fig 6:**
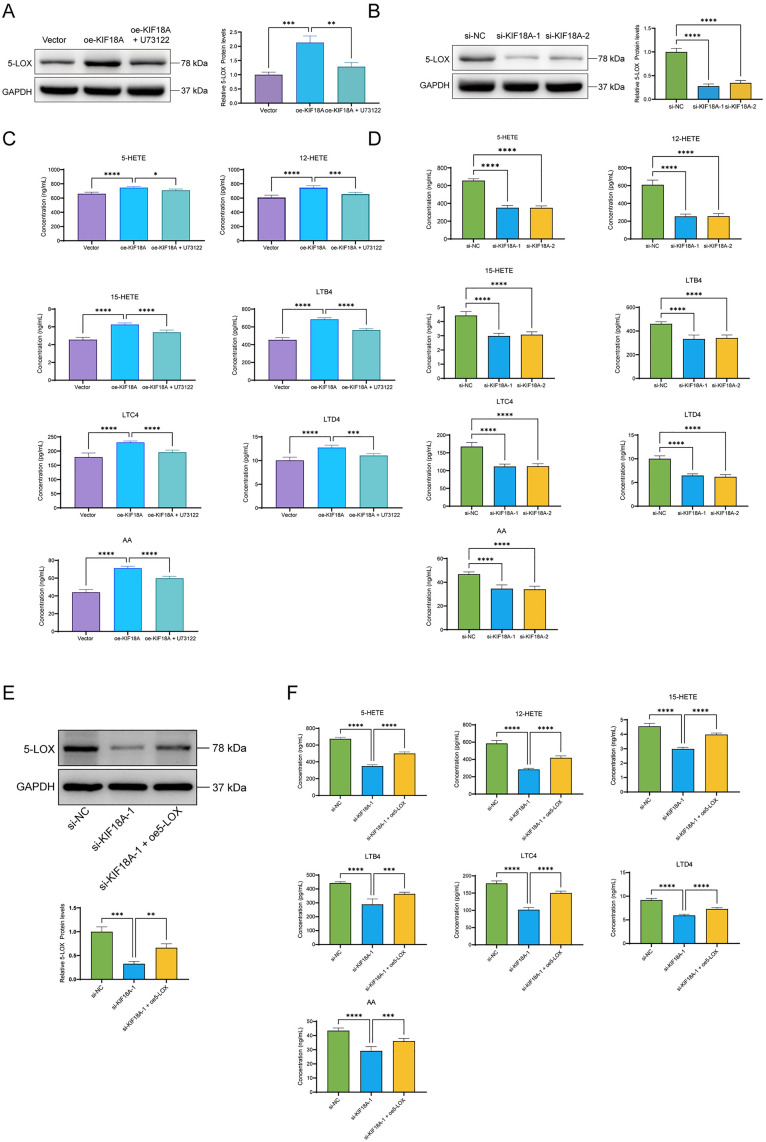
KIF18A can regulate the arachidonic acid pathway. (A) Detection of the relative expression level of 5-LOX protein in HepG2 cells by western blotting. (B) Detection of the relative expression level of 5-LOX protein in SNU423 cells by western blotting. (C) Relative expression levels of 5-HETE, 12-HETE, 15-HETE, LTB4, LTC4, LTD4, and AA in the cell supernatant of HepG2 cells were detected by ELISA. (D) Relative expression levels of 5-HETE, 12-HETE, 15-HETE, LTB4, LTC4, LTD4, and AA in the cell supernatant of SNU423 cells were detected by ELISA. (E) In SNU423 cells, after knocking down KIF18A, 5 – LOX was overexpressed, and the change in the expression level of 5 – LOX protein was detected by Western blot assay. (F) In SNU423 cells, following the knockdown of KIF18A and subsequent overexpression of 5 – LOX, the relative expression levels of 5 – HETE, 12 – HETE, 15 – HETE, LTB4, LTC4, LTD4 and AA in the supernatant of SNU423 cells were measured by ELISA.(Compared between the two groups, * P < 0.05, ** P < 0.01, *** P < 0.001, **** P < 0.0001).

To investigate whether the effect of KIF18A on the proliferation, invasion, migration, and EMT of liver cancer cells was related to the 5-LOX-dependent arachidonic acid pathway, the relative expression of key factors was evaluated using western blotting and ELISA. The western blotting results indicated that KIF18A induced an increase in the level of 5-LOX, an effect that was weakened after the addition of U73122. The level of 5-LOX protein also decreased after KIF18 knockdown (Fig 6A and 6B). In addition, the ELISA results showed that KIF18A upregulated the relative expression levels of 5-HETE, 12-HETE, 15-HETE, LTB4, LTC4, LTD4, and AA. U73122 was found to restore the expression levels of these factors. The expression levels of the above factors were also decreased after KIF18A knockdown (Fig 6C and 6D). To further elucidate the regulatory relationship between KIF18A and 5-LOX and its signaling pathway, we established a KIF18A knockdown model in SNU423 cells and transfected a 5-LOX overexpression plasmid in this model for a rescue experiment. By detecting the changes in the protein level of 5-LOX and the expression of downstream related factors in its pathway (5-HETE, 12-HETE, 15-HETE, LTB4, LTC4, LTD4 and AA), we deeply explored the functional connection between them. The results showed that knockdown of KIF18A significantly downregulated the expression of 5-LOX protein and decreased the levels of the above-mentioned pathway-related factors. However, after overexpressing 5-LOX in KIF18A-knockdown cells, the inhibitory effect of KIF18A deficiency on the 5-LOX pathway was effectively eliminated (Fig 6E and 6F). The above results indicate that KIF18A can upregulate the levels of 5-LOX and factors related to its pathway, including 5-HETE, 12-HETE, 15-HETE, LTB4, LTC4, LTD4, and AA, thus inducing the activation of the arachidonic acid pathway. Notably, pretreatment with the specific 5-LOX inhibitor U73122 can effectively counteract the regulatory effect of the KIF18A protein on the 5-LOX pathway. In summary, these findings suggest that the regulatory effect of KIF18A on the progression of liver cancer cells may be mediated through the 5-LOX-dependent arachidonic acid pathway.

## Discussion

This study evaluated the role of KIF18A in liver cancer via the 5-LOX-dependent arachidonic acid pathway, a perspective scarcely explored in the literature. Although previous studies have shown that arachidonic acid metabolism-related genes may be involved in the occurrence and development of HCC, the specific role of KIF18A in this pathway has yet to be fully elucidated. First, through bioinformatics analysis and experimental verification, the expression level of KIF18A in liver cancer cells was found to be significantly higher than that in normal liver cells, with high levels of KIF18A expression associated with a poor prognosis in patients with liver cancer. This finding suggests that KIF18A may play an important role in the occurrence and development of liver cancer. Further cell function experiments showed that KIF18A overexpression could significantly promote the proliferation, invasion, and migration ability of liver cancer cells, while KIF18A knockdown inhibited these cell behaviors, confirming that KIF18A is an oncogene, which is consistent with previous results [12,13]. Secondly, this study also found that KIF18A could promote the EMT of liver cancer cells. EMT is a key step in tumor invasion and metastasis, characterized by the downregulation of epithelial cell marker E-cadherin and the upregulation of mesenchymal cell marker N-cadherin [14,15]. The immunofluorescence and western blotting results showed that KIF18A overexpression downregulated the expression of E-cadherin and upregulated N-cadherin expression, while the 5-LOX inhibitor U73122 reversed this effect, indicating that KIF18A may promote the invasion and migration of liver cancer cells by regulating the expression of EMT-related proteins.

Additionally, the role of KIF18A in the arachidonic acid metabolism pathway was also explored. Arachidonic acid is a polyunsaturated fatty acid in the cell membrane whose metabolites play an important role in tumor development. 5-Lipoxygenase (5-LOX) is a core enzyme in the arachidonic acid (AA) metabolic pathway, which converts AA into various hydroxy-eicosatetraenoic acids (HETEs), including 5-HETE, 12-HETE, and 15-HETE [16–18]. These metabolites are further converted into biologically active leukotrienes, such as LTB4, LTC4, and LTD4, which play a key role in regulating inflammatory responses and cell signaling [19,20]. 5-LOX converts AA into 5-HPETE, and then generates LTA4, as a precursor for leukotriene synthesis. LTB4 is directly generated from LTA4, while LTC4, LTD4, and LTE4 require the action of LTA4 and other enzymes [21]. It should be specifically pointed out that when the metabolic pathways of LOX and COX are inhibited, AA is metabolized into lipoxin A4, an anti – inflammatory substance, which in turn affects the progression of liver cancer [8–10]. LTB4 can attract white blood cells, while LTC4, LTD4, and LTE4 affect smooth muscle contraction and inflammation by binding to specific receptors. The release and metabolism of AA are regulated by enzymes of the phospholipase A2 (PLA2) family, which are responsible for releasing AA from the phospholipids of the cell membrane [22,23]. The regulatory network of the 5-LOX pathway is very complex and involves the interaction of multiple enzymes and signaling molecules, including the activation and scaffolding effect of 5-LOX activating protein (FLAP) on 5-LOX [24]. Our results indicated that KIF18A induced the upregulation of 5-LOX expression and increased the arachidonic acid metabolism. These results suggest that KIF18A promotes the proliferation, invasion, and EMT of liver cancer cells by activating the 5-LOX-dependent arachidonic acid pathway.

Although these results are promising, it is noteworthy that the conclusion regarding the clinical relevance of the expression characteristics of KIF18A in liver cancer in this study is mainly based on the immunohistochemical analysis of the HPAdatabase. Although the HPA data cover a large number of clinical samples, it is difficult to control confounding factors. The differences in the interpretation of immunohistochemical staining intensity across different centers may affect the reliability of the results. In the future, we will collect relevant clinical samples to further validate our conclusions. Moreover, due to the lack of in-vivo animal experiments, we are unable to evaluate the systemic therapeutic effects of targeting this pathway. To overcome these limitations, future research will involve constructing patient-derived xenograft models of liver cancer and conditional gene-knockout mice. We will also use an siRNA nano-delivery system in combination with 5-LOX inhibitors to verify the in-vivo efficacy. Finally, by integrating single-cell transcriptomics and spatial metabolomics, we aim to elucidate the spatio-temporal regulatory mechanisms of this pathway within the tumor niche, providing a translational experimental basis for the targeted treatment of liver cancer.

## Conclusion

This study reveals that KIF18A promotes the proliferation, invasion, migration, and EMT of liver cancer cells by activating the 5-LOX-dependent arachidonic acid pathway, with high KIF18A expression associated with a poor prognosis in patients with liver cancer. These findings provide insights that support the development of new approaches for the prognostic evaluation and targeted therapy of liver cancer.

## Supporting information

S1 Fig Identification of key module genes in WGCNA. (DOCX)

S1 Table ELISA kit information. (DOCX)

S2 Table Antibody information used in this study. (DOCX)

S3 Table RT-qPCR primer information and sequence information related to knockdown and overexpression. (DOCX)

S1 Raw Images The original image of uncropped western blot. (PDF)

## References

[1] Bray F, Laversanne M, Sung H, Ferlay J, Siegel RL, Soerjomataram I, et al. Global cancer statistics 2022: GLOBOCAN estimates of incidence and mortality worldwide for 36 cancers in 185 countries. CA Cancer J Clin. 2024;74(3):229–63. doi: 10.3322/caac.21834 38572751

[2] Wang S, Zheng R, Li J, Zeng H, Li L, Chen R, et al. Global, regional, and national lifetime risks of developing and dying from gastrointestinal cancers in 185 countries: a population-based systematic analysis of GLOBOCAN. Lancet Gastroenterol Hepatol. 2024;9(3):229–37. doi: 10.1016/S2468-1253(23)00366-7 38185129 PMC10849975

[3] Li M, Hu M, Jiang L, Pei J, Zhu C. Trends in Cancer Incidence and Potential Associated Factors in China. JAMA Netw Open. 2024;7(10):e2440381. doi: 10.1001/jamanetworkopen.2024.40381 39432306 PMC11581522

[4] Yu Z, Bai X, Zhou R, Ruan G, Guo M, Han W, et al. Differences in the incidence and mortality of digestive cancer between Global Cancer Observatory 2020 and Global Burden of Disease 2019. Int J Cancer. 2024;154(4):615–25. doi: 10.1002/ijc.34740 37750191

[5] Wang B, Wu L, Chen J, Dong L, Chen C, Wen Z, et al. Metabolism pathways of arachidonic acids: mechanisms and potential therapeutic targets. Signal Transduct Target Ther. 2021;6(1):94. doi: 10.1038/s41392-020-00443-w 33637672 PMC7910446

[6] Clemente SM, Martínez-Costa OH, Monsalve M, Samhan-Arias AK. Targeting Lipid Peroxidation for Cancer Treatment. Molecules. 2020;25(21):5144. doi: 10.3390/molecules25215144 33167334 PMC7663840

[7] Sharma V, Bhatia P, Alam O, Javed Naim M, Nawaz F, Ahmad Sheikh A, et al. Recent advancement in the discovery and development of COX-2 inhibitors: Insight into biological activities and SAR studies (2008-2019). Bioorg Chem. 2019;89:103007. doi: 10.1016/j.bioorg.2019.103007 31132600

[8] Iida N, Sugiyama A, Myoubudani H, Inoue K, Sugamata M, Ihara T, et al. Suppression of arachidonic acid cascade-mediated apoptosis in aflatoxin B1-induced rat hepatoma cells by glucocorticoids. Carcinogenesis. 1998;19(7):1191–202. doi: 10.1093/carcin/19.7.1191 9683177

[9] Panigrahy D, Greene ER, Pozzi A, Wang DW, Zeldin DC. EET signaling in cancer. Cancer Metastasis Rev. 2011;30(3–4):525–40. doi: 10.1007/s10555-011-9315-y 22009066 PMC3804913

[10] Yarla NS, Bishayee A, Sethi G, Reddanna P, Kalle AM, Dhananjaya BL, et al. Targeting arachidonic acid pathway by natural products for cancer prevention and therapy. Semin Cancer Biol. 2016;40–41:48–81. doi: 10.1016/j.semcancer.2016.02.001 26853158

[11] Teng L, Li Z, Shi Y, Gao Z, Yang Y, Wang Y, et al. Development and validation of a microenvironment-related prognostic model for hepatocellular carcinoma patients based on histone deacetylase family. Transl Oncol. 2022;26:101547. doi: 10.1016/j.tranon.2022.101547 36191460 PMC9531286

[12] Mohd Amin AS, Eastwood S, Pilcher C, Truong JQ, Foitzik R, Boag J, et al. KIF18A inhibition: the next big player in the search for cancer therapeutics. Cancer Metastasis Rev. 2024;44(1):3. doi: 10.1007/s10555-024-10225-3 39580563

[13] Gliech CR, Yeow ZY, Tapias-Gomez D, Yang Y, Huang Z, Tijhuis AE, et al. Weakened APC/C activity at mitotic exit drives cancer vulnerability to KIF18A inhibition. EMBO J. 2024;43(5):666–94. doi: 10.1038/s44318-024-00031-6 38279026 PMC10907621

[14] Xue W, Yang L, Chen C, Ashrafizadeh M, Tian Y, Sun R. Wnt/β-catenin-driven EMT regulation in human cancers. Cell Mol Life Sci. 2024;81(1):79. doi: 10.1007/s00018-023-05099-7 38334836 PMC10857981

[15] Yang S, Zhang D, Sun Q, Nie H, Zhang Y, Wang X, et al. Single-Cell and Spatial Transcriptome Profiling Identifies the Transcription Factor BHLHE40 as a Driver of EMT in Metastatic Colorectal Cancer. Cancer Res. 2024;84(13):2202–17. doi: 10.1158/0008-5472.CAN-23-3264 38657117

[16] Alam W, Khan H, Jan MS, W Darwish H, Daglia M, A Elhenawy A. In vitro 5-LOX inhibitory and antioxidant potential of isoxazole derivatives. PLoS One. 2024;19(10):e0297398. doi: 10.1371/journal.pone.0297398 39365759 PMC11452043

[17] Wang J, Li L, Chen P, He C, Niu X, Mei Q. Homocysteine aggravates intestinal inflammation through promotion of 5-LOX and COX-2 in IBD. Eur J Med Res. 2024;29(1):537. doi: 10.1186/s40001-024-02125-7 39506850 PMC11542312

[18] Cerchia C, Küfner L, Werz O, Lavecchia A. Identification of selective 5-LOX and FLAP inhibitors as novel anti-inflammatory agents by ligand-based virtual screening. Eur J Med Chem. 2024;263:115932. doi: 10.1016/j.ejmech.2023.115932 37976708

[19] Zhang Z, Zhao C, Sun L, Cheng C, Tian Q, Wu C, et al. Trappc1 intrinsically prevents ferroptosis of naive T cells to avoid spontaneous autoinflammatory disease in mice. Eur J Immunol. 2024;54(3):e2350836. doi: 10.1002/eji.202350836 38234007

[20] Zeng W, Xia J, Zeng Q. Levels of serum inflammatory cytokines and their correlations with disease severity in patients with chronic spontaneous urticaria. Postepy Dermatol Alergol. 2024;41(1):85–90. doi: 10.5114/ada.2024.135922 38533368 PMC10962375

[21] Wu Z, Mehrabi Nasab E, Arora P, Athari SS. Study effect of probiotics and prebiotics on treatment of OVA-LPS-induced of allergic asthma inflammation and pneumonia by regulating the TLR4/NF-kB signaling pathway. J Transl Med. 2022;20(1):130. doi: 10.1186/s12967-022-03337-3 35296330 PMC8925173

[22] Choi JM, Baek SE, Kim JO, Jeon EY, Jang EJ, Kim CD. 5-LO-derived LTB4 plays a key role in MCP-1 expression in HMGB1-exposed VSMCs via a BLTR1 signaling axis. Sci Rep. 2021;11(1):11100. doi: 10.1038/s41598-021-90636-2 34045591 PMC8160259

[23] Archambault A-S, Brassard J, Bernatchez É, Martin C, Di Marzo V, Laviolette M, et al. Human and Mouse Eosinophils Differ in Their Ability to Biosynthesize Eicosanoids, Docosanoids, the Endocannabinoid 2-Arachidonoyl-glycerol and Its Congeners. Cells. 2022;11(1):141. doi: 10.3390/cells11010141 35011703 PMC8750928

[24] Kretzer C, Jordan PM, Bilancia R, Rossi A, Gür Maz T, Banoglu E, et al. Shifting the Biosynthesis of Leukotrienes Toward Specialized Pro-Resolving Mediators by the 5-Lipoxygenase-Activating Protein (FLAP) Antagonist BRP-201. J Inflamm Res. 2022;15:911–25. doi: 10.2147/JIR.S345510 35173459 PMC8842732

