# Supplementary material for: KIF18A induces the EMT process of hepatoma cells through the 5-LOX-dependent arachidonic acid pathway
Source: PLoS One. 2025 Oct 13;20(10):e0333385. doi: 10.1371/journal.pone.0333385 (PMC12517525; doi:10.1371/journal.pone.0333385)
Supplement: S1 Fig — (DOCX) [file pone.0333385.s001.docx]

**
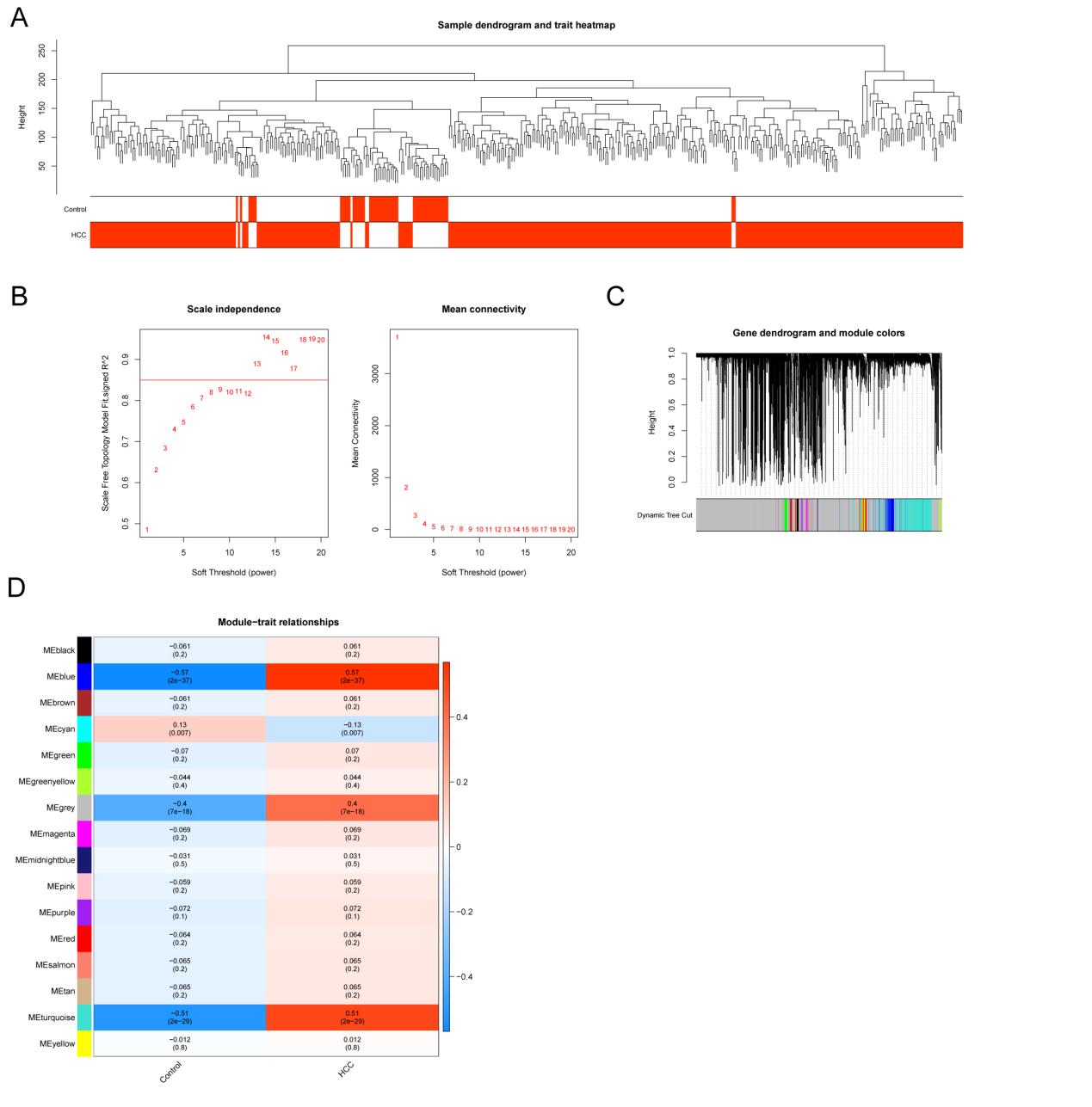
**

**Figure S1 Identification of key module genes in WGCNA**

(A) Sample clustering diagram. (B) Selection of soft threshold β. (C) Module clustering diagram. (D) Heat map of the relationship between gene modules and traits using HCC as the phenotype.
