# Supplementary material for: KIF18A induces the EMT process of hepatoma cells through the 5-LOX-dependent arachidonic acid pathway
Source: PLoS One. 2025 Oct 13;20(10):e0333385. doi: 10.1371/journal.pone.0333385 (PMC12517525; doi:10.1371/journal.pone.0333385)
Supplement: S1 Table — (DOCX) [file pone.0333385.s002.docx]

**Table S1 ELISA kit information**

| Gene name | Manufacturer | Article number | Species reactivity |
| --- | --- | --- | --- |
| 5-HETE | ELK Biotechnology | ELK8155 | Humans |
| 12-HETE | YaJi Biological | YS01886B | Humans |
| 15-HETE | ELK Biotechnology | ELK8351 | Humans |
| LTB4 | Elabscience | E-EL-0061 | Humans |
| LTC4 | RUIXIN BIOTECH | RX106172H | Humans |
| LTD4 | ELK Biotechnology | ELK9178 | Humans |
| AA | Elabscience | E-EL-0051 | Humans |
