# Supplementary material for: KIF18A induces the EMT process of hepatoma cells through the 5-LOX-dependent arachidonic acid pathway
Source: PLoS One. 2025 Oct 13;20(10):e0333385. doi: 10.1371/journal.pone.0333385 (PMC12517525; doi:10.1371/journal.pone.0333385)
Supplement: S2 Table — (DOCX) [file pone.0333385.s003.docx]

**Table S2 Antibody information used in this study**

| Gene name | Manufacturer | Article number | Dilution ratio |
| --- | --- | --- | --- |
| KIF18A | Abcam | ab72417 | 1/2000 |
| 5-LOX | affinity | AF4699 | 1/1000 |
| E-cadherin | bioss | bs-1519R | 1/1000 |
| N-cadherin | ABclonal | A1908 | 1/1000 |
| Snail1 | affinity | AF6032 | 1/1000 |
| Vimentin | abclonal | A19607 | 1/2000 |
| GAPDH | abclonal | A19056 | 1/50000 |
| Sheep anti-rabbit fluorescent secondary antibody -488 | ZSBio | ZF-0511 | 1/200 |
| WB Second Anti | Bioss | K008 | 1/10000 |
