# Supplementary material for: KIF18A induces the EMT process of hepatoma cells through the 5-LOX-dependent arachidonic acid pathway
Source: PLoS One. 2025 Oct 13;20(10):e0333385. doi: 10.1371/journal.pone.0333385 (PMC12517525; doi:10.1371/journal.pone.0333385)
Supplement: S3 Table — (DOCX) [file pone.0333385.s004.docx]

**Table S3 RT-qPCR primer information and sequence information related to knockdown and overexpression**

| Gene | Forward Primer (5′–3′) | Reverse Primer (5′–3′) |
| --- | --- | --- |
| KIF18A | CACTTGCTGTCCGGGAAGAT | AAGTCCATGAACGACCACCC |
| GAPDH | GAGAAGGCTGGGGCTCATTT | AGTGATGGCATGGACTGTGG |
| Gene | SS Sequence | AS Sequence |
| SiNC | UCUUCAAGGGGUCUACAUGGC | GGUUUACAUGUUCCAAUAUGA |
| SiKIF18A-1 | UUGAAUACUUCUCUUCAUGUG | CAUGAAGAGAAGUAUUCAAGU |
| SiKIF18A-2 | AAAUACUUGAAUACUUCUCUU | GAGAAGUAUUCAAGUAUUUAU |
| SiKIF18A-3 | UGUAUAAAUACUUGAAUACUU | GUAUUCAAGUAUUUAUACAGA |
| Gene | Primer sequence (5 '-3') | |
| pcDNA3.1-KIF18A-F | GGTACCGAGCTCGGATCCATGTCTGTCACTGAGGAAGAC | |
| pcDNA3.1-KIF18A-R | GGATATCTGCAGAATTCTTATCTTAGATTTCCTTTTG | |
