# Supplementary material for: KIF18A induces the EMT process of hepatoma cells through the 5-LOX-dependent arachidonic acid pathway
Source: PLoS One. 2025 Oct 13;20(10):e0333385. doi: 10.1371/journal.pone.0333385 (PMC12517525; doi:10.1371/journal.pone.0333385)
Supplement: S1 Raw Images — (PDF) [file pone.0333385.s005.pdf]

Figure3-D

KIF18A

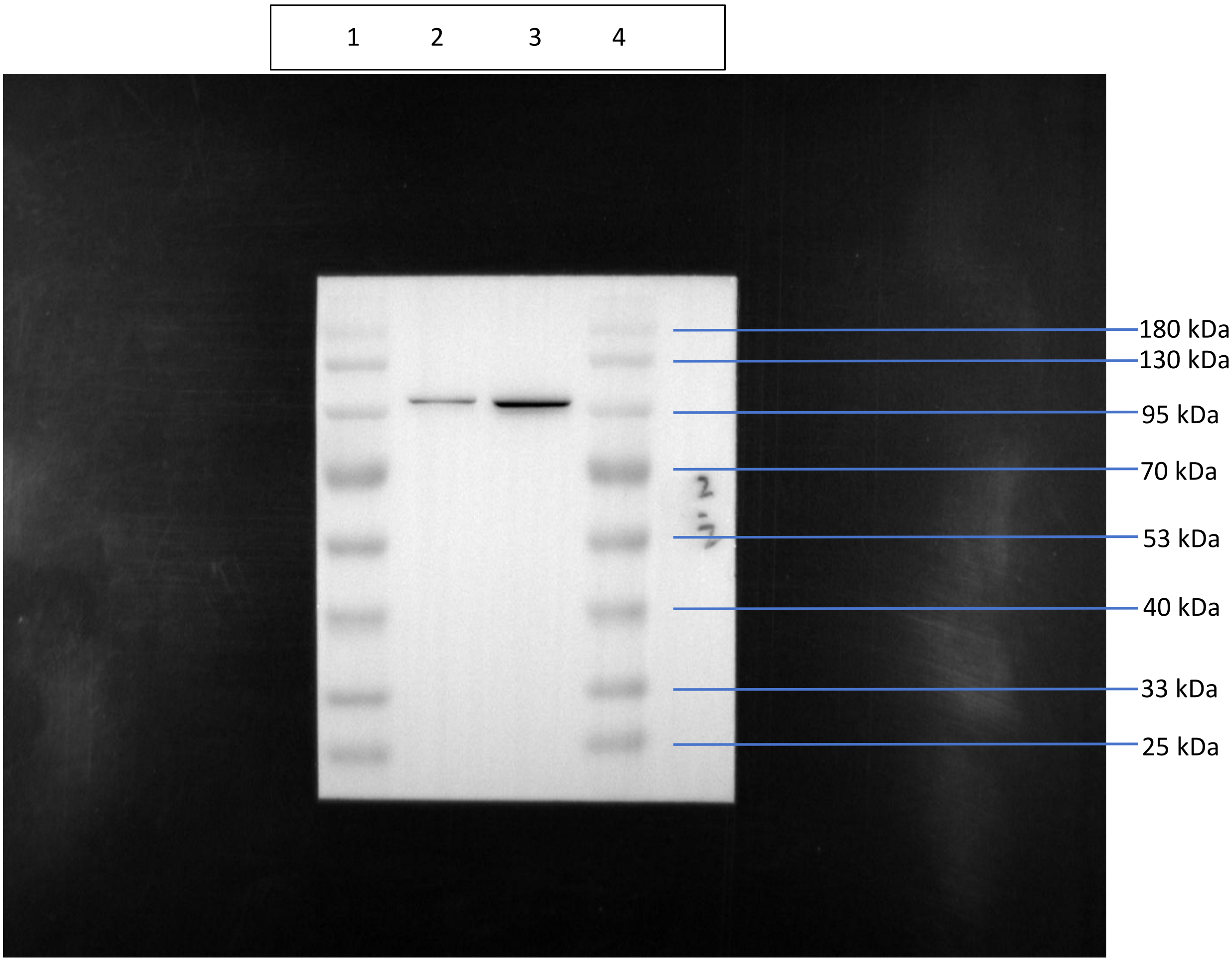

Lane1：Protein marker

Lane2：Vector

Lane3：oe-KIF18A

Lane4：Protein marker

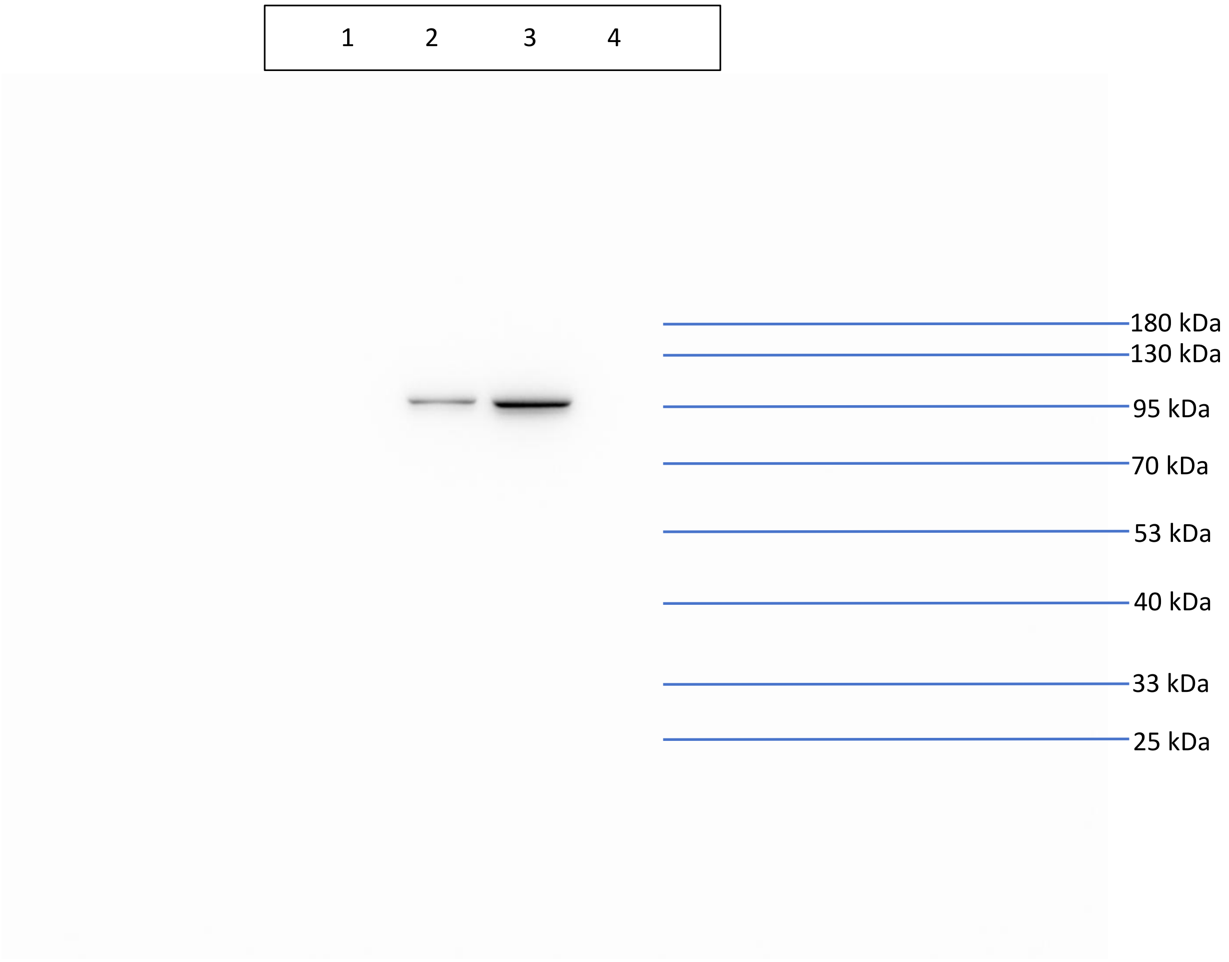

Lane1：Protein marker

Lane2：Vector

Lane3：oe-KIF18A

Lane4：Protein marker

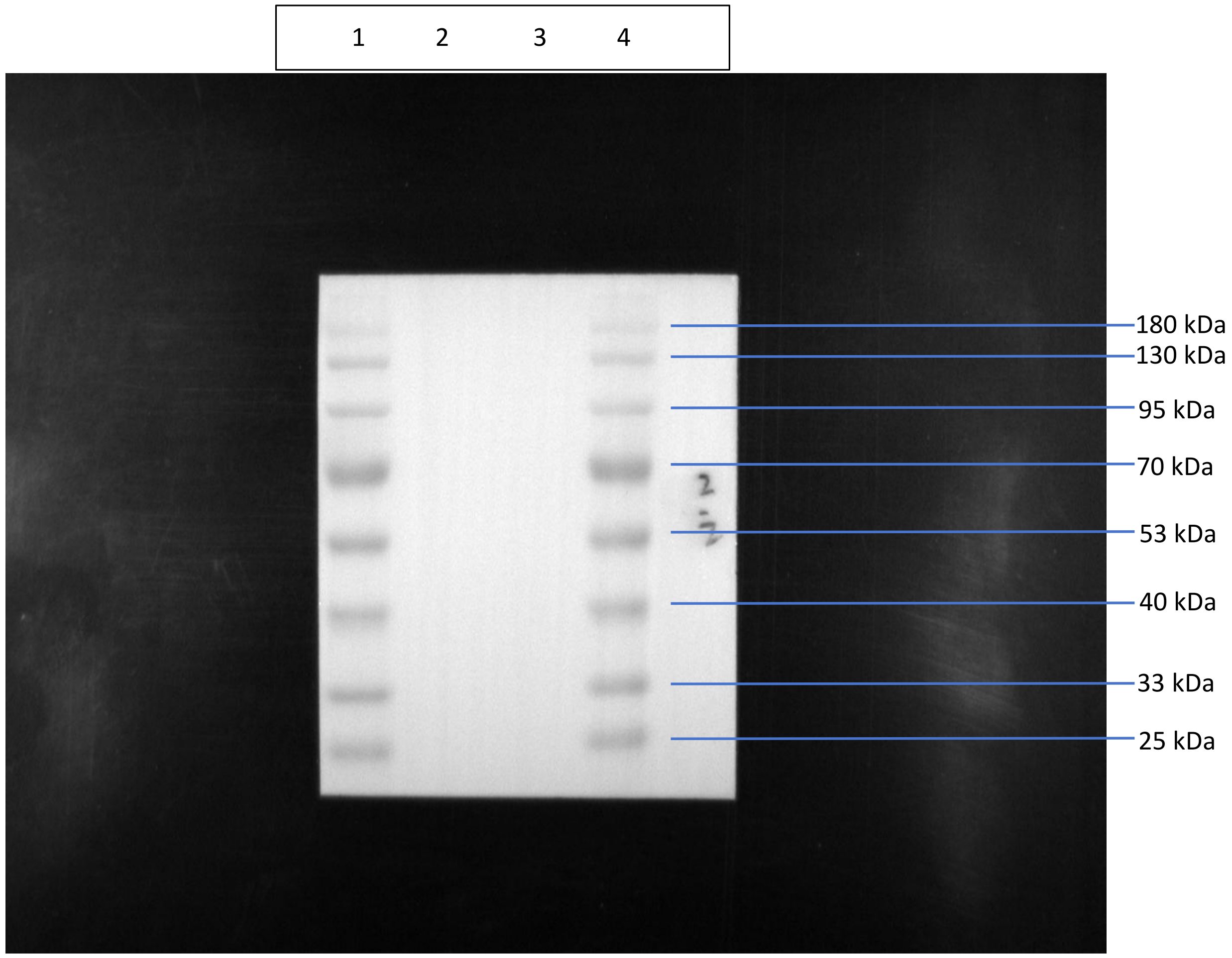

Lane1：Protein marker

Lane2：Vector

Lane3：oe-KIF18A

Lane4：Protein marker

GAPDH

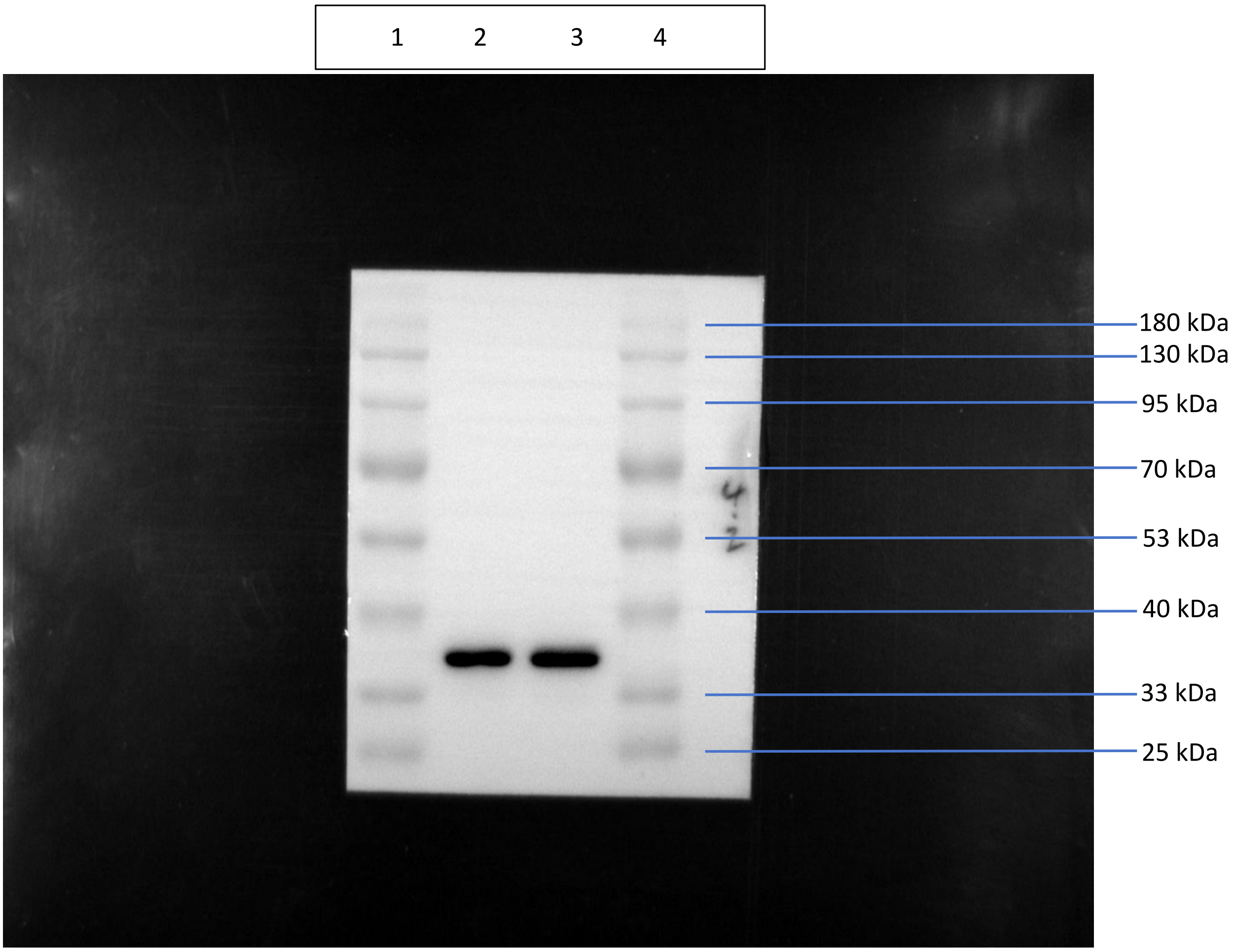

Lane1：Protein marker

Lane2：Vector

Lane3：oe-KIF18A

Lane4：Protein marker

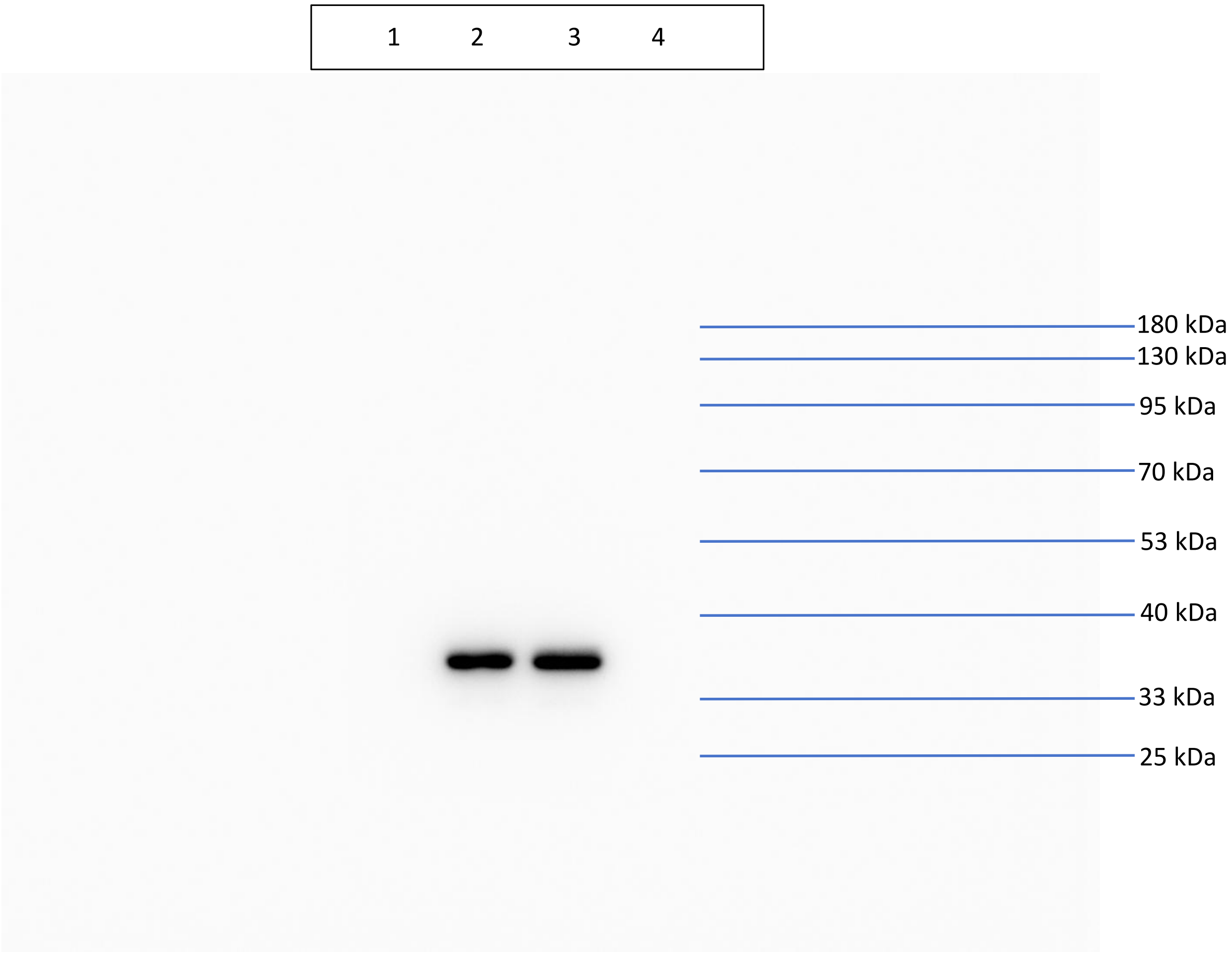

Lane1：Protein marker

Lane2：Vector

Lane3：oe-KIF18A

Lane4：Protein marker

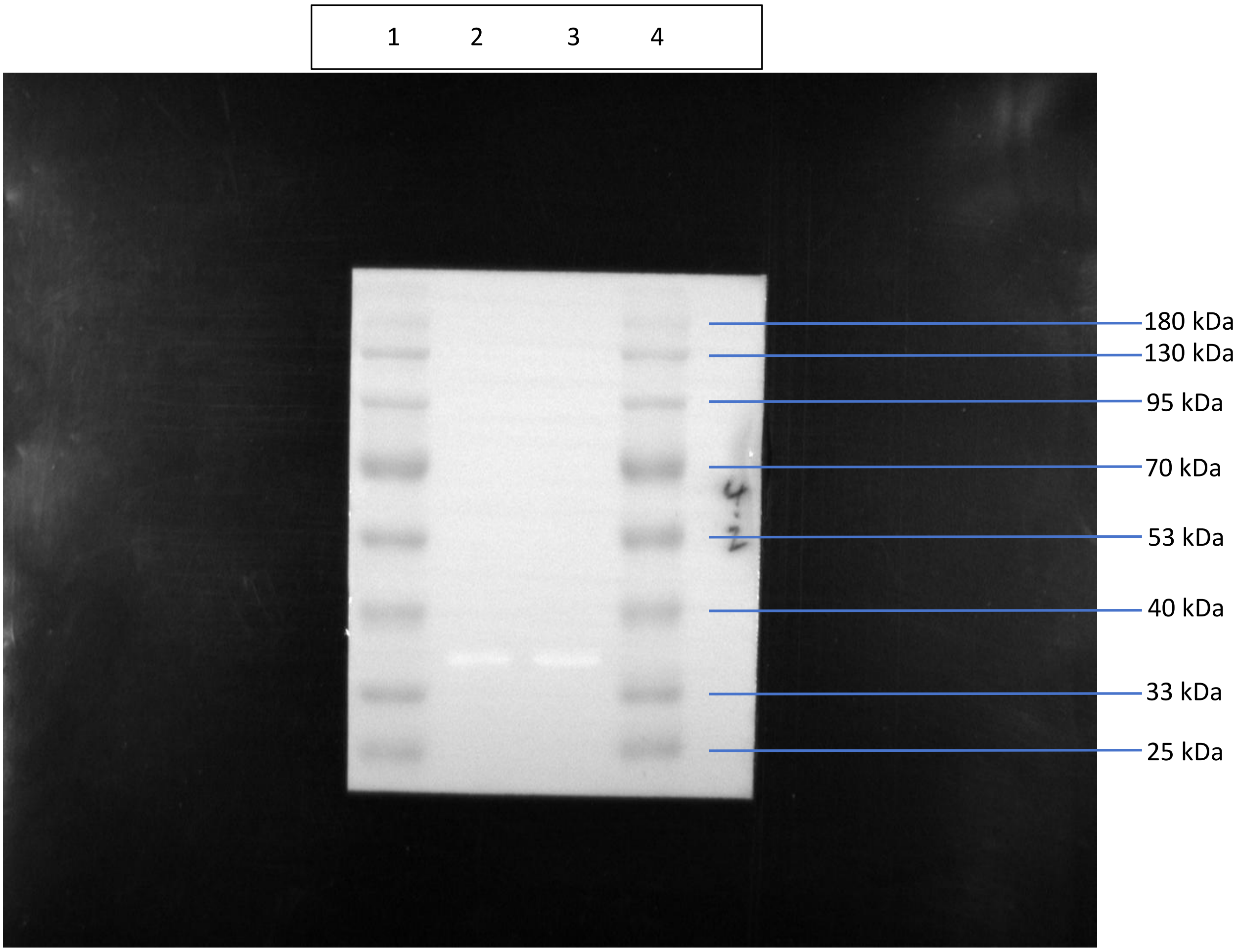

Lane1： Protein marker

Lane2： Vector

Lane3： oe-KIF18A

Lane4： Protein marker

Figure3-G

KIF18A

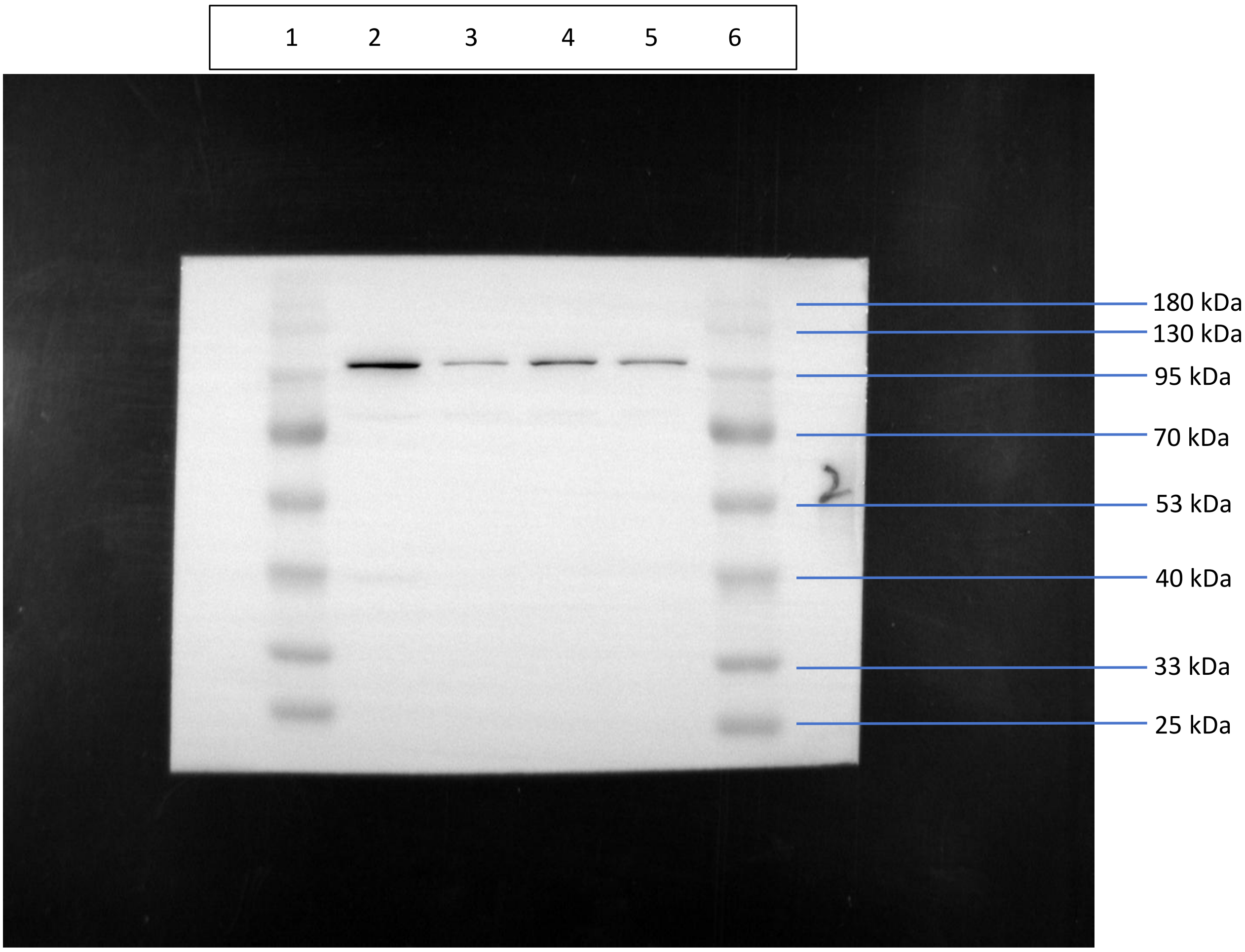

Lane1：Protein marker

Lane2：si-NC

Lane3：si-KIF18A-1

Lane4：si-KIF18A-2

Lane5：si-KIF18A-3

Lane6：Protein marker

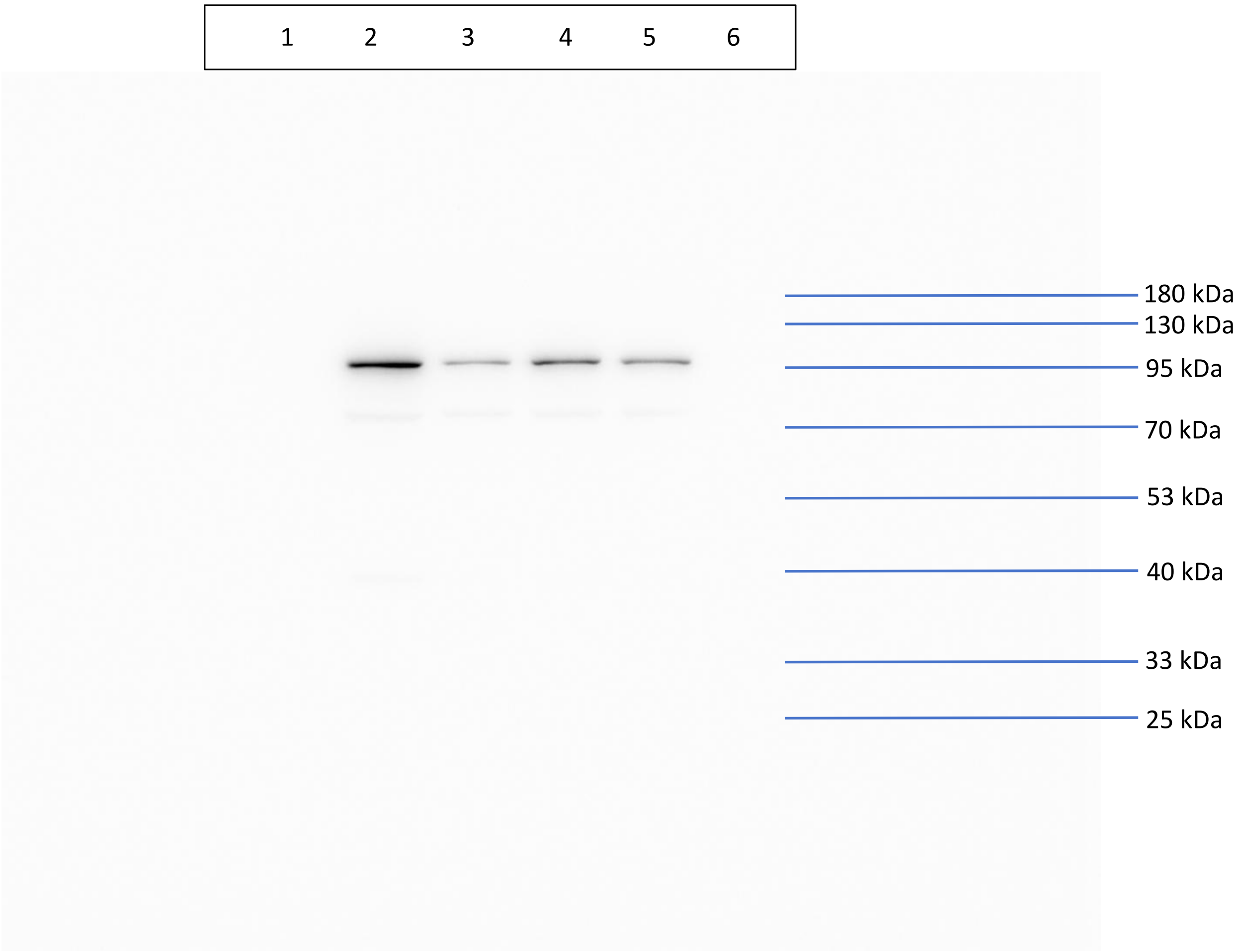

Lane1：Protein marker

Lane2：si-NC

Lane3：si-KIF18A-1

Lane4：si-KIF18A-2

Lane5：si-KIF18A-3

Lane6：Protein marker

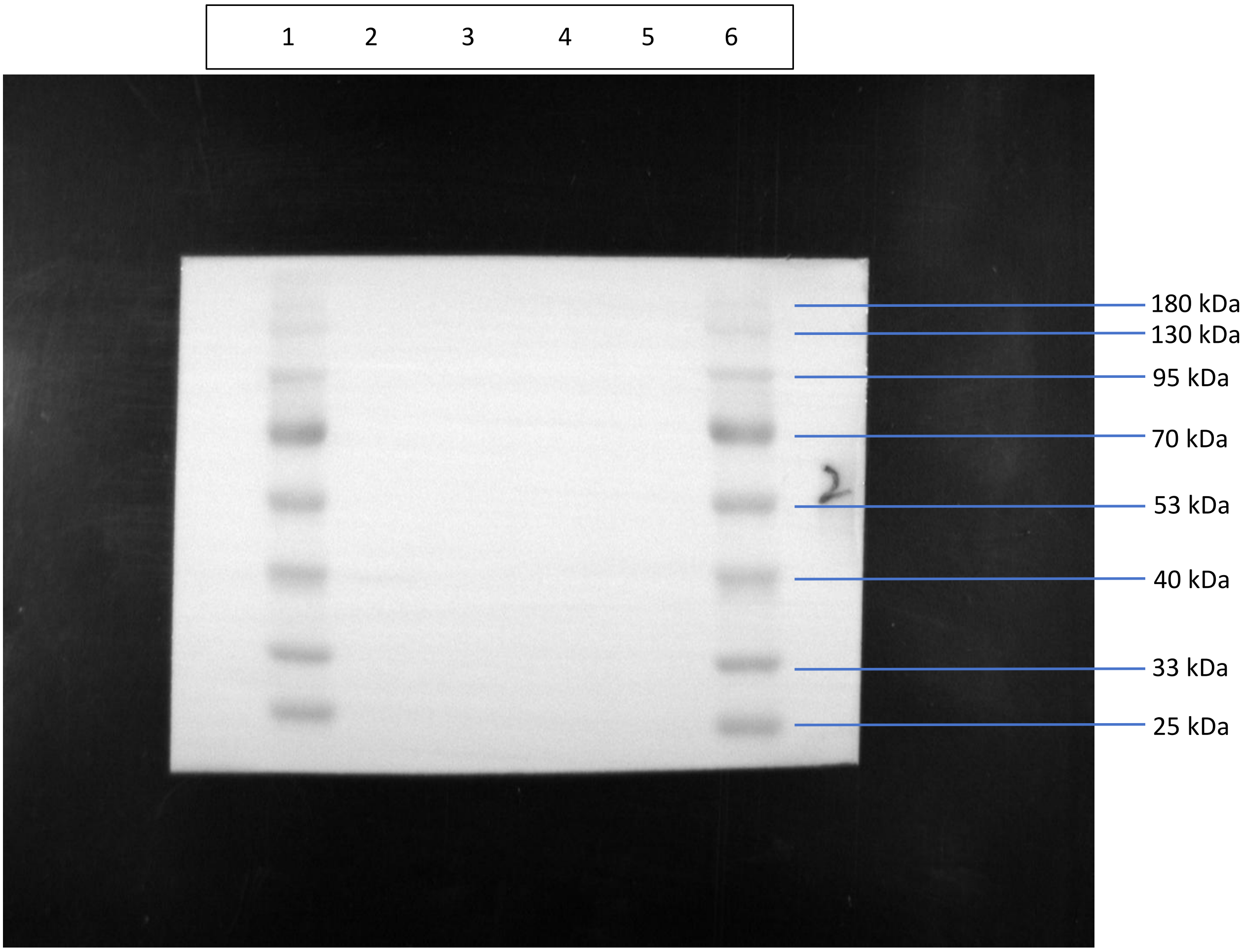

Lane1：Protein marker

Lane2：si-NC

Lane3：si-KIF18A-1

Lane4：si-KIF18A-2

Lane5：si-KIF18A-3

Lane6：Protein marker

GAPDH

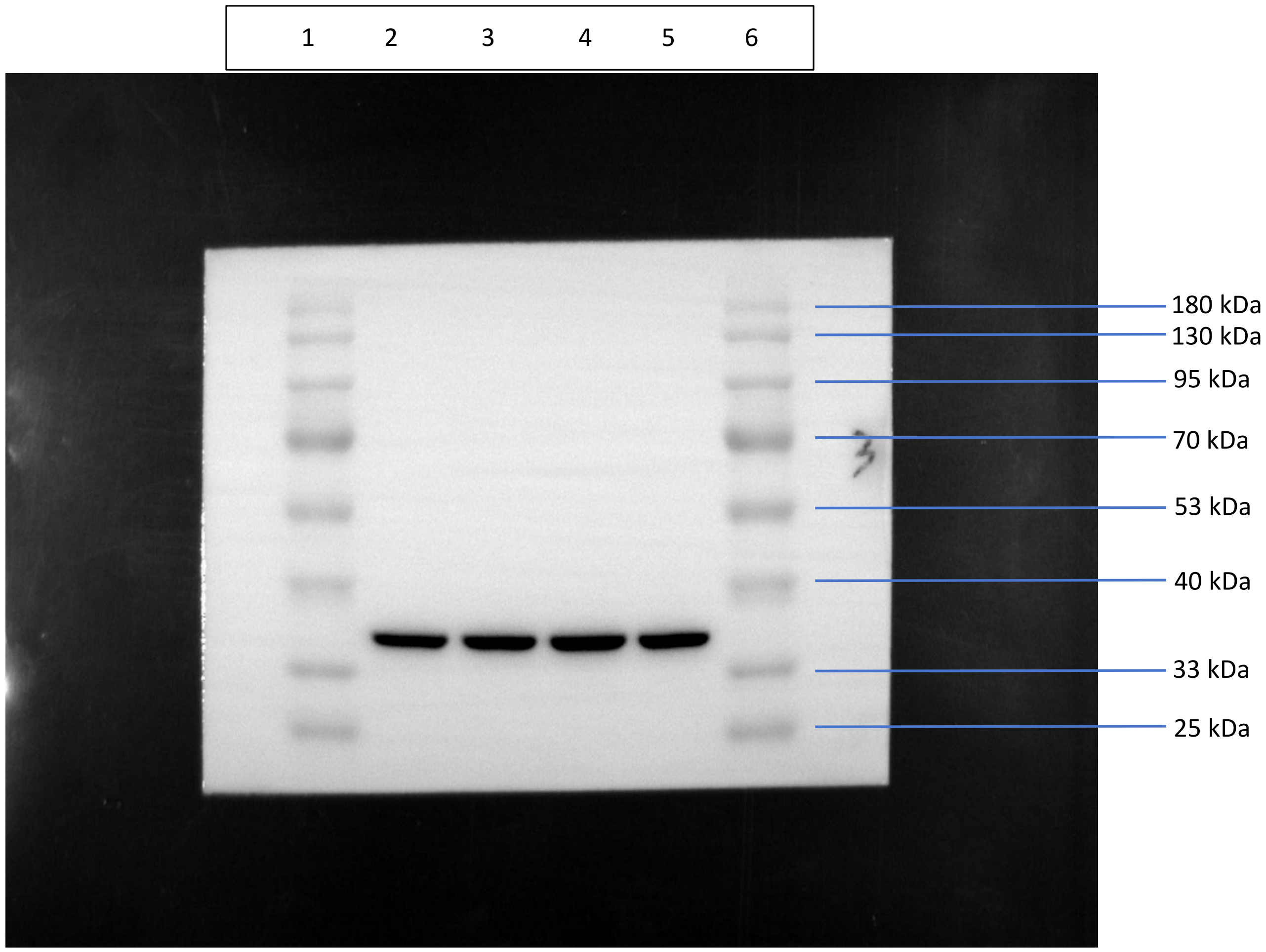

Lane1：Protein marker

Lane2：si-NC

Lane3：si-KIF18A-1

Lane4：si-KIF18A-2

Lane5：si-KIF18A-3

Lane6：Protein marker

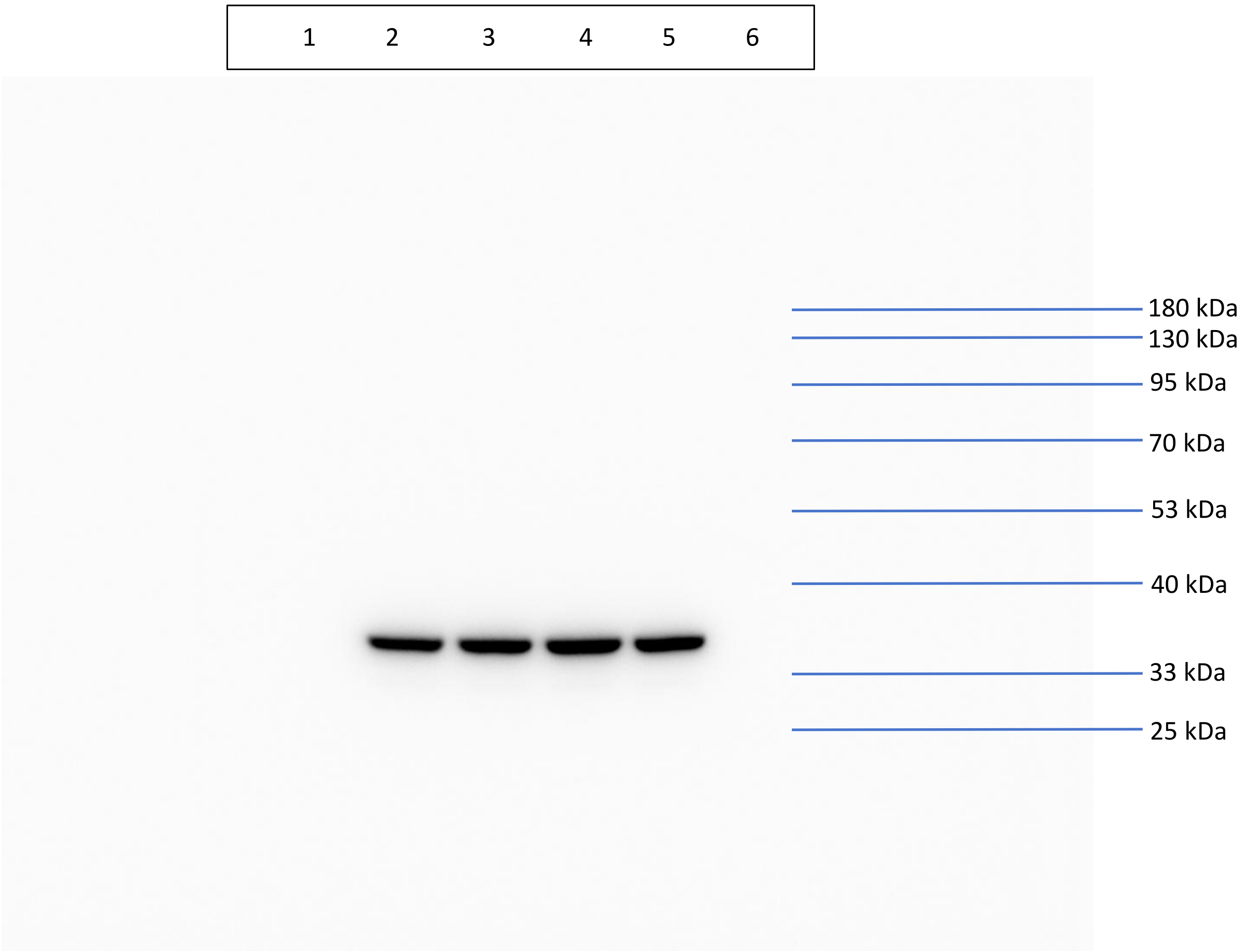

Lane1：Protein marker

Lane2：si-NC

Lane3：si-KIF18A-1

Lane4：si-KIF18A-2

Lane5：si-KIF18A-3

Lane6：Protein marker

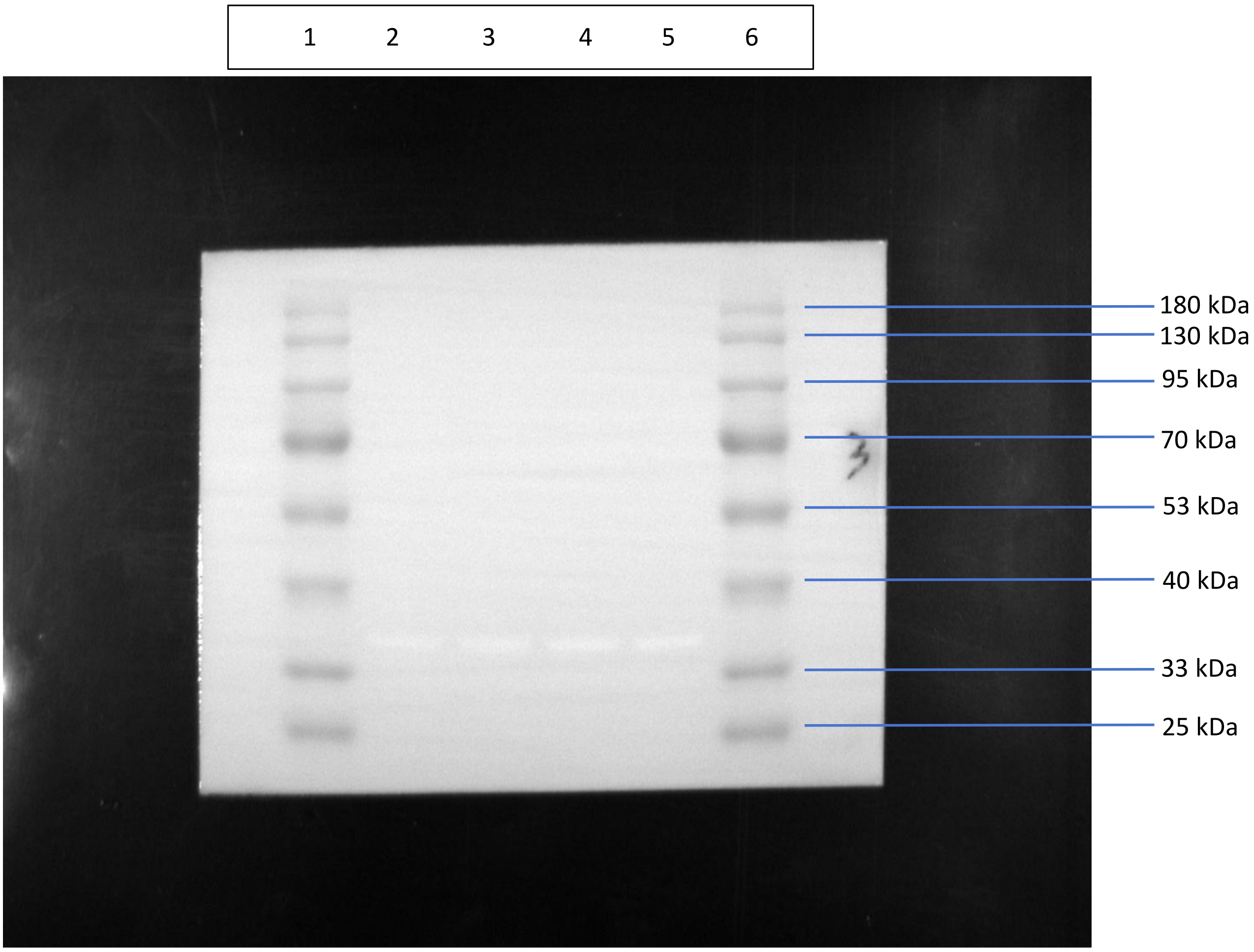

Lane1：Protein marker

Lane2：si-NC

Lane3：si-KIF18A-1

Lane4：si-KIF18A-2

Lane5：si-KIF18A-3

Lane6：Protein marker

Figure5-E

E-Cadherin

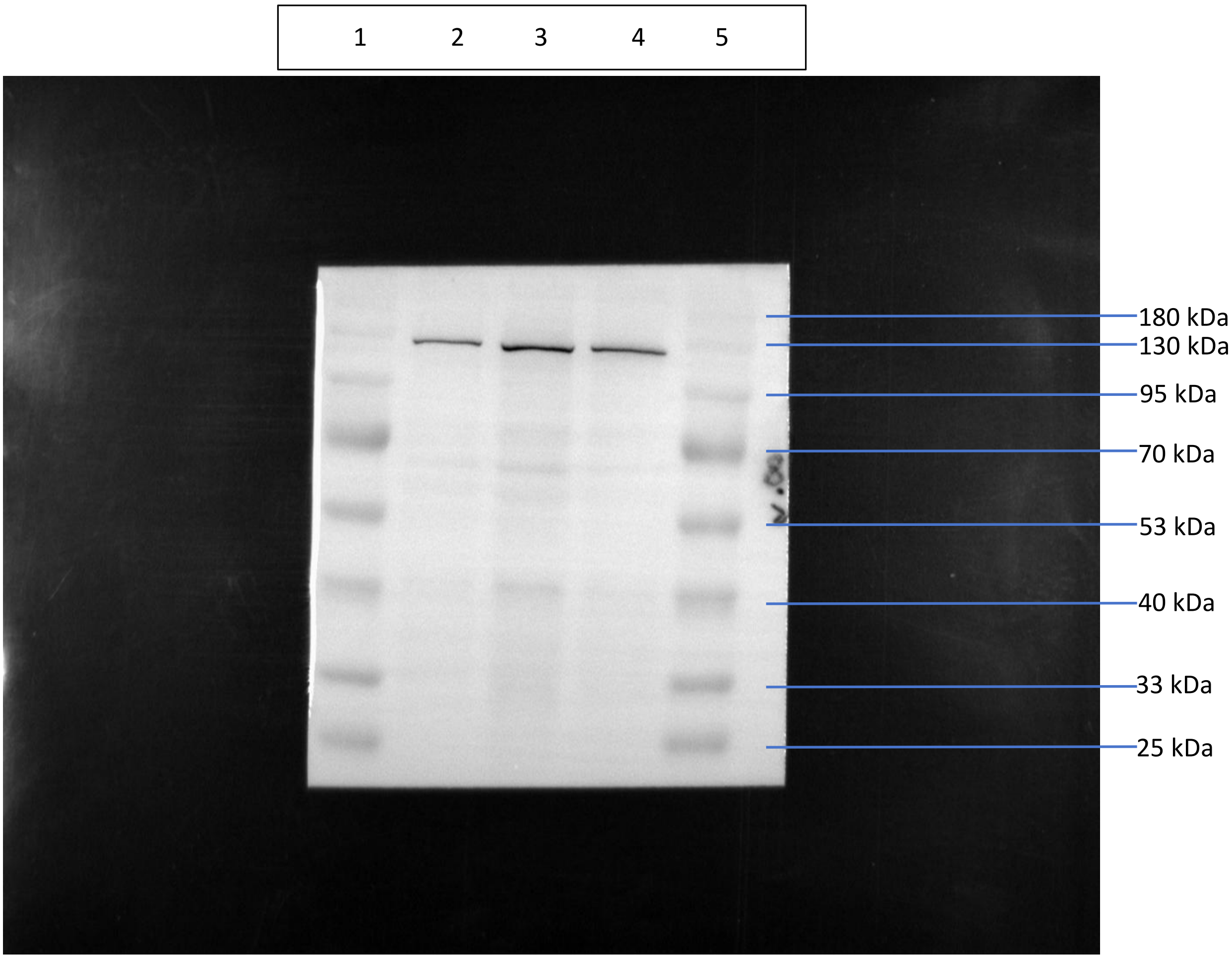

Lane1：Protein marker

Lane2：Vector

Lane3：oe-KIF18A

Lane4：oe-KIF18A+U73122

Lane5：Protein marker

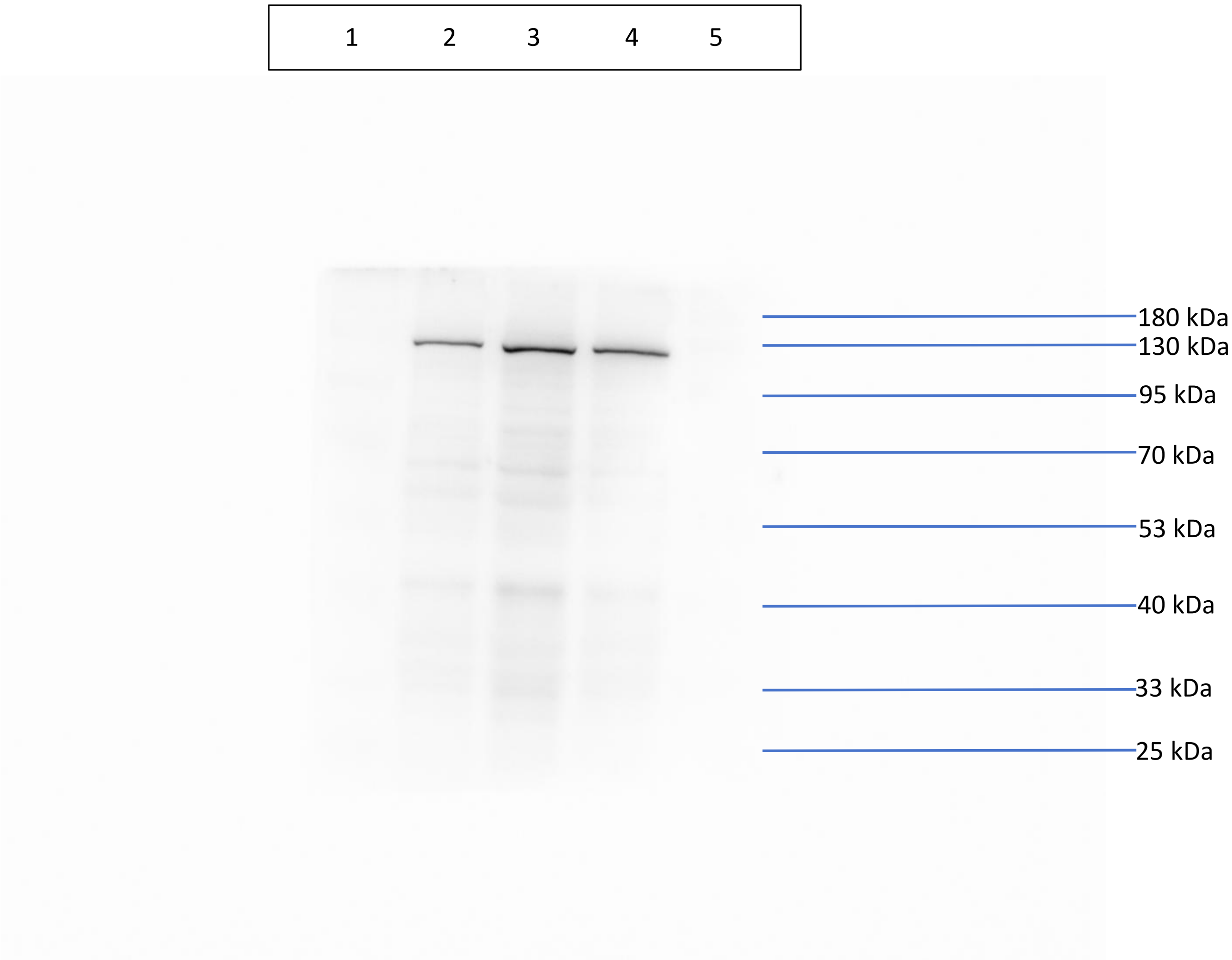

Lane1：Protein marker

Lane2：Vector

Lane3：oe-KIF18A

Lane4：oe-KIF18A+U73122

Lane5：Protein marker

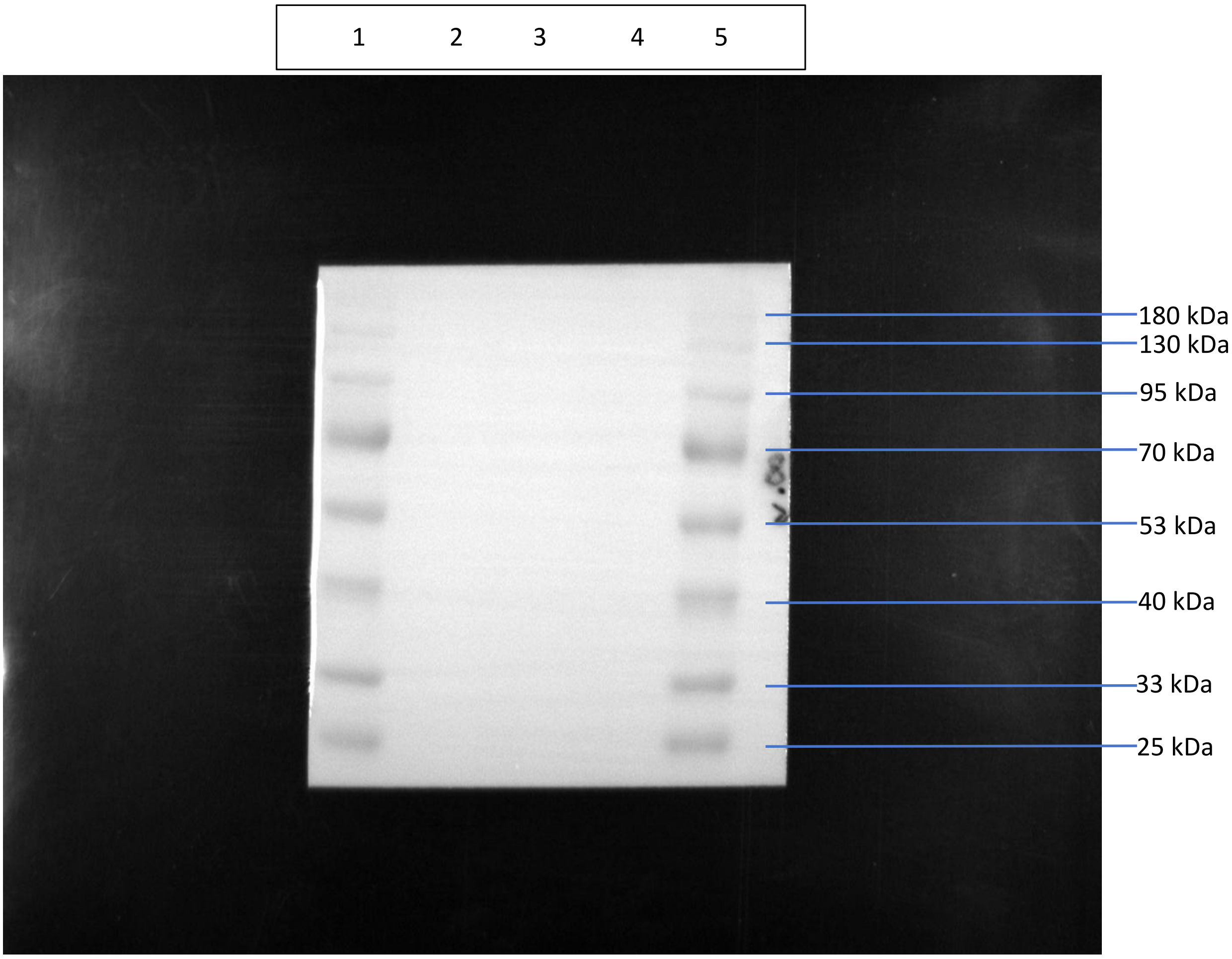

Lane1：Protein marker

Lane2：Vector

Lane3：oe-KIF18A

Lane4：oe-KIF18A+U73122

Lane5：Protein marker

N-Cadherin

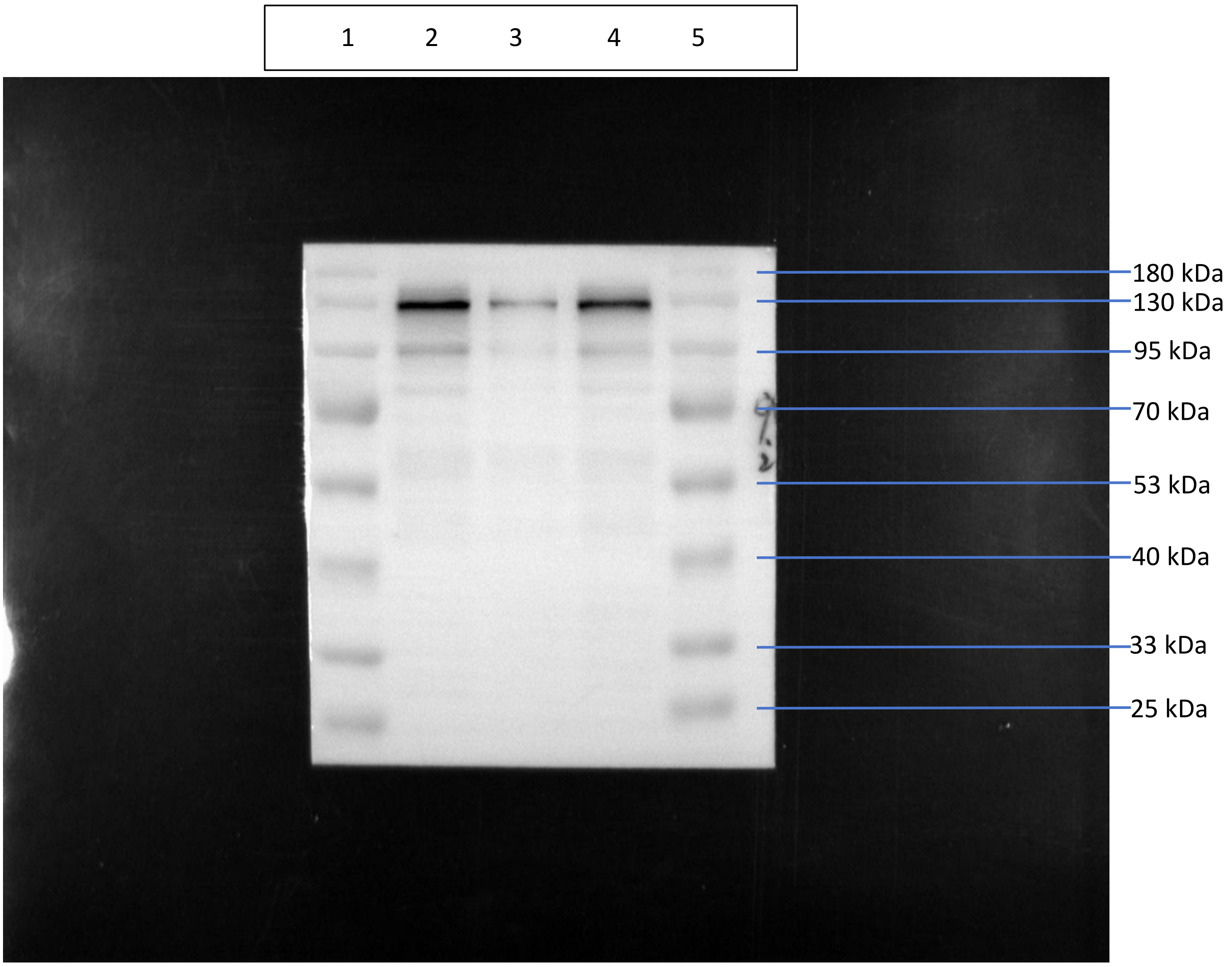

Lane1：Protein marker

Lane2：Vector

Lane3：oe-KIF18A

Lane4：oe-KIF18A+U73122

Lane5：Protein marker

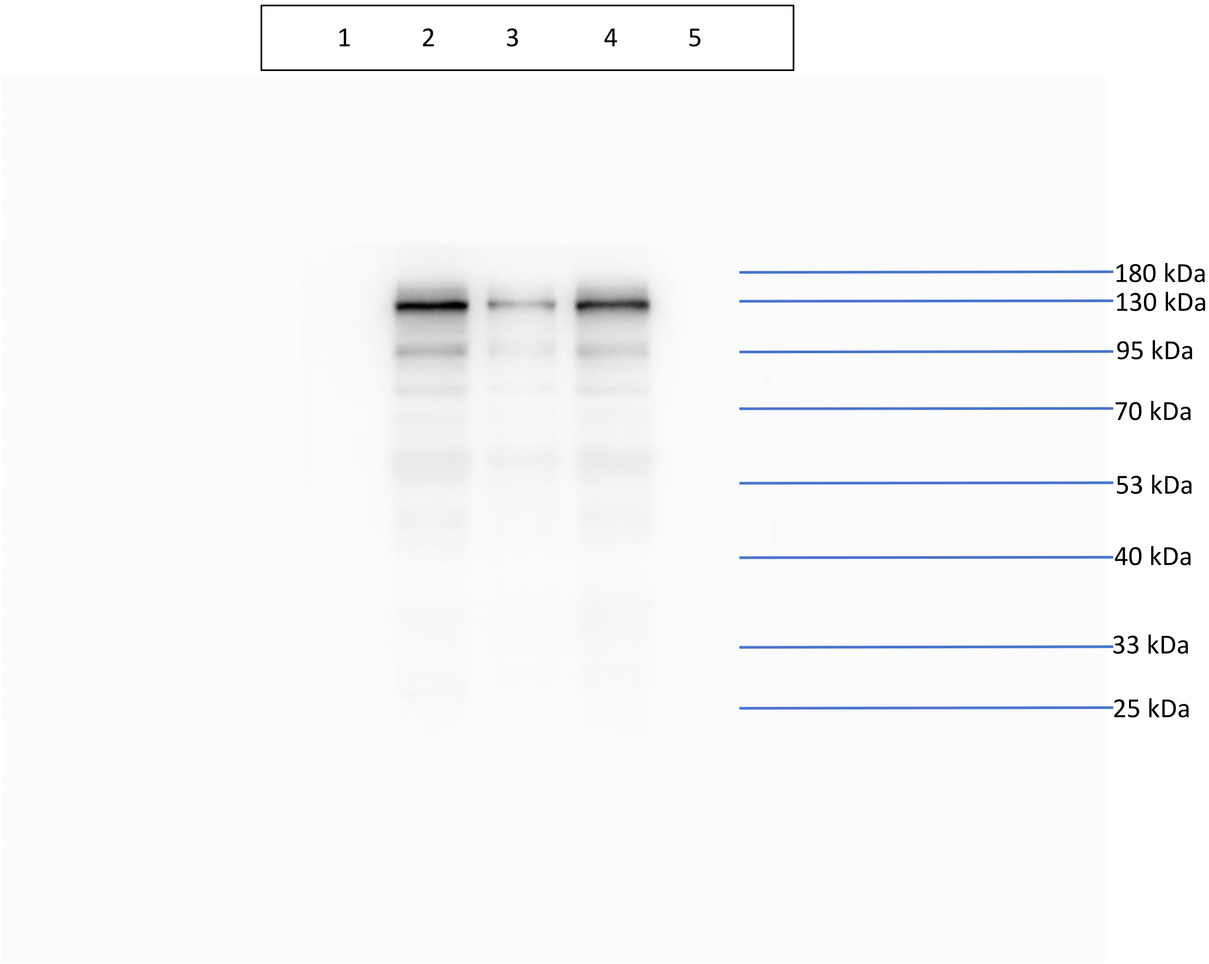

Lane1：Protein marker

Lane2：Vector

Lane3：oe-KIF18A

Lane4：oe-KIF18A+U73122

Lane5：Protein marker

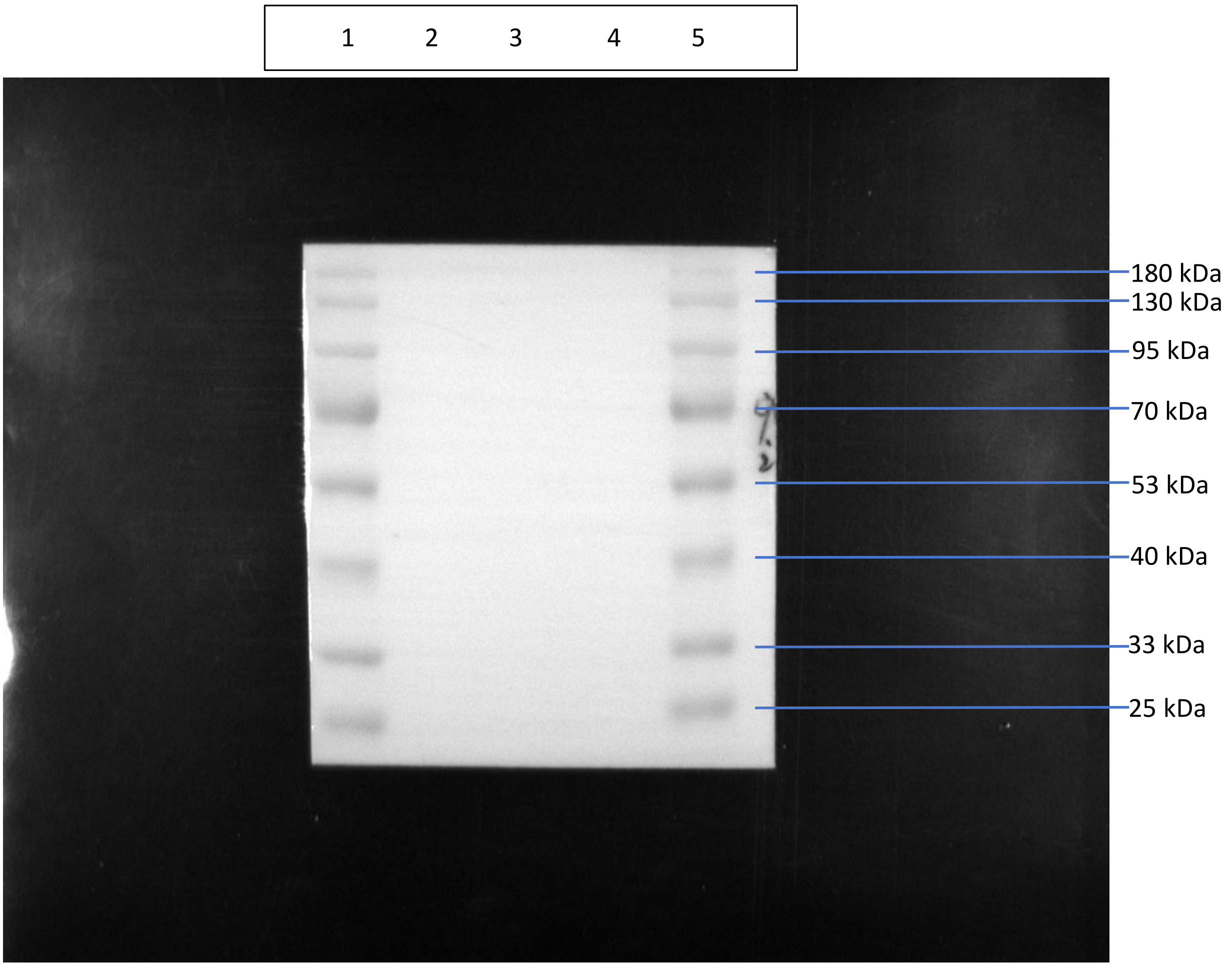

Lane1： Protein marker

Lane2： Vector

Lane3： oe-KIF18A

Lane4： oe-KIF18A+U73122

Lane5： Protein marker

Snail1

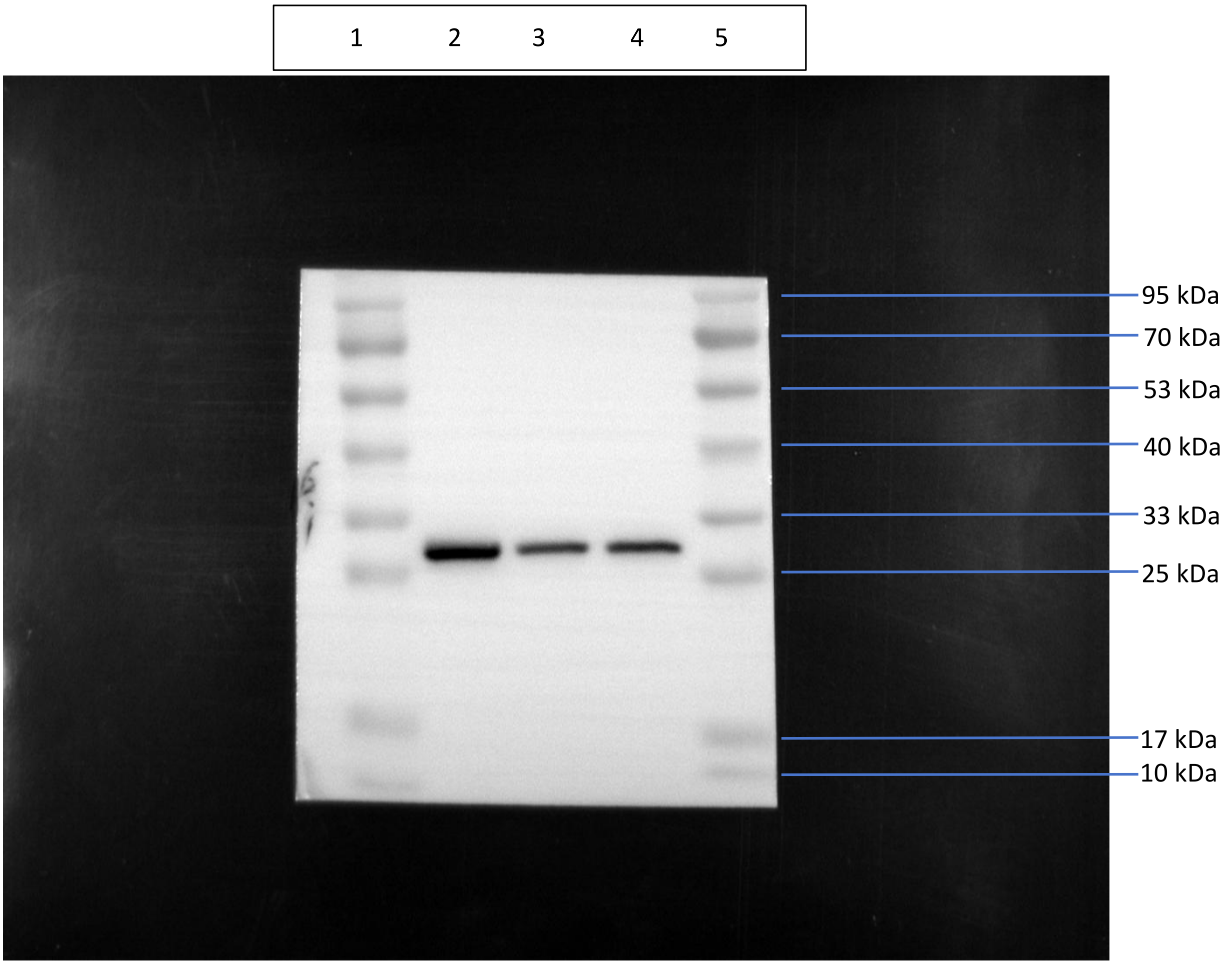

Lane1：Protein marker

Lane2：Vector

Lane3：oe-KIF18A

Lane4：oe-KIF18A+U73122

Lane5：Protein marker

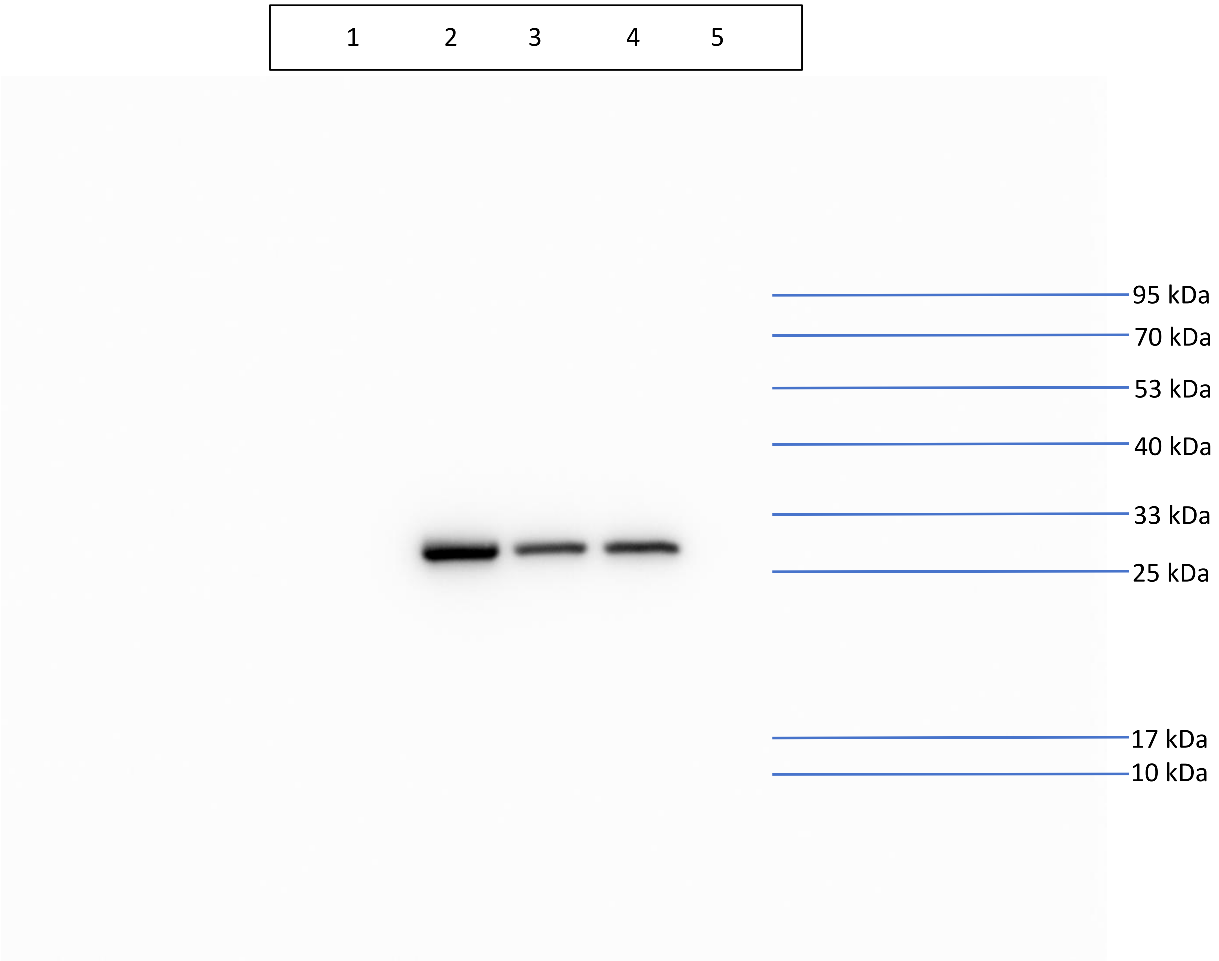

Lane1：Protein marker

Lane2：Vector

Lane3：oe-KIF18A

Lane4：oe-KIF18A+U73122

Lane5：Protein marker

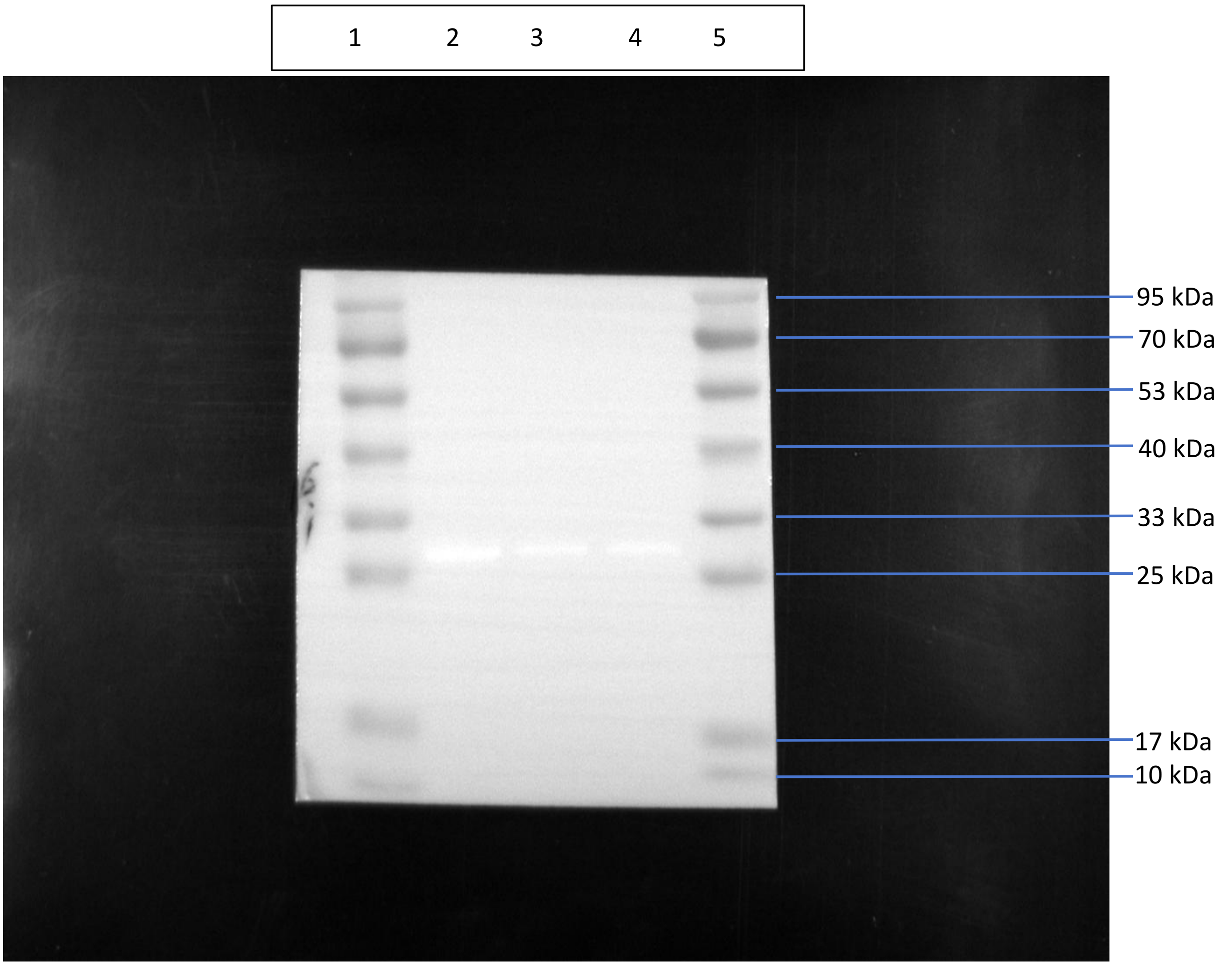

Lane1：Protein marker

Lane2：Vector

Lane3：oe-KIF18A

Lane4：oe-KIF18A+U73122

Lane5：Protein marker

Vimentin

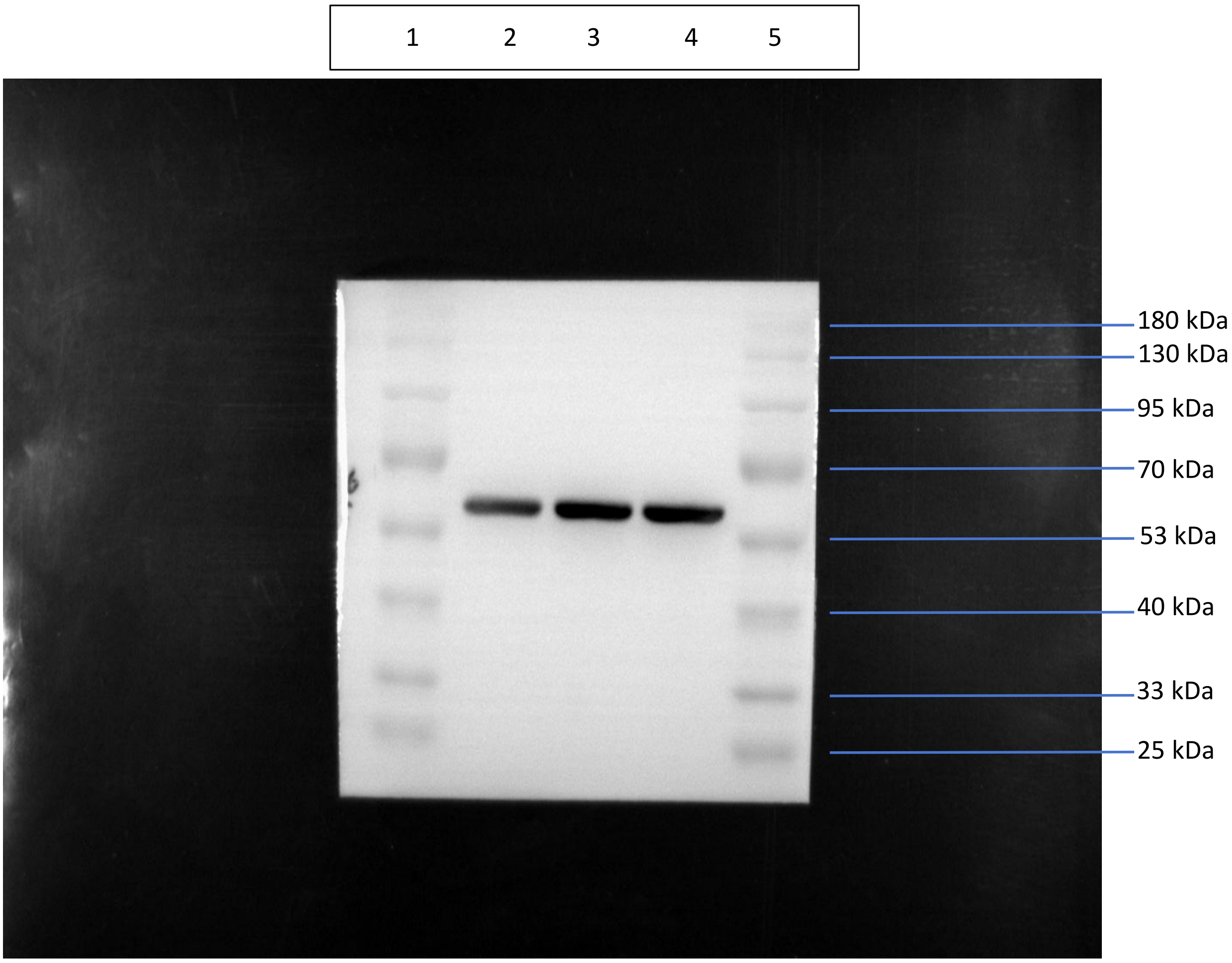

Lane1：Protein marker

Lane2：Vector

Lane3：oe-KIF18A

Lane4：oe-KIF18A+U73122

Lane5：Protein marker

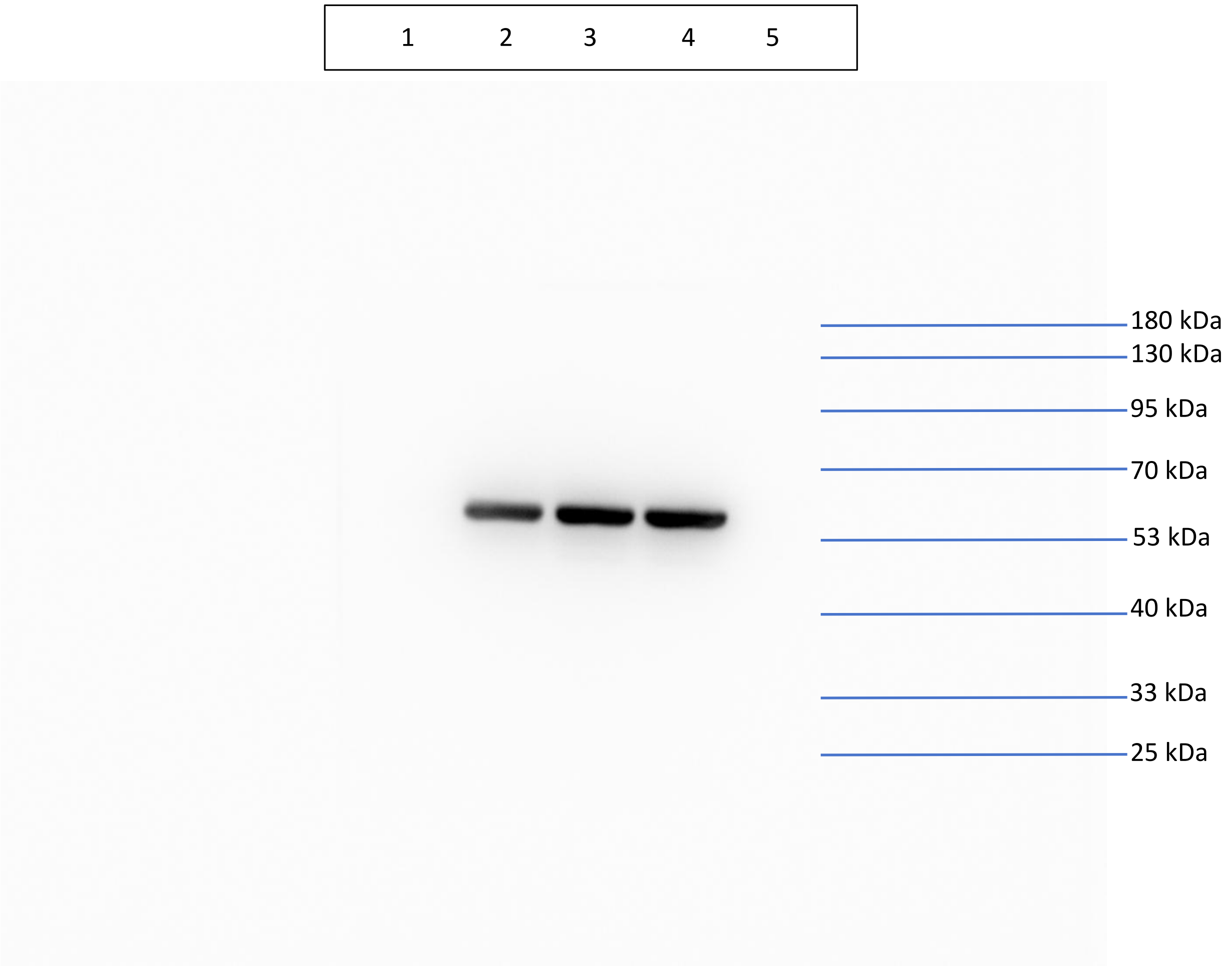

Lane1：Protein marker

Lane2：Vector

Lane3：oe-KIF18A

Lane4：oe-KIF18A+U73122

Lane5：Protein marker

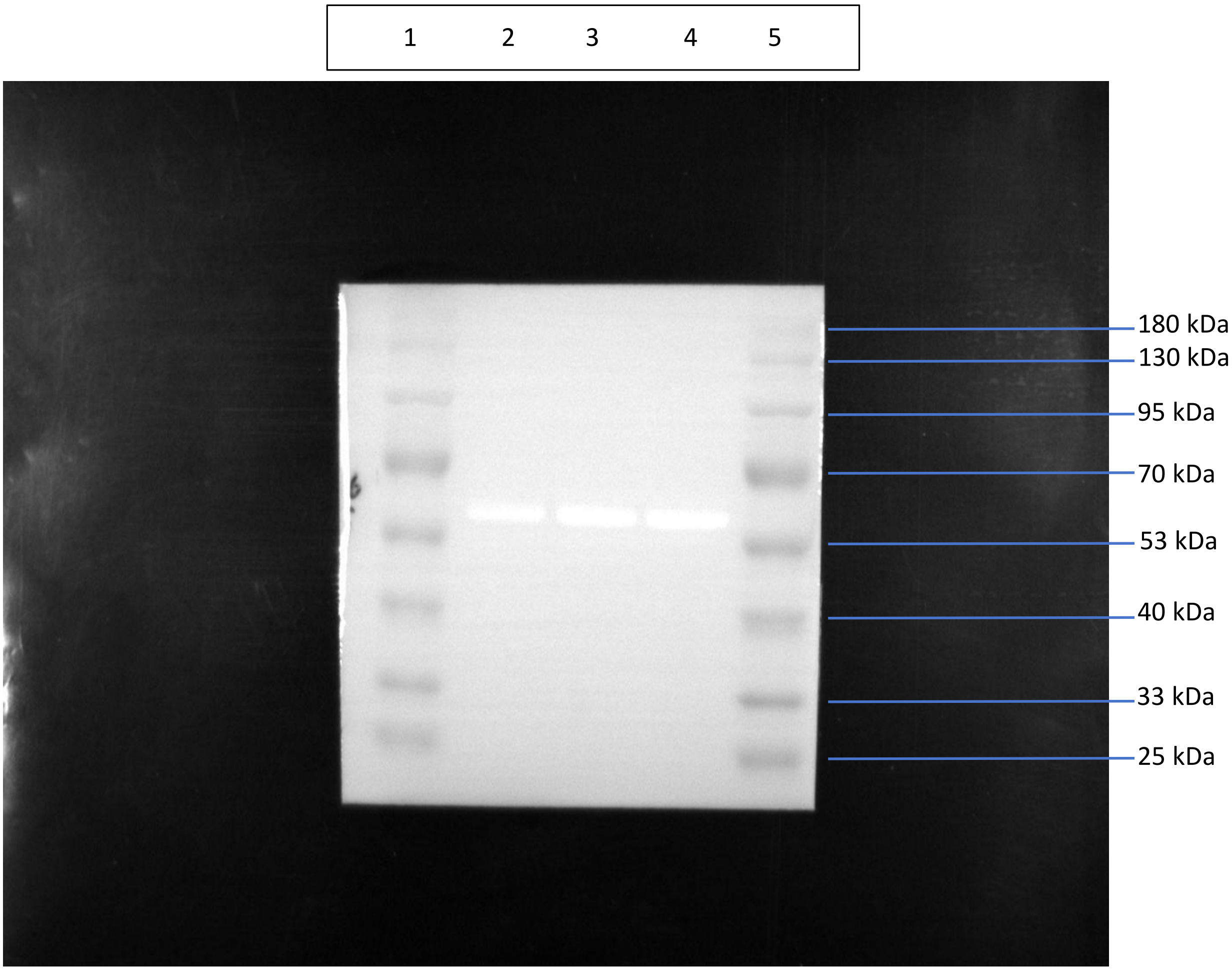

Lane1：Protein marker

Lane2：Vector

Lane3：oe-KIF18A

Lane4：oe-KIF18A+U73122

Lane5：Protein marker

GAPDH

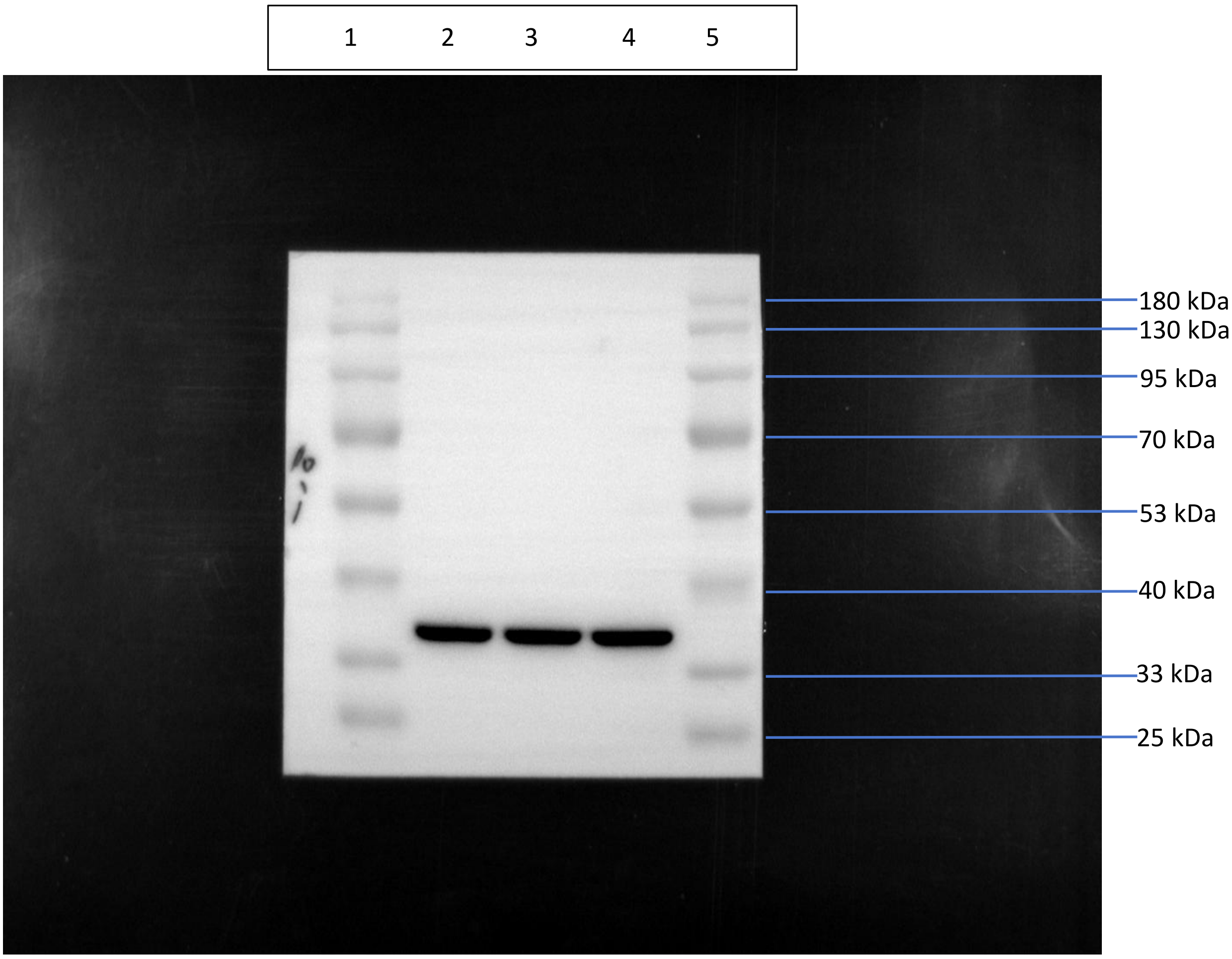

Lane1：Protein marker

Lane2：Vector

Lane3：oe-KIF18A

Lane4：oe-KIF18A+U73122

Lane5：Protein marker

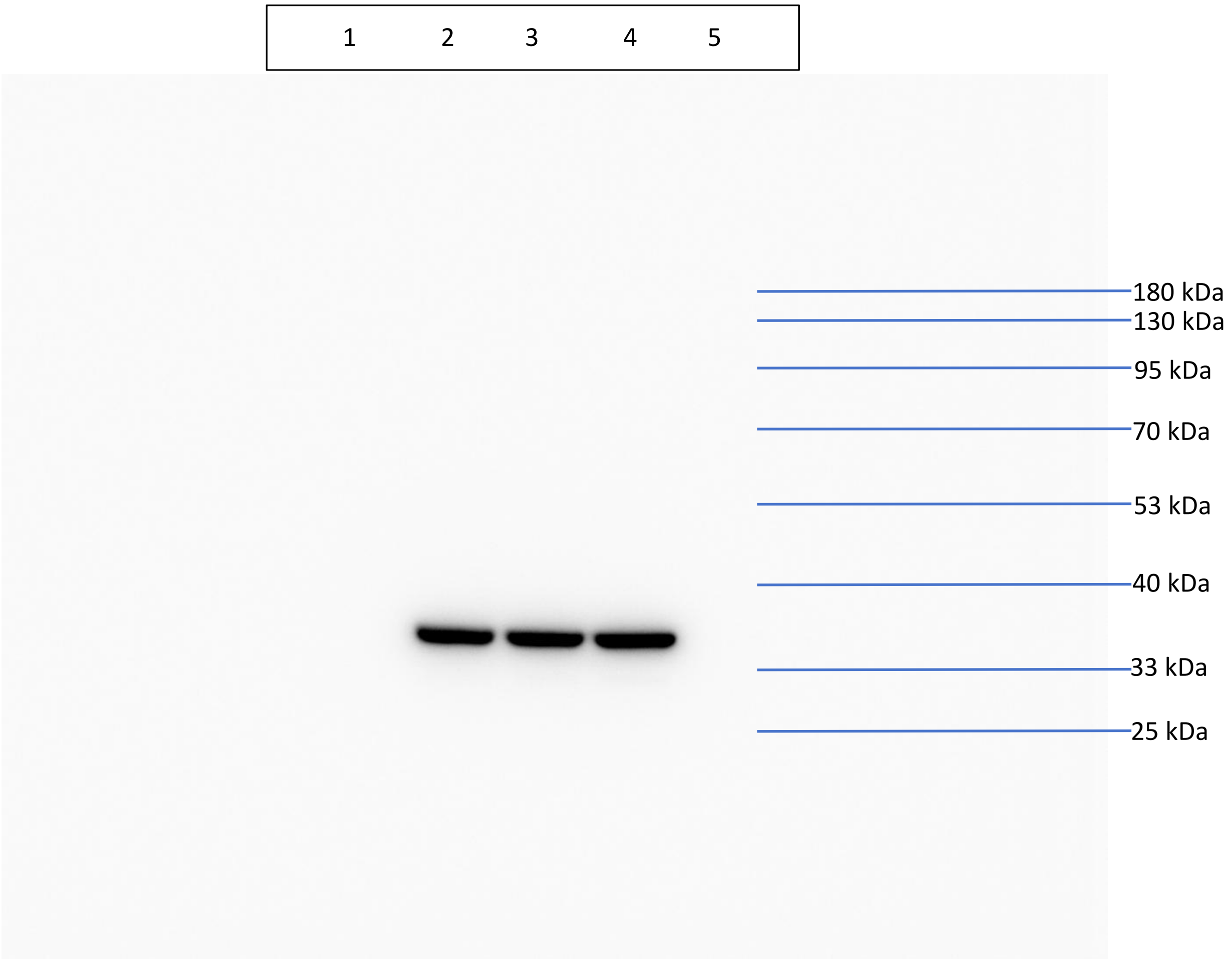

Lane1： Protein marker

Lane2： Vector

Lane3： oe-KIF18A

Lane4： oe-KIF18A+U73122

Lane5： Protein marker

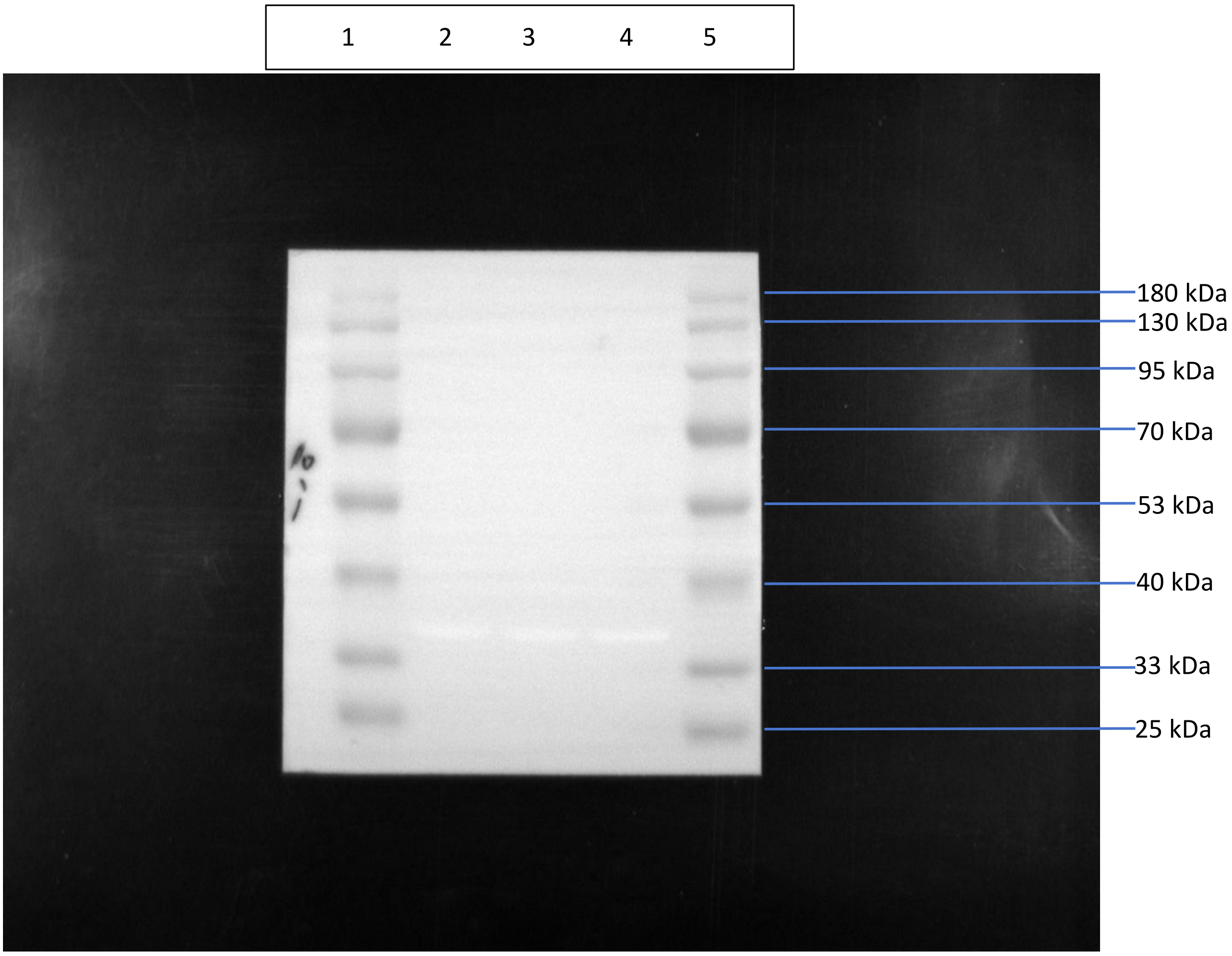

Lane1：Protein marker

Lane2：Vector

Lane3：oe-KIF18A

Lane4：oe-KIF18A+U73122

Lane5：Protein marker

Figure5-J

E-Cadherin

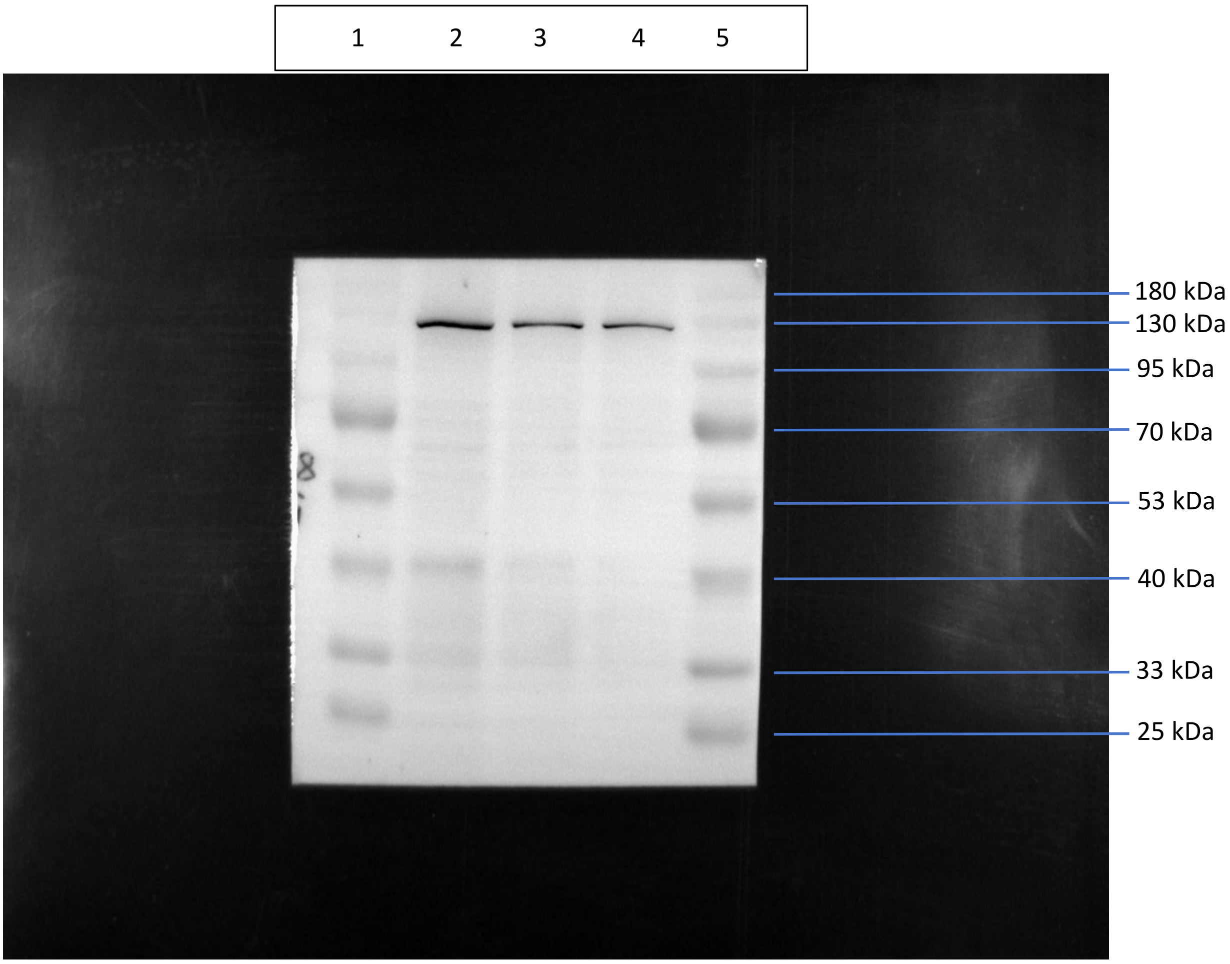

Lane1：Protein marker

Lane2：si-NC

Lane3：si-KIF18A-1

Lane4：si-KIF18A-2

Lane5：Protein marker

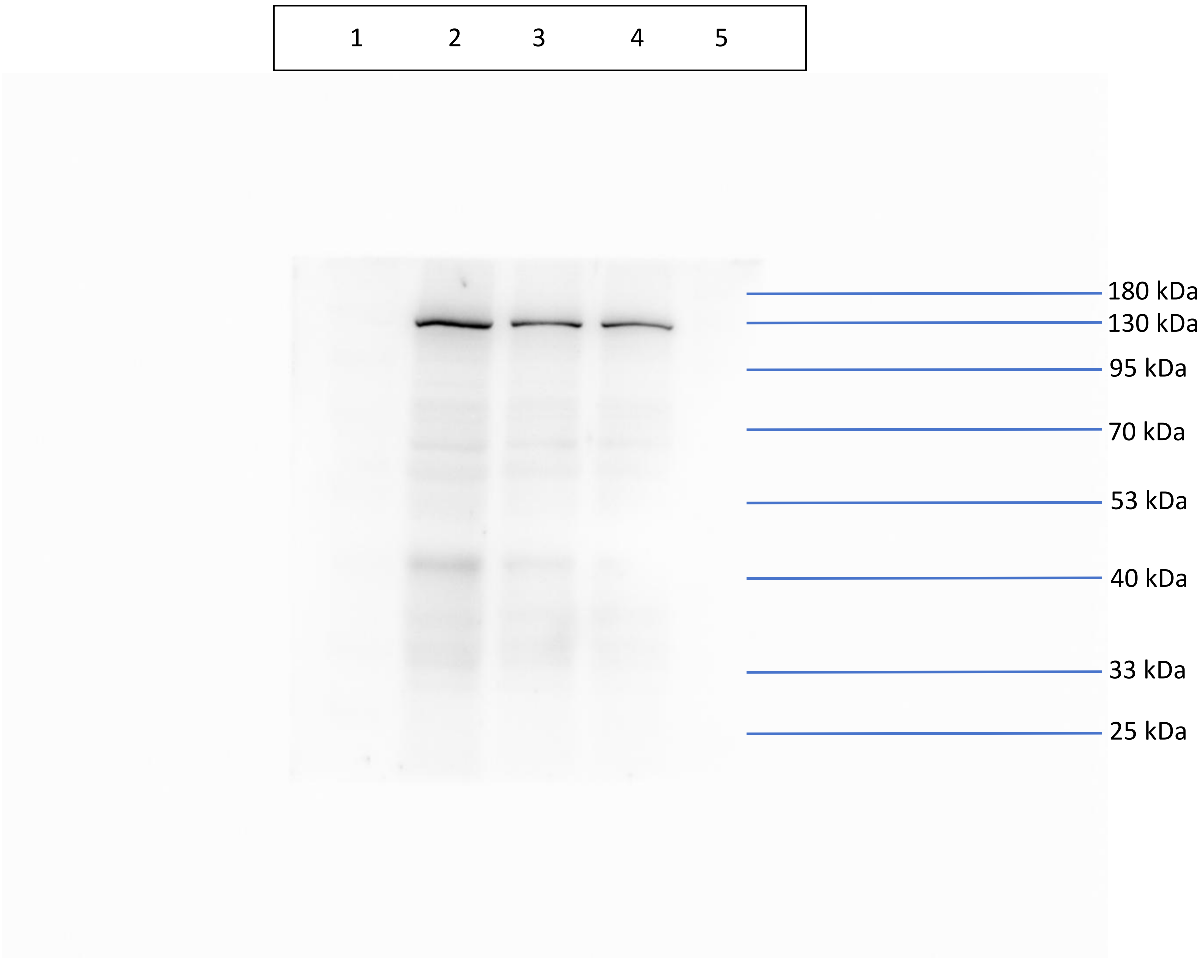

Lane1：Protein marker

Lane2：si-NC

Lane3：si-KIF18A-1

Lane4：si-KIF18A-2

Lane5：Protein marker

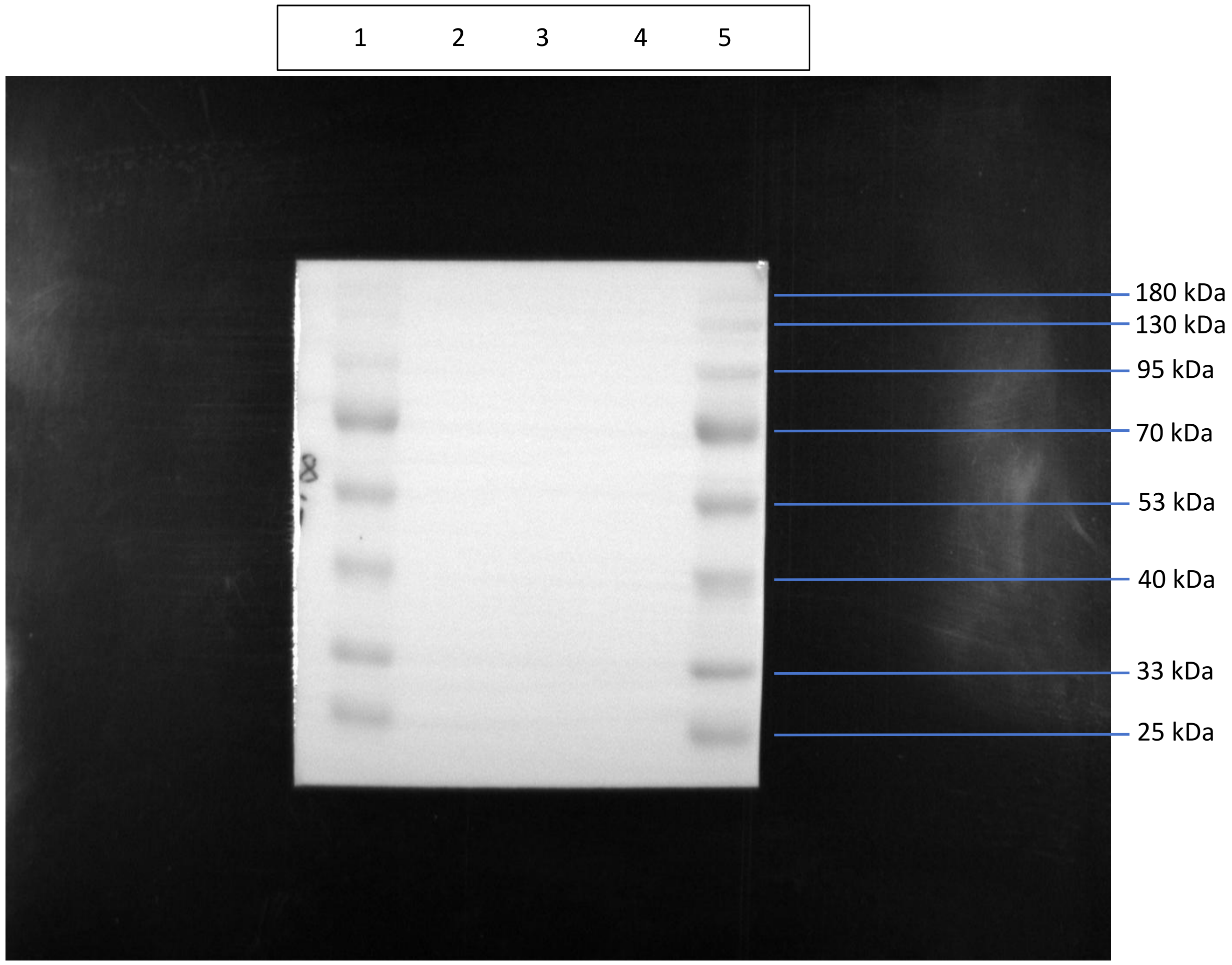

Lane1：Protein marker

Lane2：si-NC

Lane3：si-KIF18A-1

Lane4：si-KIF18A-2

Lane5：Protein marker

N-Cadherin

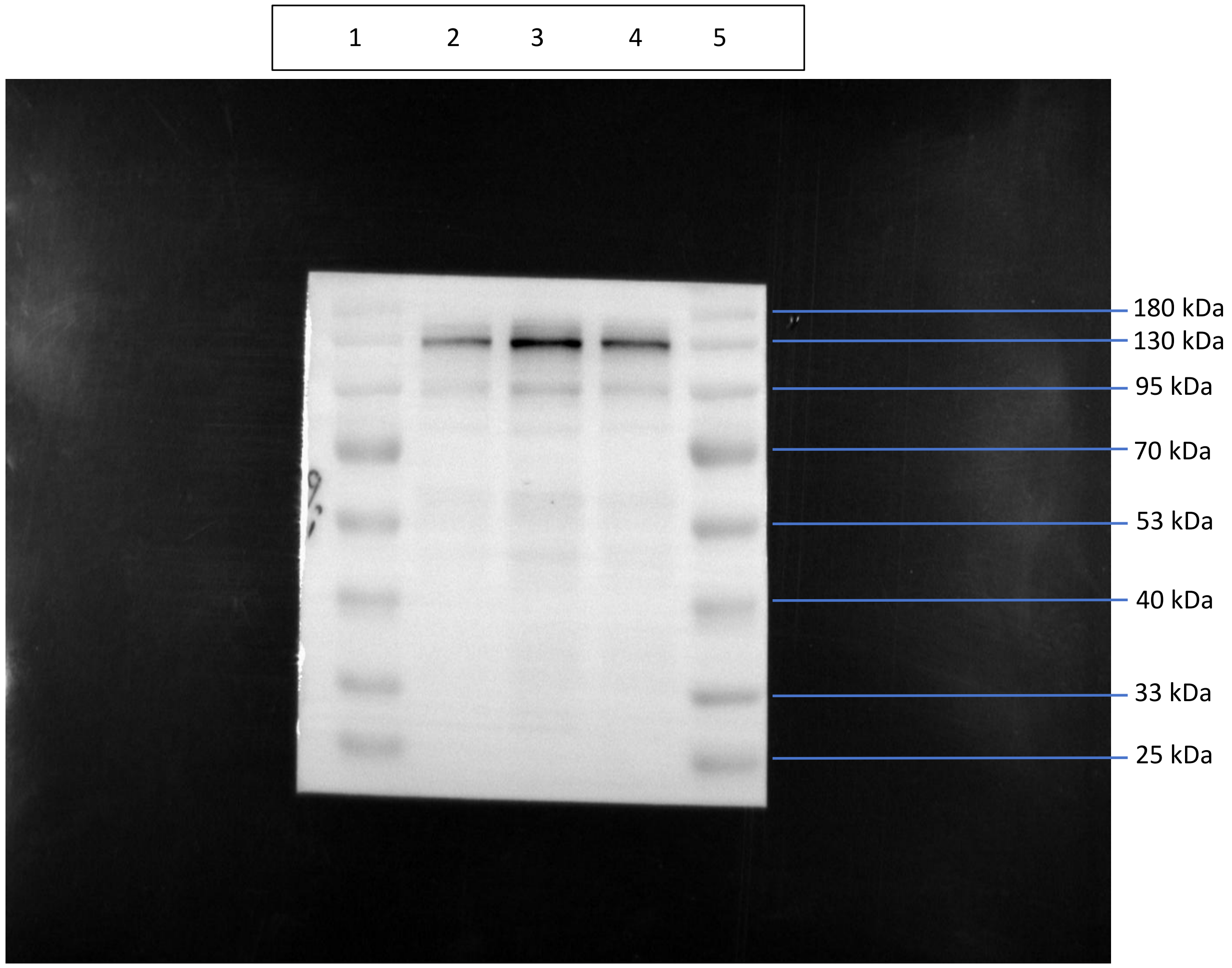

Lane1：Protein marker

Lane2：si-NC

Lane3：si-KIF18A-1

Lane4：si-KIF18A-2

Lane5：Protein marker

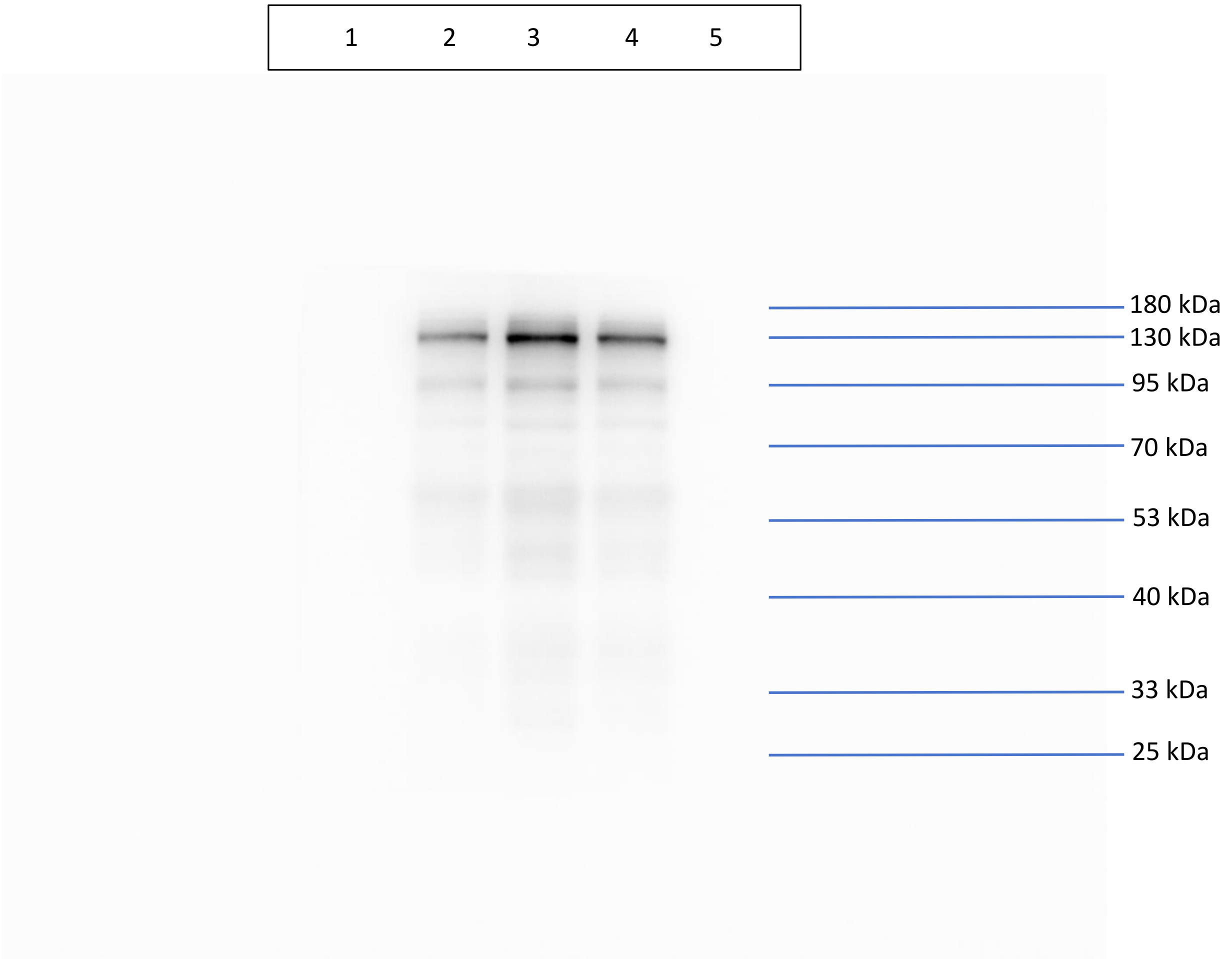

Lane1：Protein marker

Lane2：si-NC

Lane3：si-KIF18A-1

Lane4：si-KIF18A-2

Lane5：Protein marker

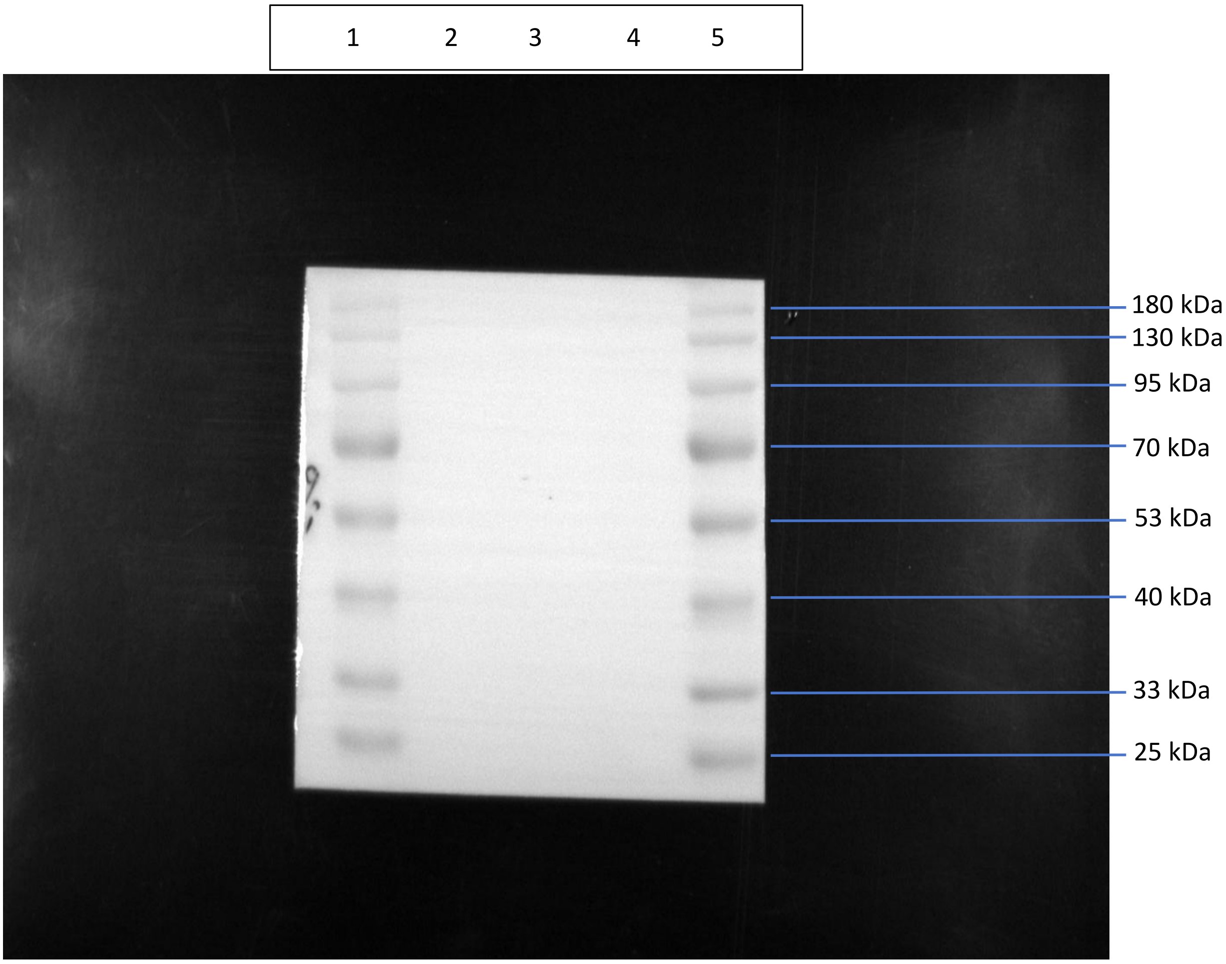

Lane1：Protein marker

Lane2：si-NC

Lane3：si-KIF18A-1

Lane4：si-KIF18A-2

Lane5：Protein marker

Snail1

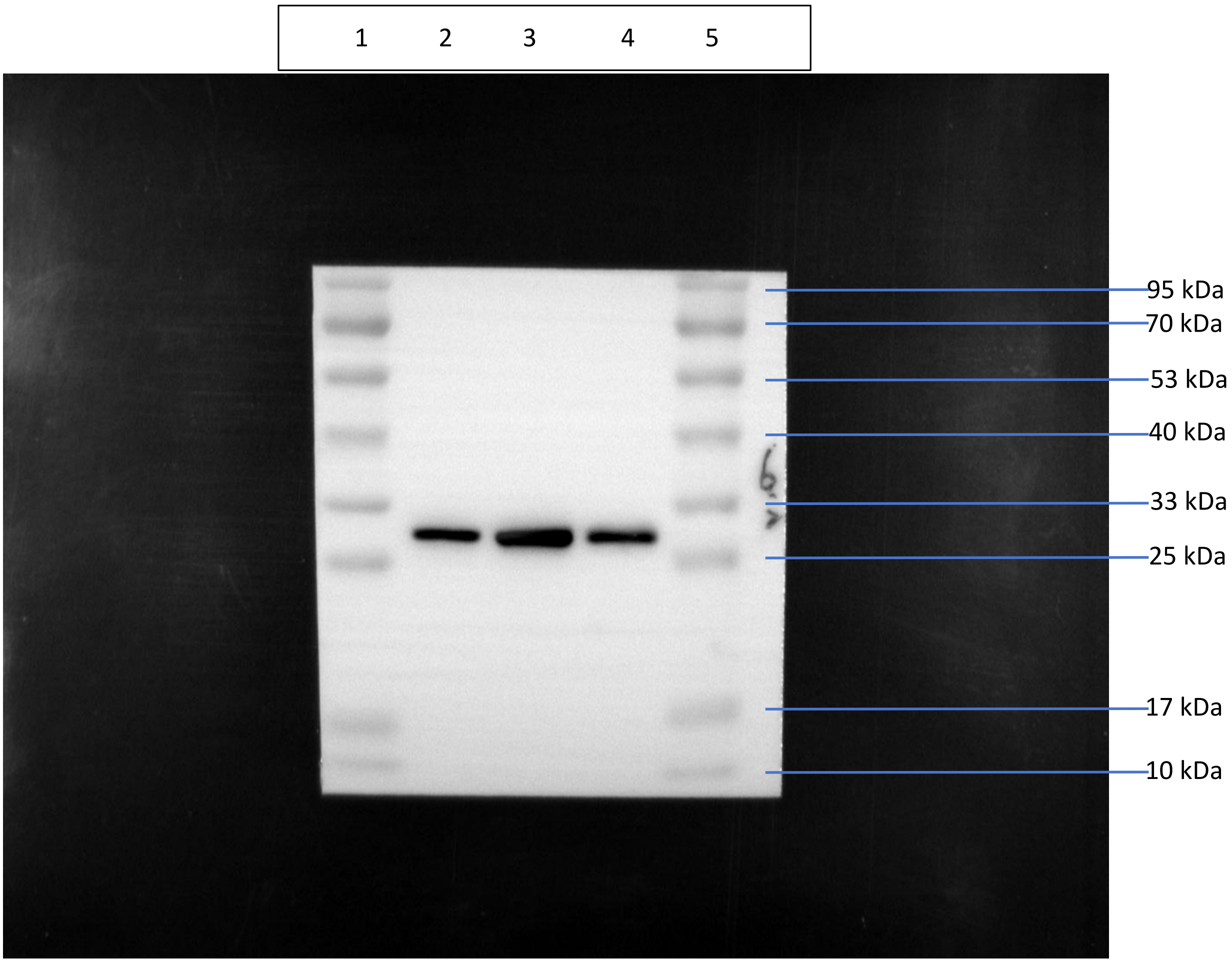

Lane1：Protein marker

Lane2：si-NC

Lane3：si-KIF18A-1

Lane4：si-KIF18A-2

Lane5：Protein marker

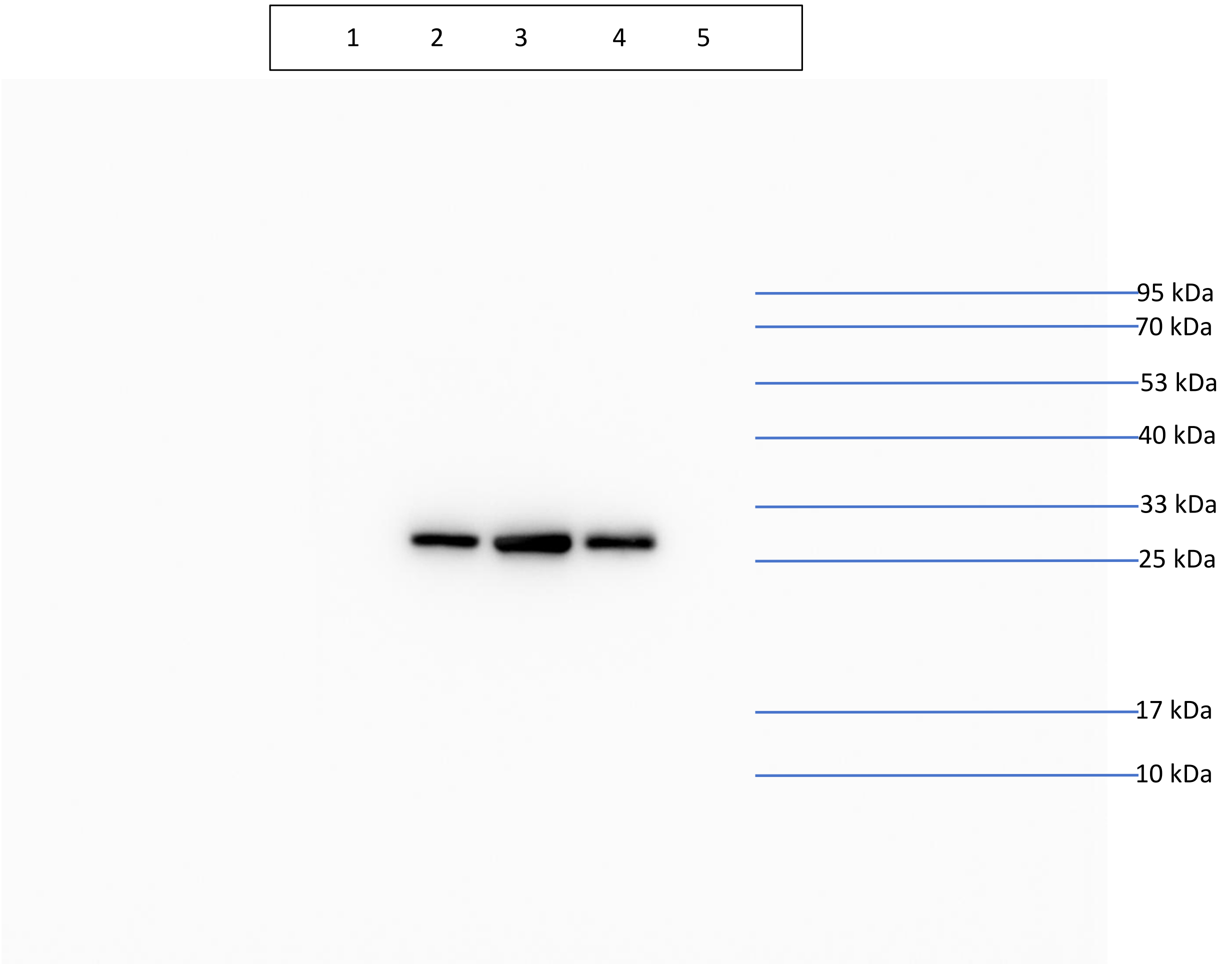

Lane1：Protein marker

Lane2：si-NC

Lane3：si-KIF18A-1

Lane4：si-KIF18A-2

Lane5：Protein marker

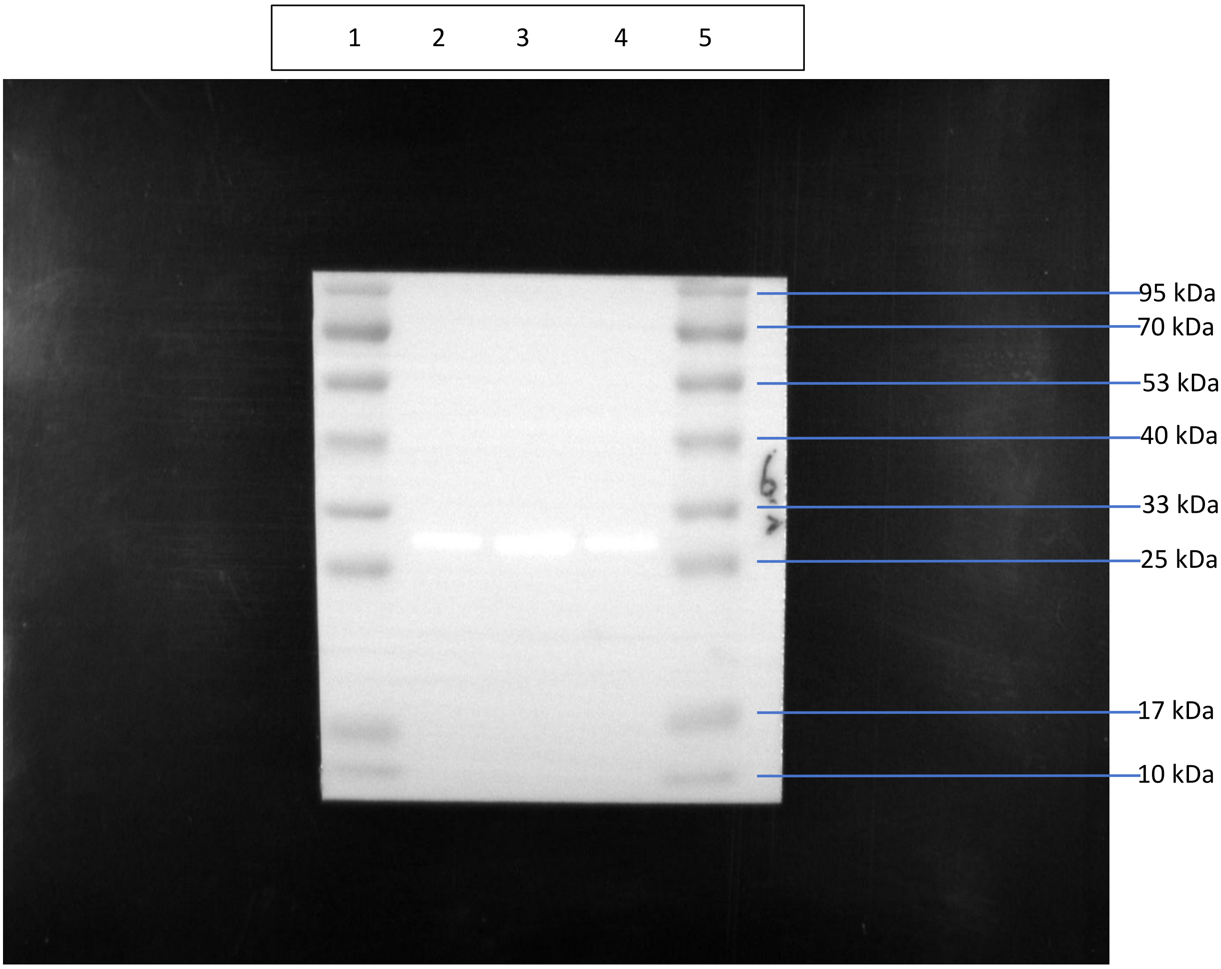

Lane1：Protein marker

Lane2：si-NC

Lane3：si-KIF18A-1

Lane4：si-KIF18A-2

Lane5：Protein marker

Vimentin

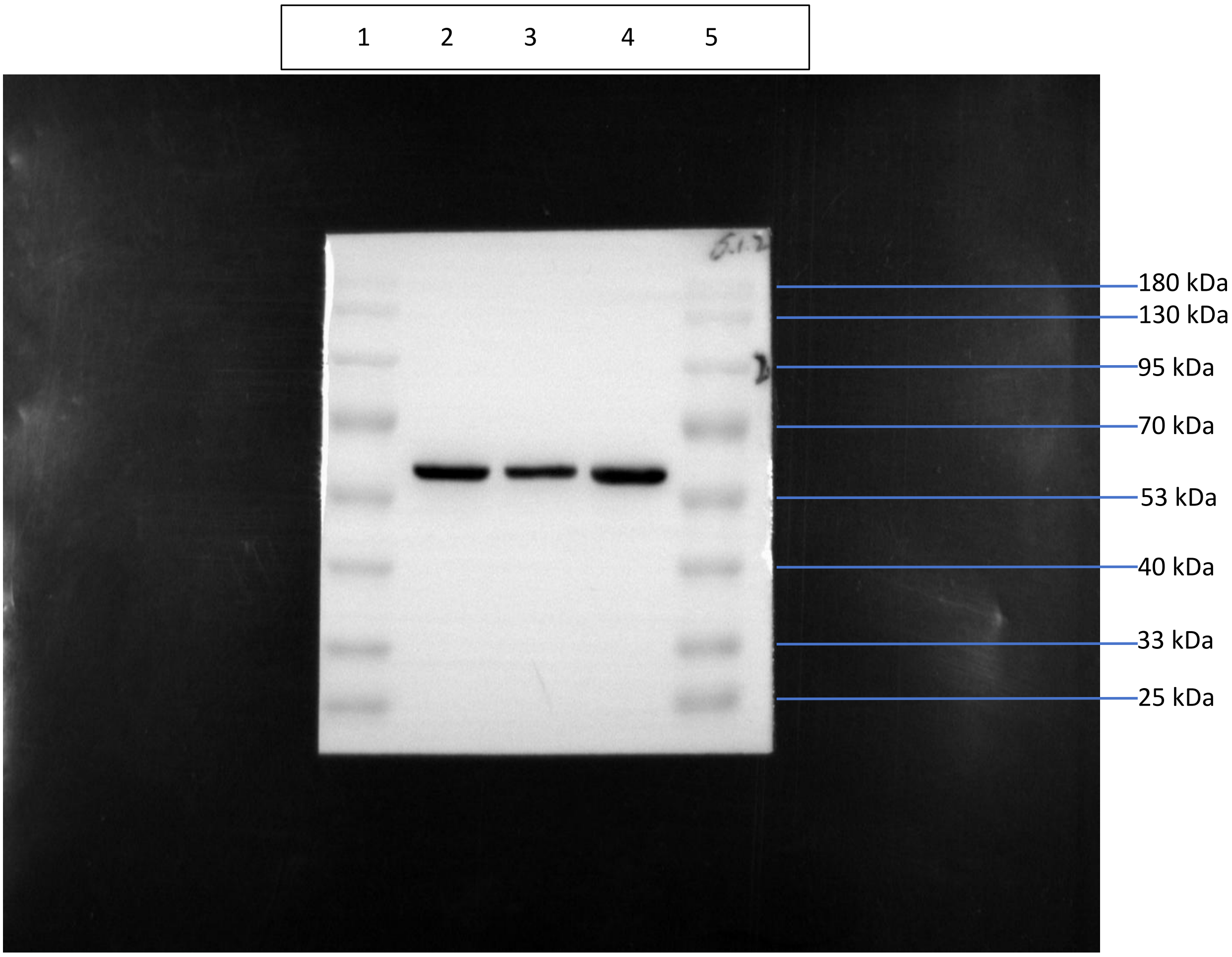

Lane1：Protein marker

Lane2：si-NC

Lane3：si-KIF18A-1

Lane4：si-KIF18A-2

Lane5：Protein marker

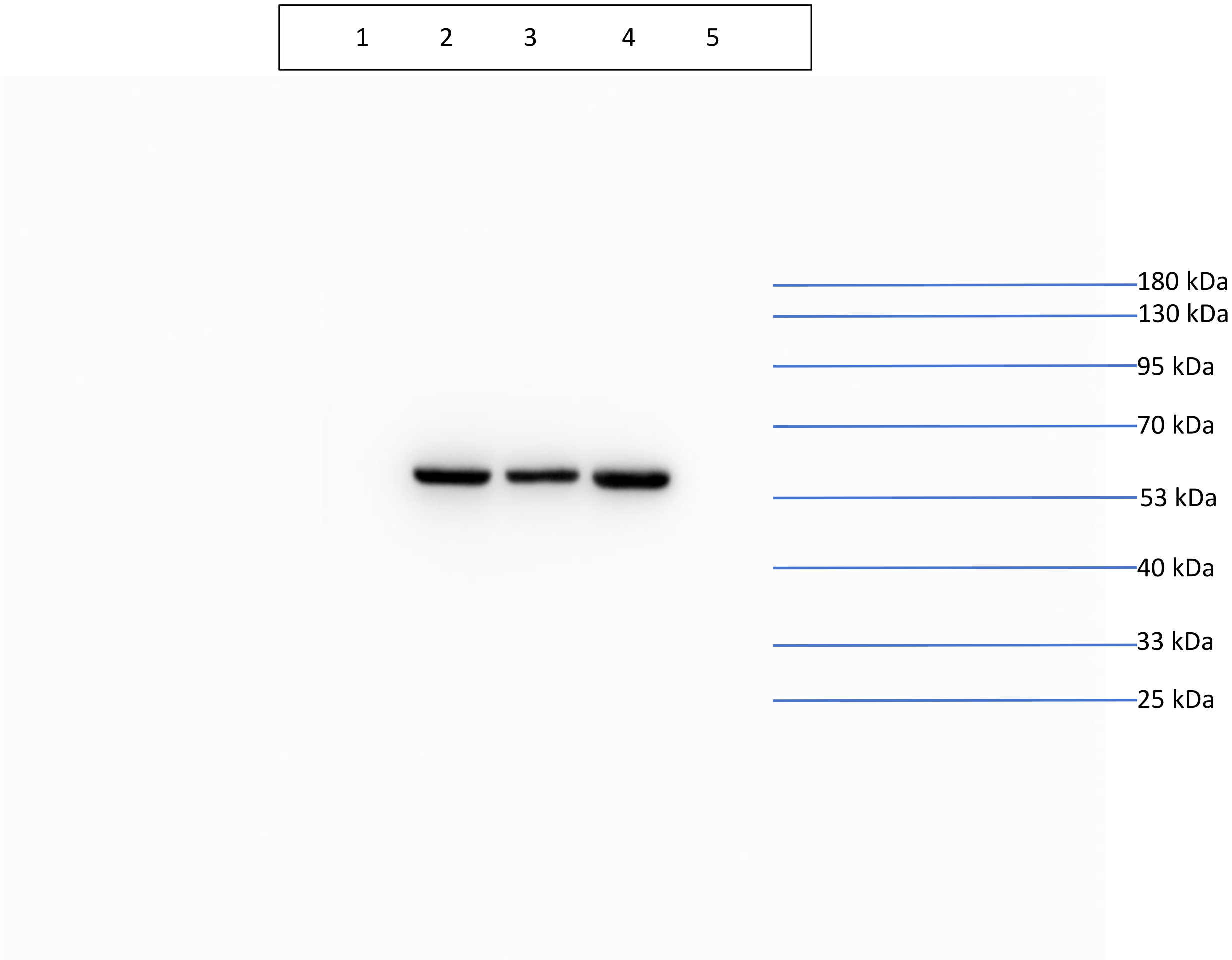

Lane1：Protein marker

Lane2：si-NC

Lane3：si-KIF18A-1

Lane4：si-KIF18A-2

Lane5：Protein marker

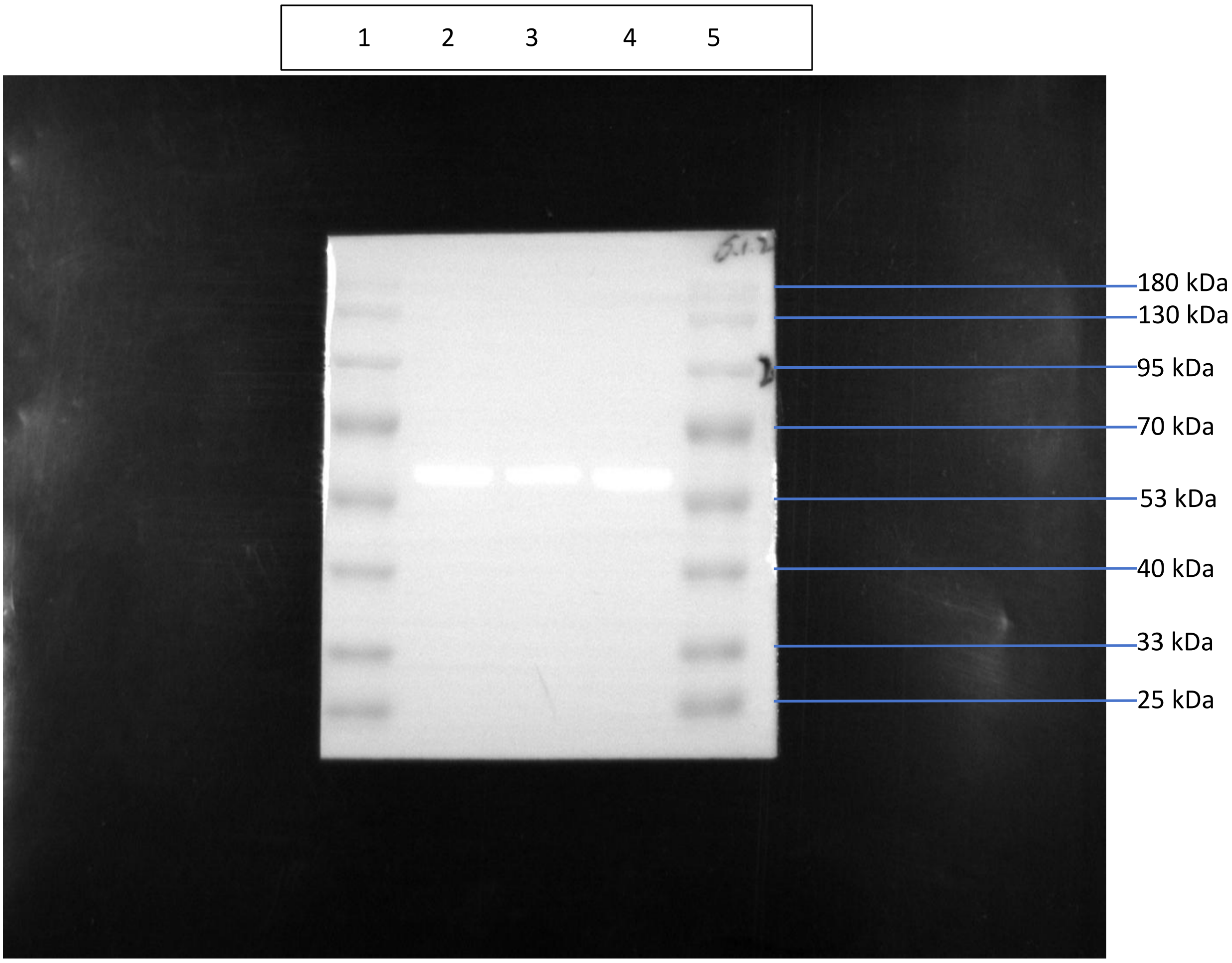

Lane1：Protein marker

Lane2：si-NC

Lane3：si-KIF18A-1

Lane4：si-KIF18A-2

Lane5：Protein marker

GAPDH

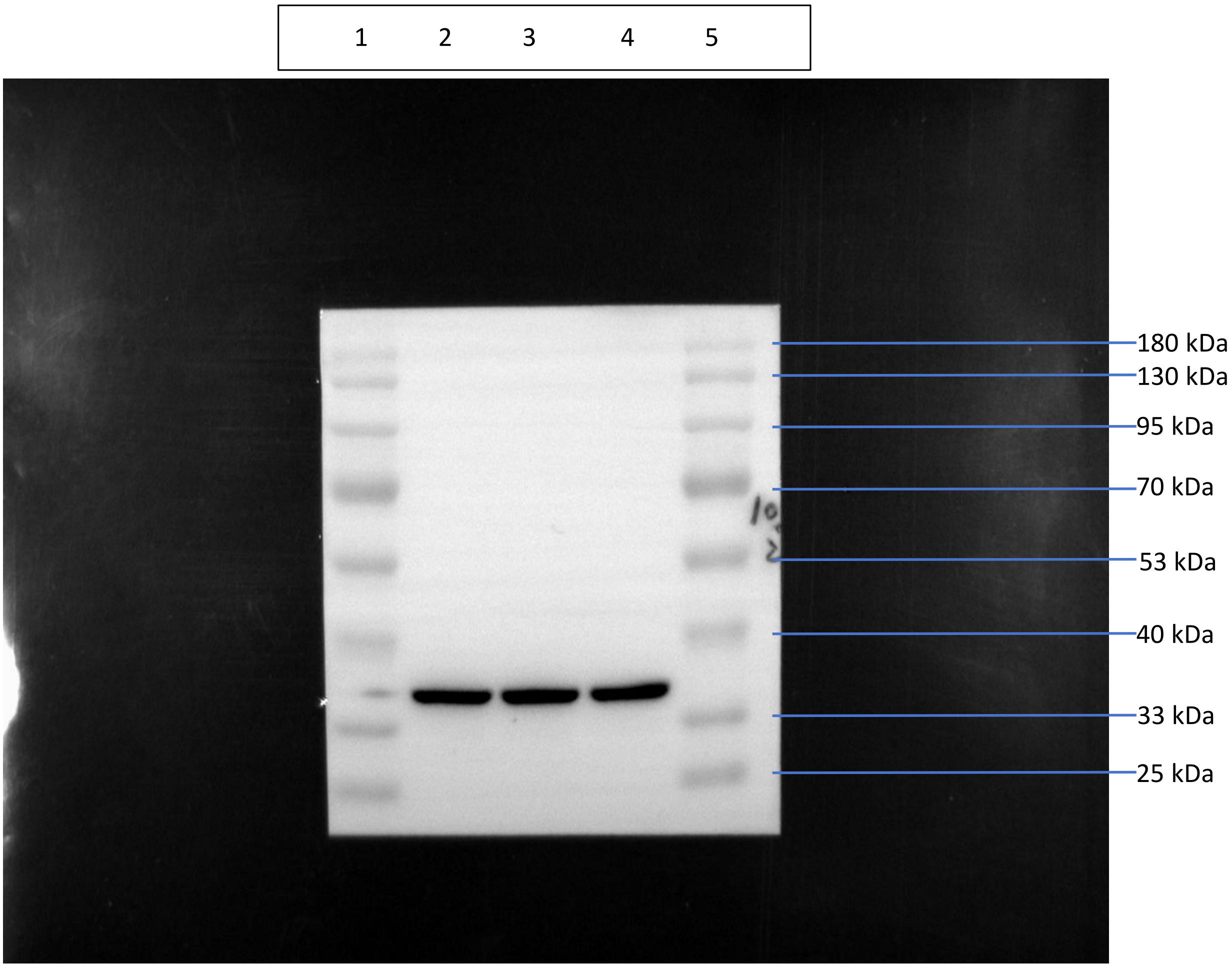

Lane1：Protein marker

Lane2：si-NC

Lane3：si-KIF18A-1

Lane4：si-KIF18A-2

Lane5：Protein marker

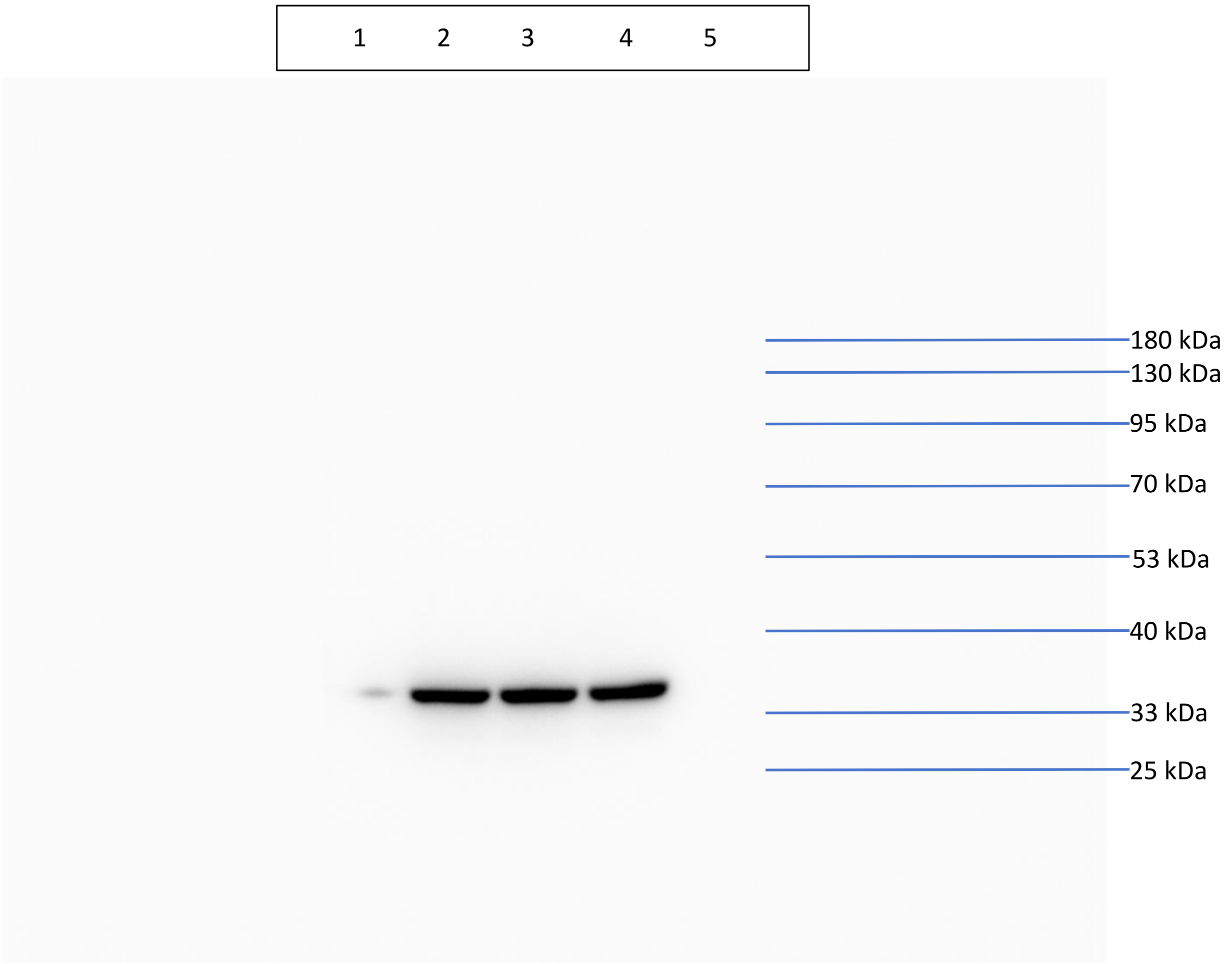

Lane1：Protein marker

Lane2：si-NC

Lane3：si-KIF18A-1

Lane4：si-KIF18A-2

Lane5：Protein marker

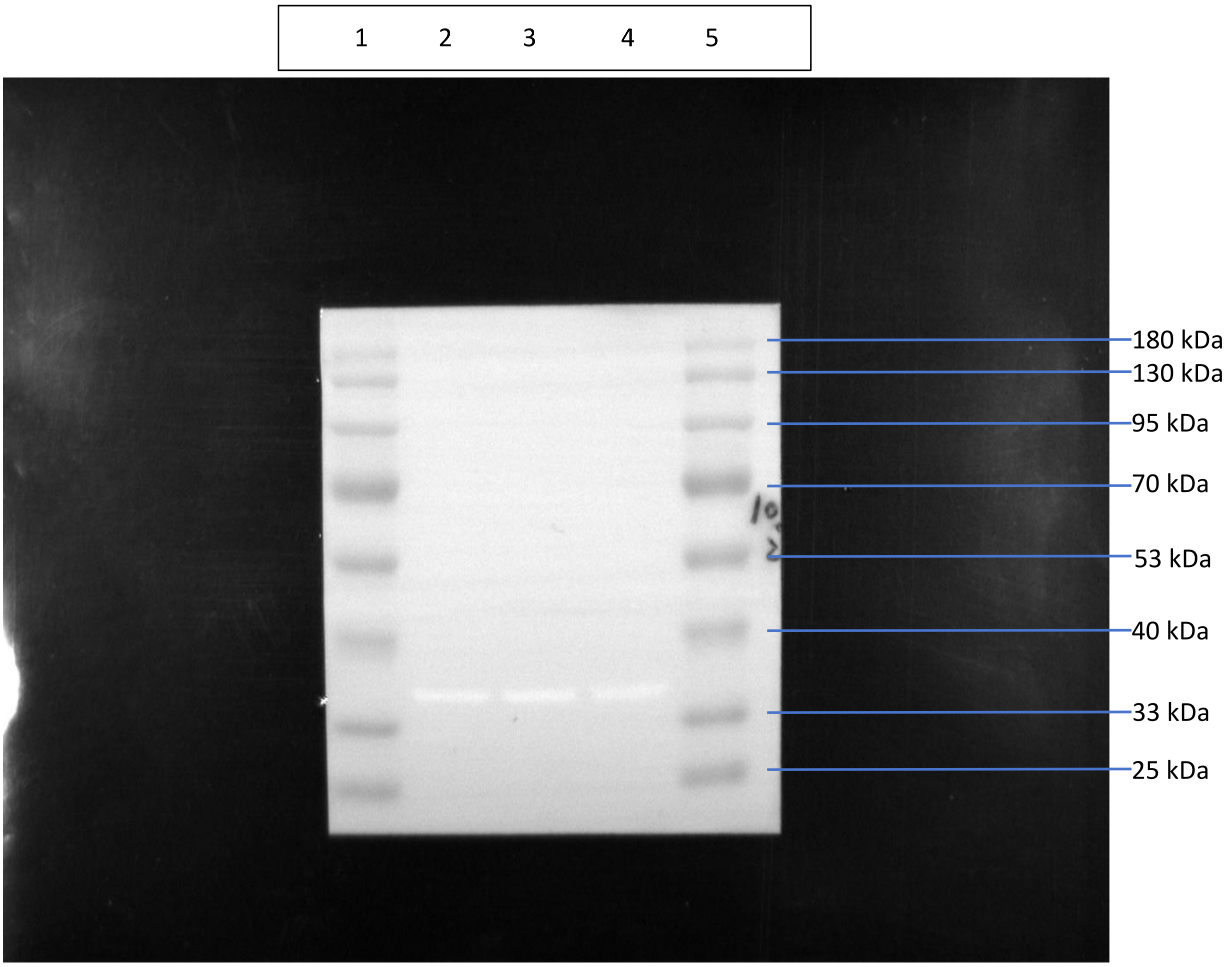

Lane1：Protein marker

Lane2：si-NC

Lane3：si-KIF18A-1

Lane4：si-KIF18A-2

Lane5：Protein marker

Figure6-A

5-LOX

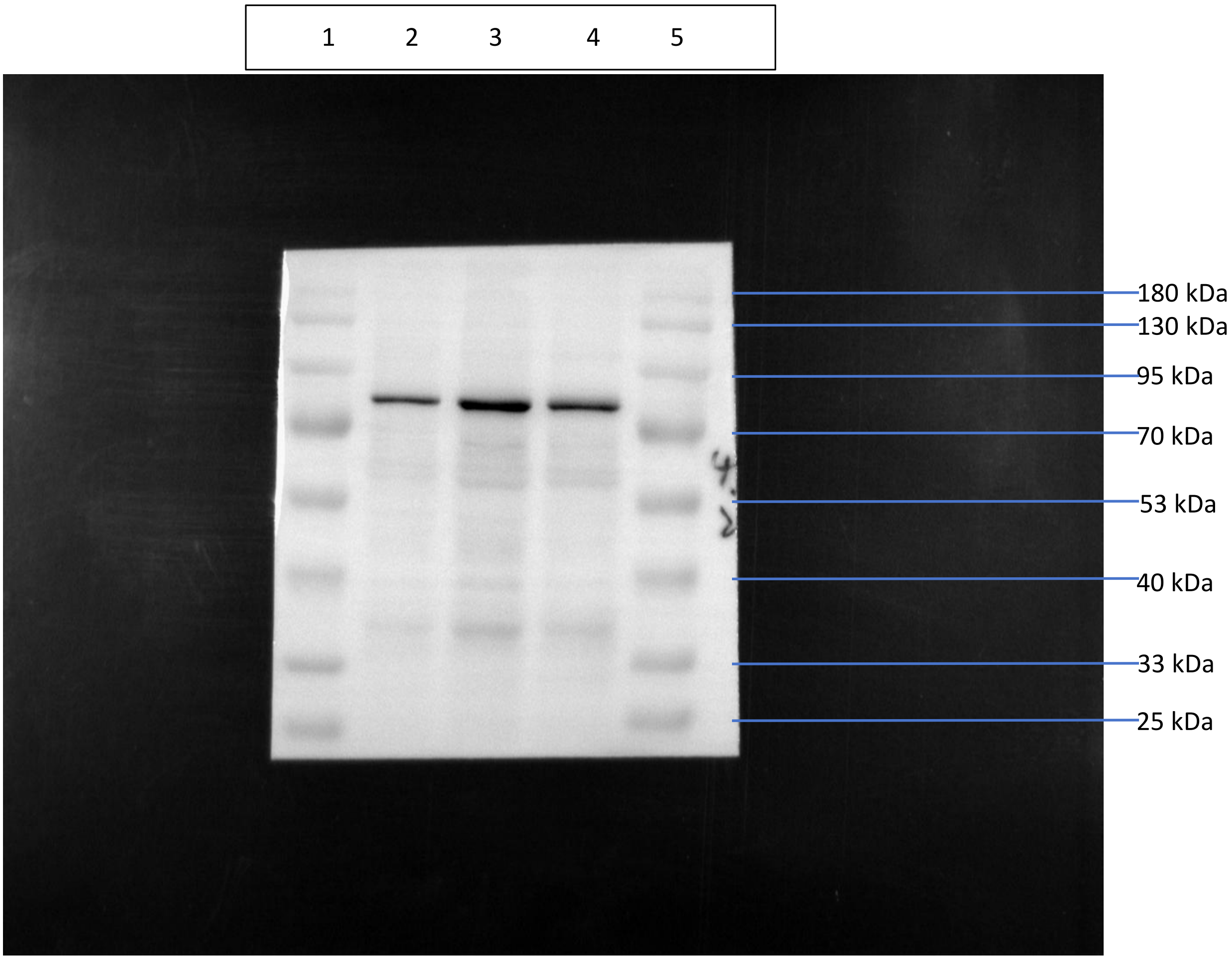

Lane1：Protein marker

Lane2：Vector

Lane3：oe-KIF18A

Lane4：oe-KIF18A+U73122

Lane5：Protein marker

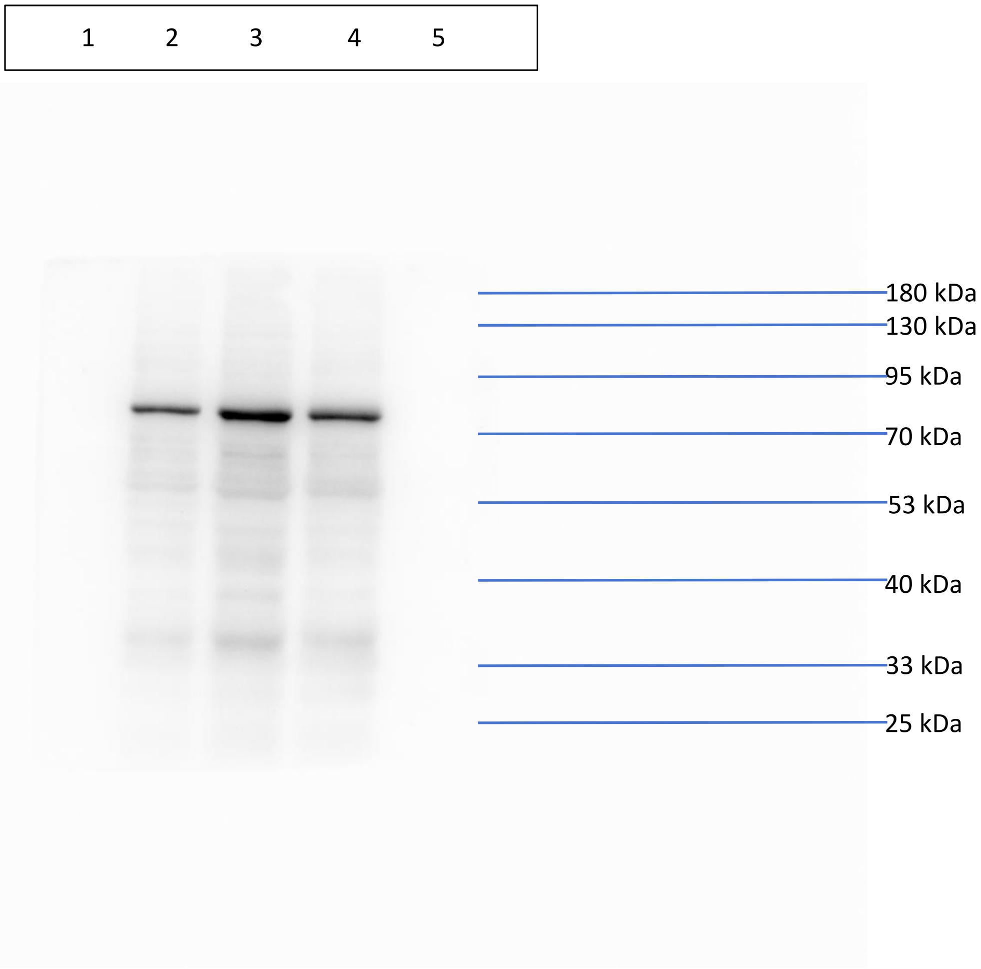

Lane1：Protein marker

Lane2：Vector

Lane3：oe-KIF18A

Lane4：oe-KIF18A+U73122

Lane5：Protein marker

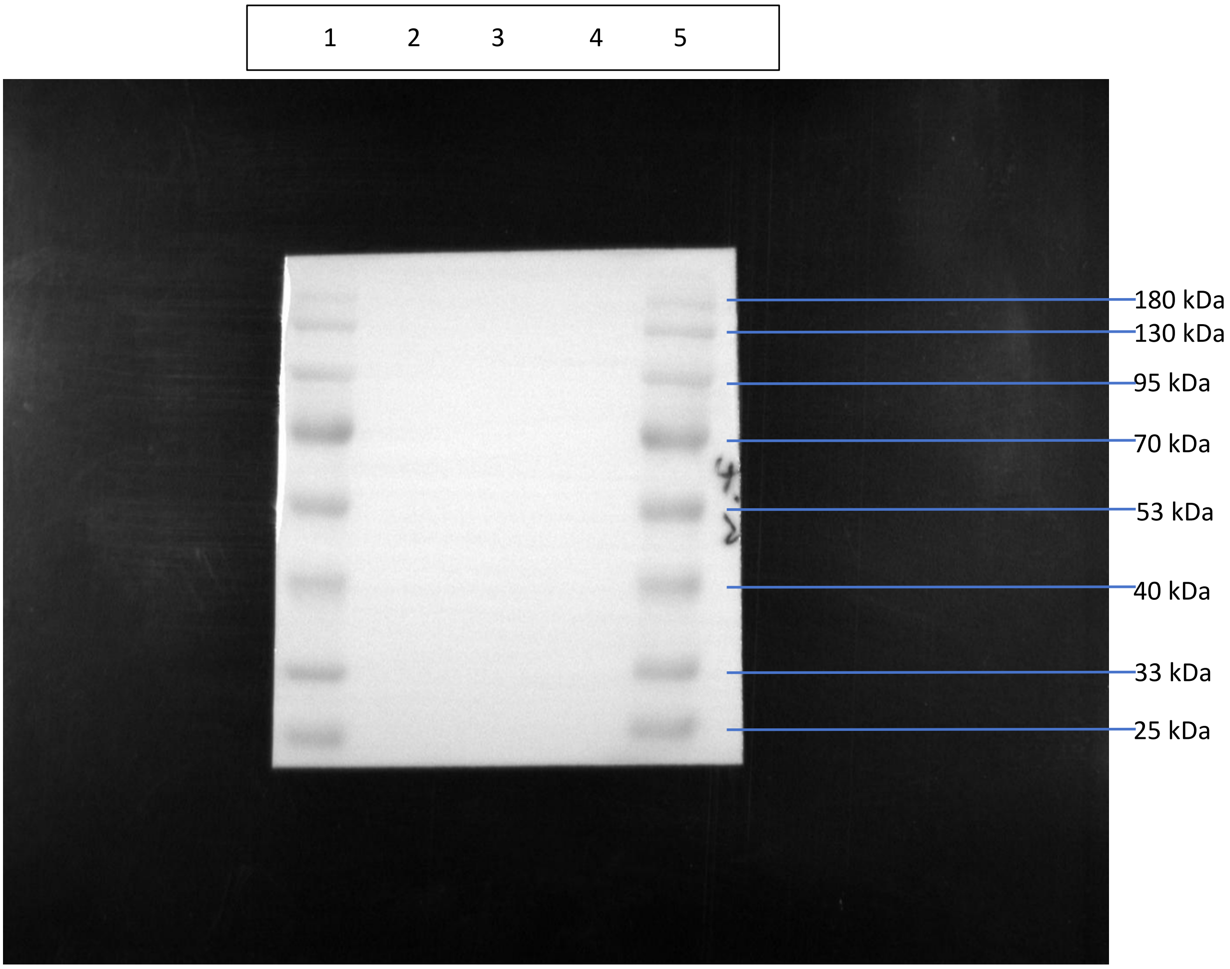

Lane1：Protein marker

Lane2：Vector

Lane3：oe-KIF18A

Lane4：oe-KIF18A+U73122

Lane5：Protein marker

GAPDH

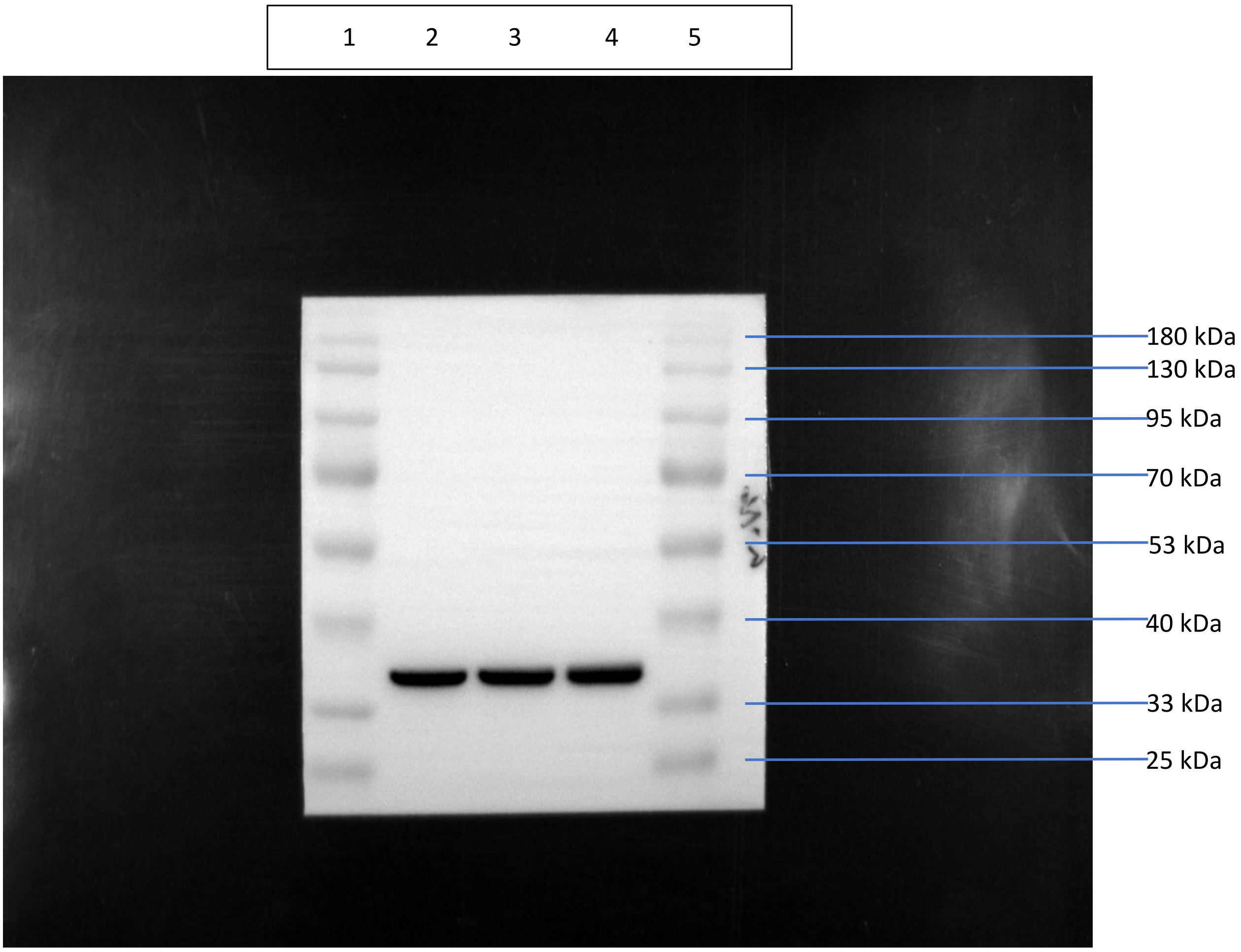

Lane1：Protein marker

Lane2：Vector

Lane3：oe-KIF18A

Lane4：oe-KIF18A+U73122

Lane5：Protein marker

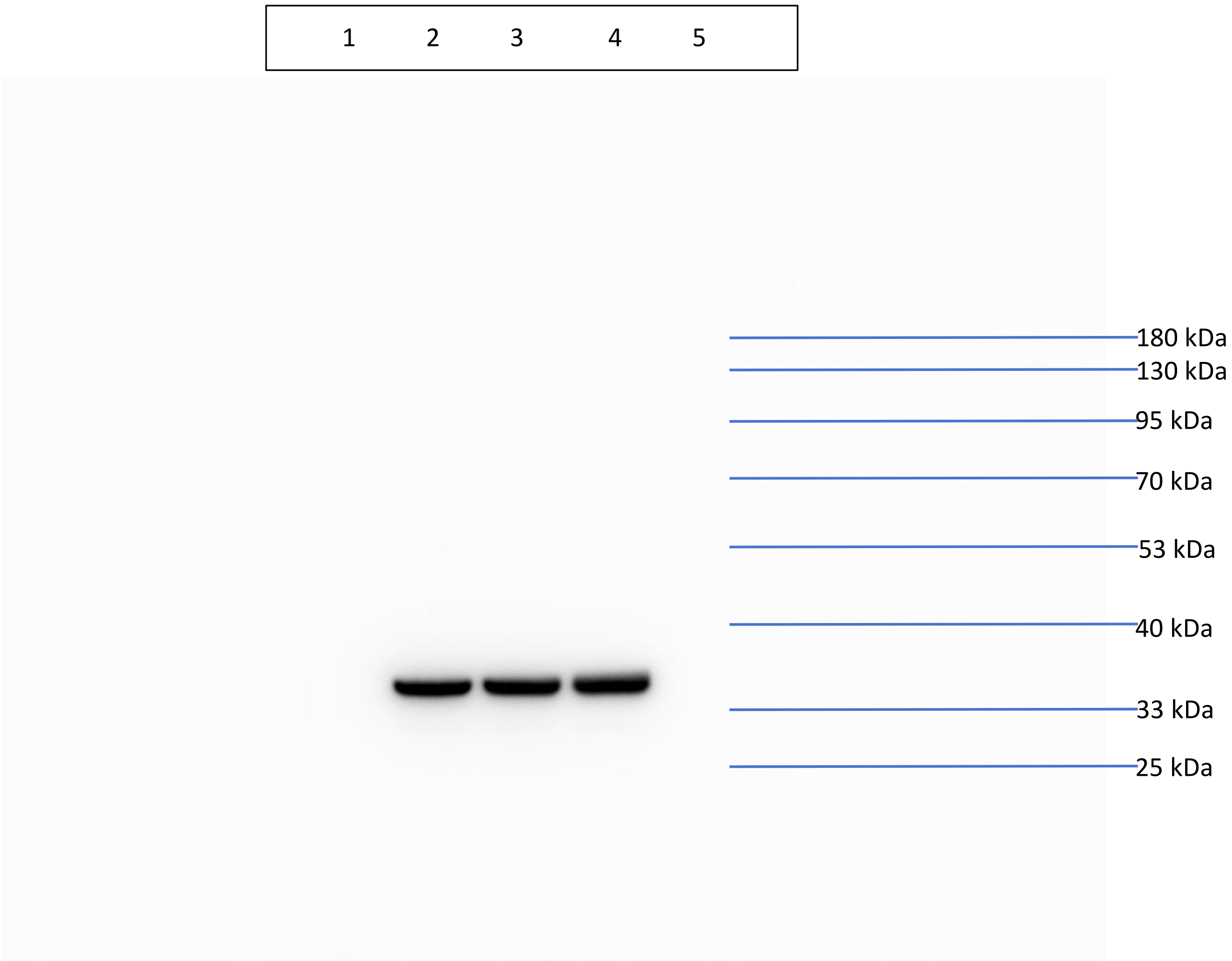

Lane1：Protein marker

Lane2：Vector

Lane3：oe-KIF18A

Lane4：oe-KIF18A+U73122

Lane5：Protein marker

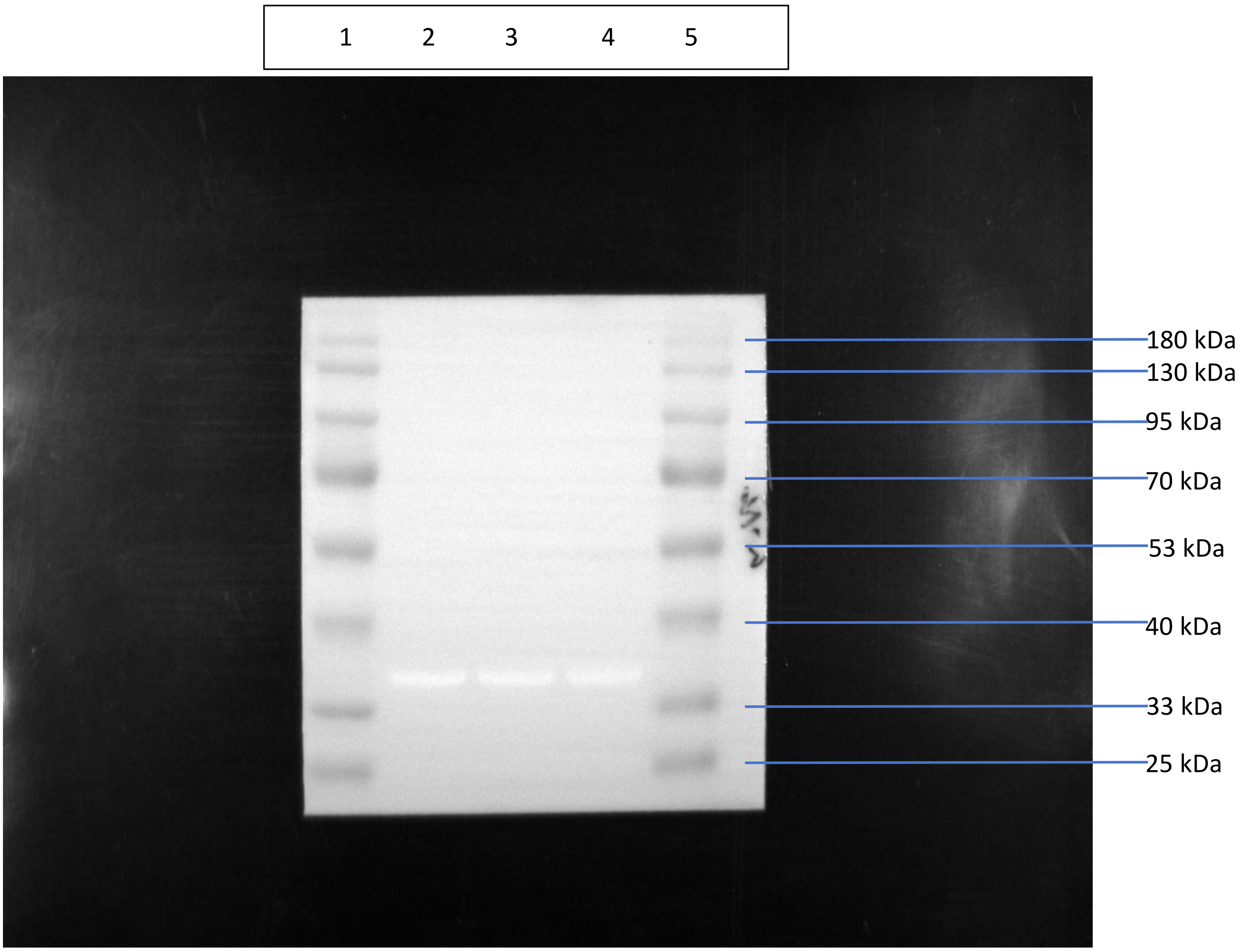

Lane1：Protein marker

Lane2：Vector

Lane3：oe-KIF18A

Lane4：oe-KIF18A+U73122

Lane5：Protein marker

Figure6-B

5-LOX

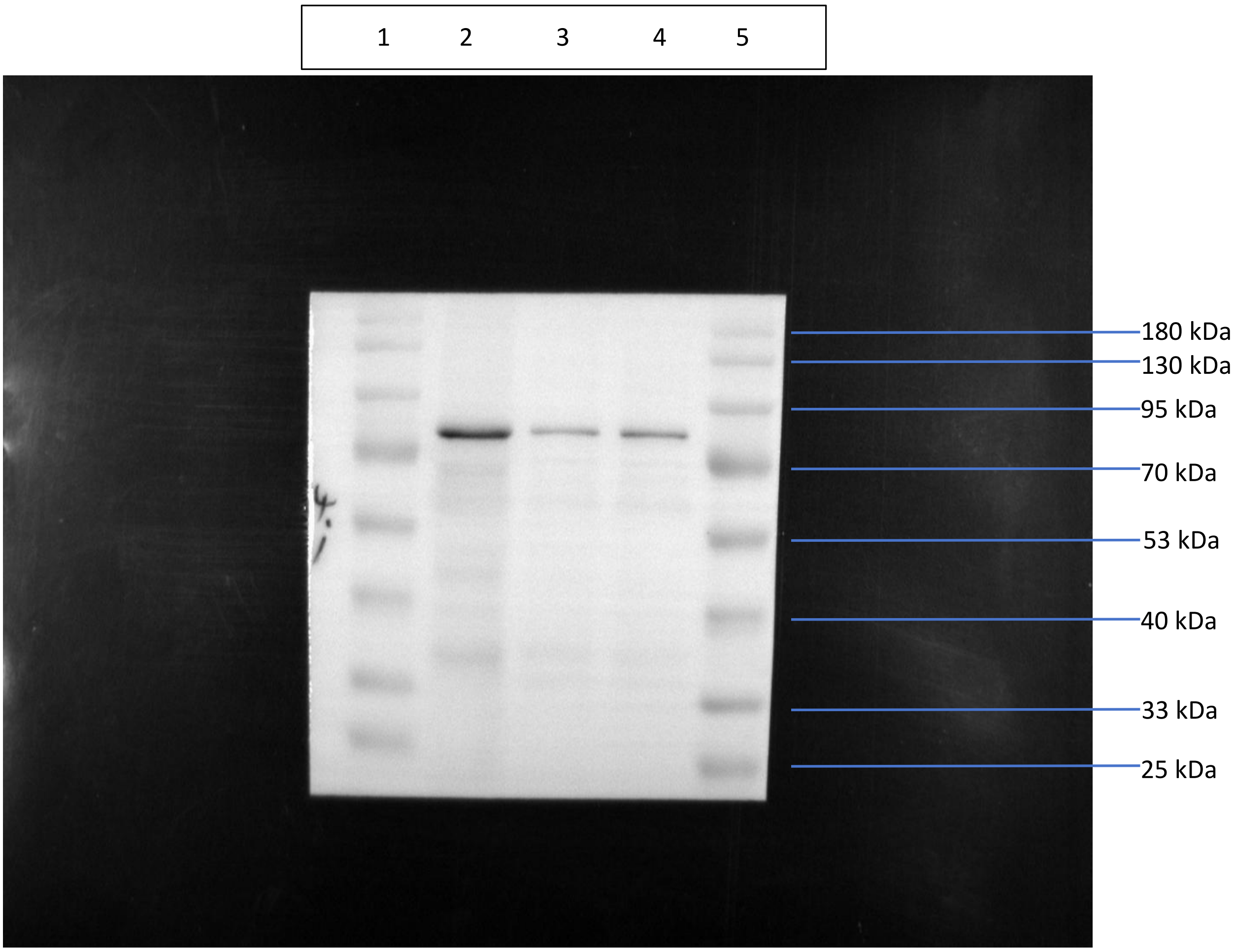

Lane1：Protein marker

Lane2：si-NC

Lane3：si-KIF18A-1

Lane4：si-KIF18A-2

Lane5：Protein marker

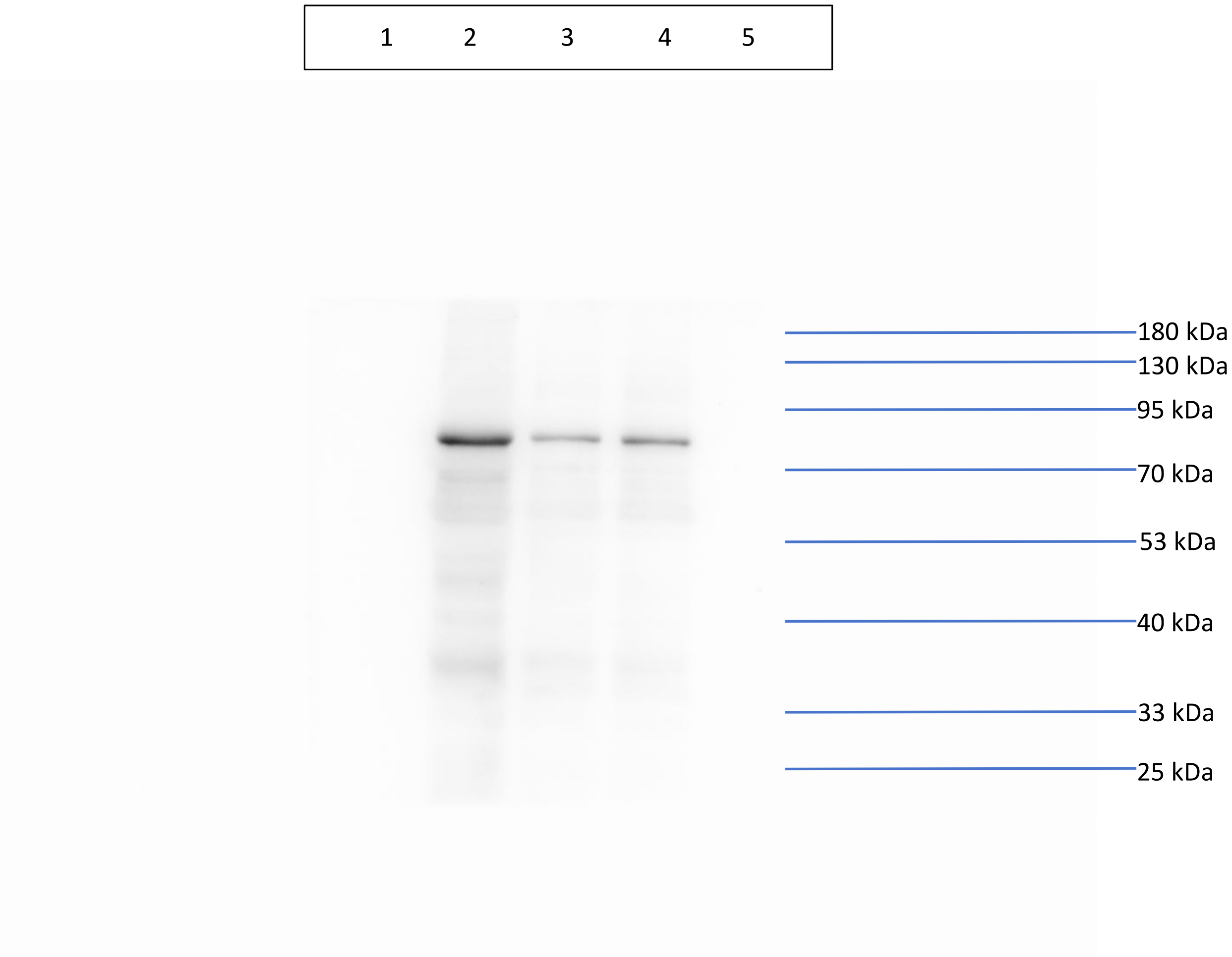

Lane1：Protein marker

Lane2：si-NC

Lane3：si-KIF18A-1

Lane4：si-KIF18A-2

Lane5：Protein marker

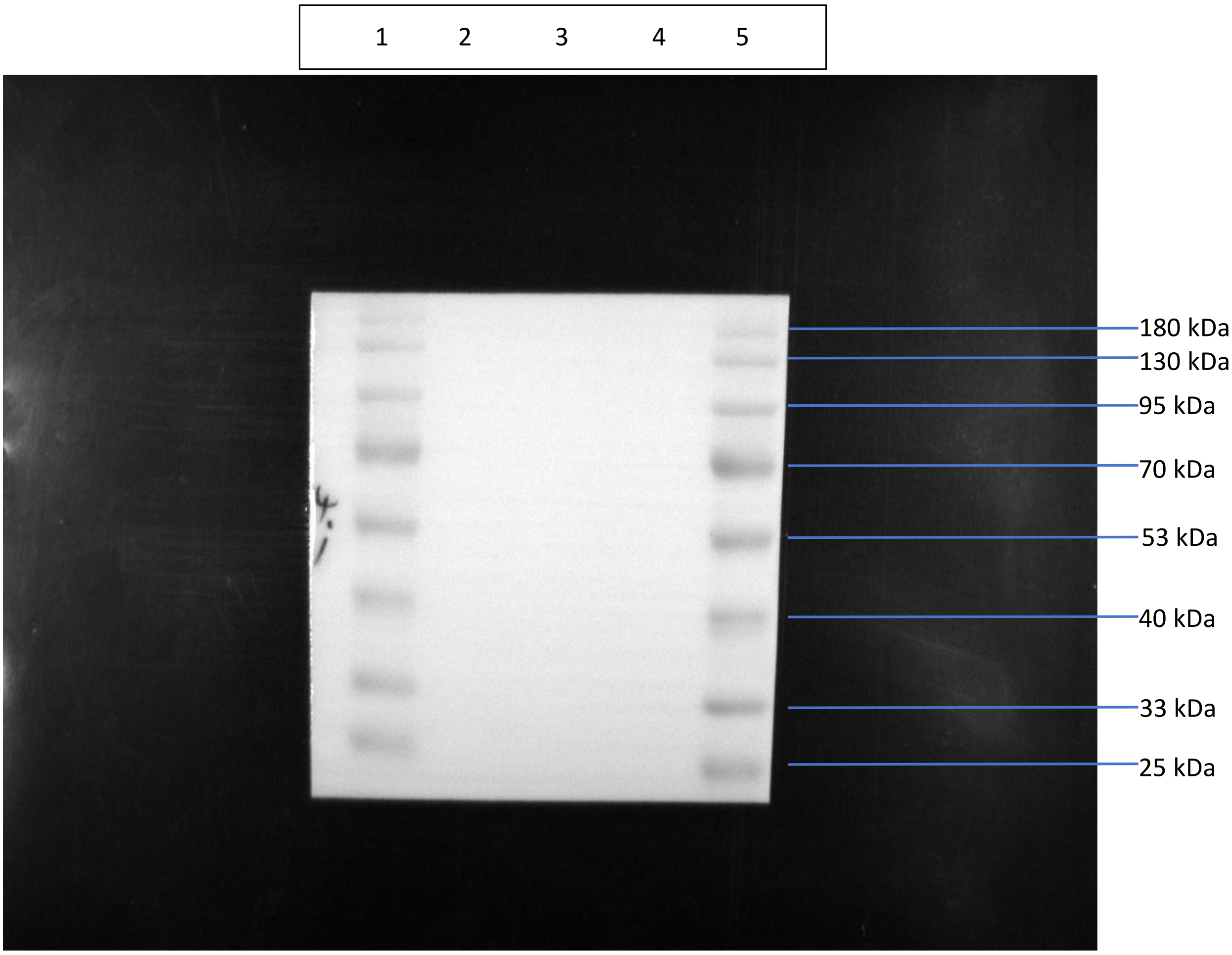

Lane1：Protein marker

Lane2：si-NC

Lane3：si-KIF18A-1

Lane4：si-KIF18A-2

Lane5：Protein marker

GAPDH

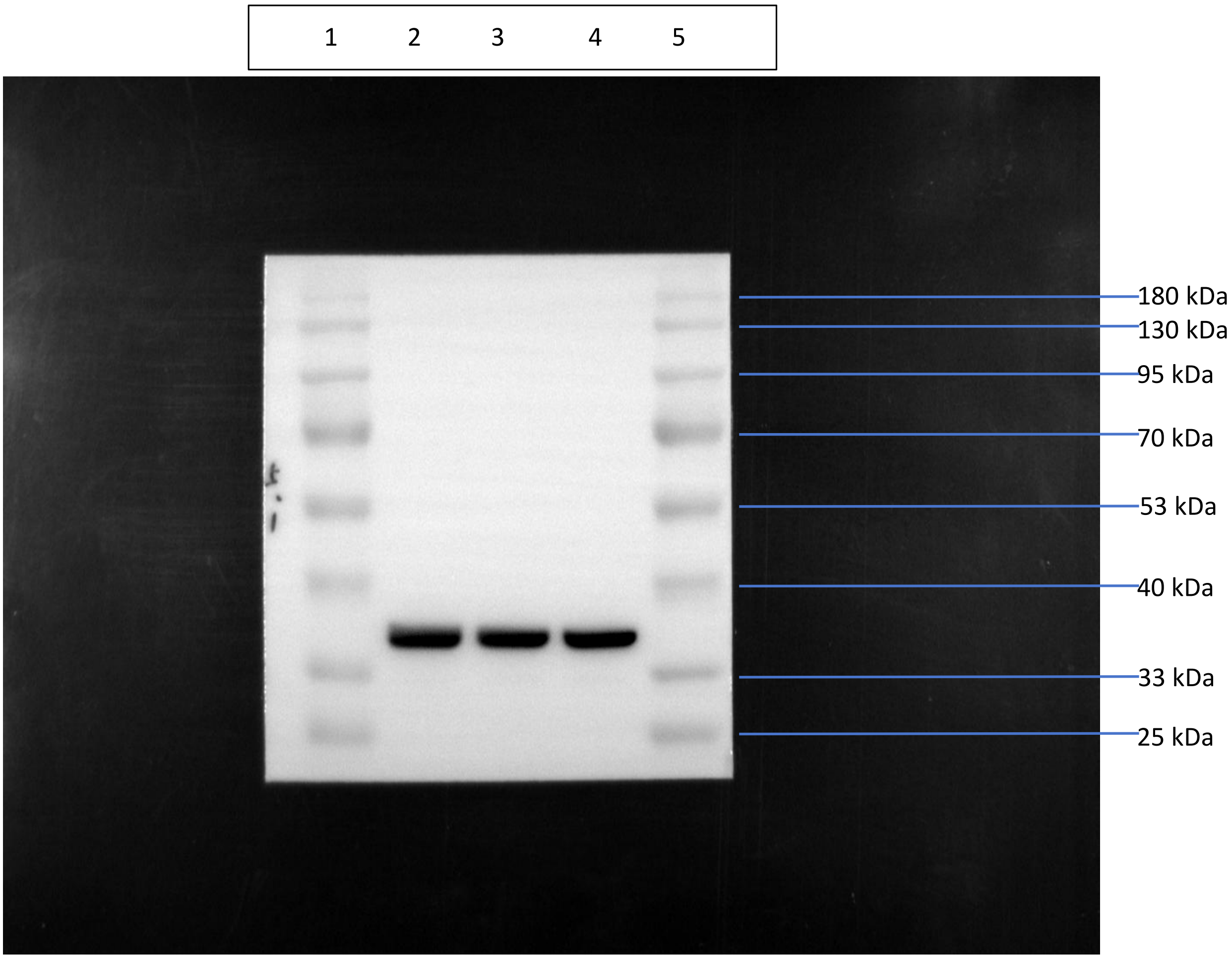

Lane1：Protein marker

Lane2：si-NC

Lane3：si-KIF18A-1

Lane4：si-KIF18A-2

Lane5：Protein marker

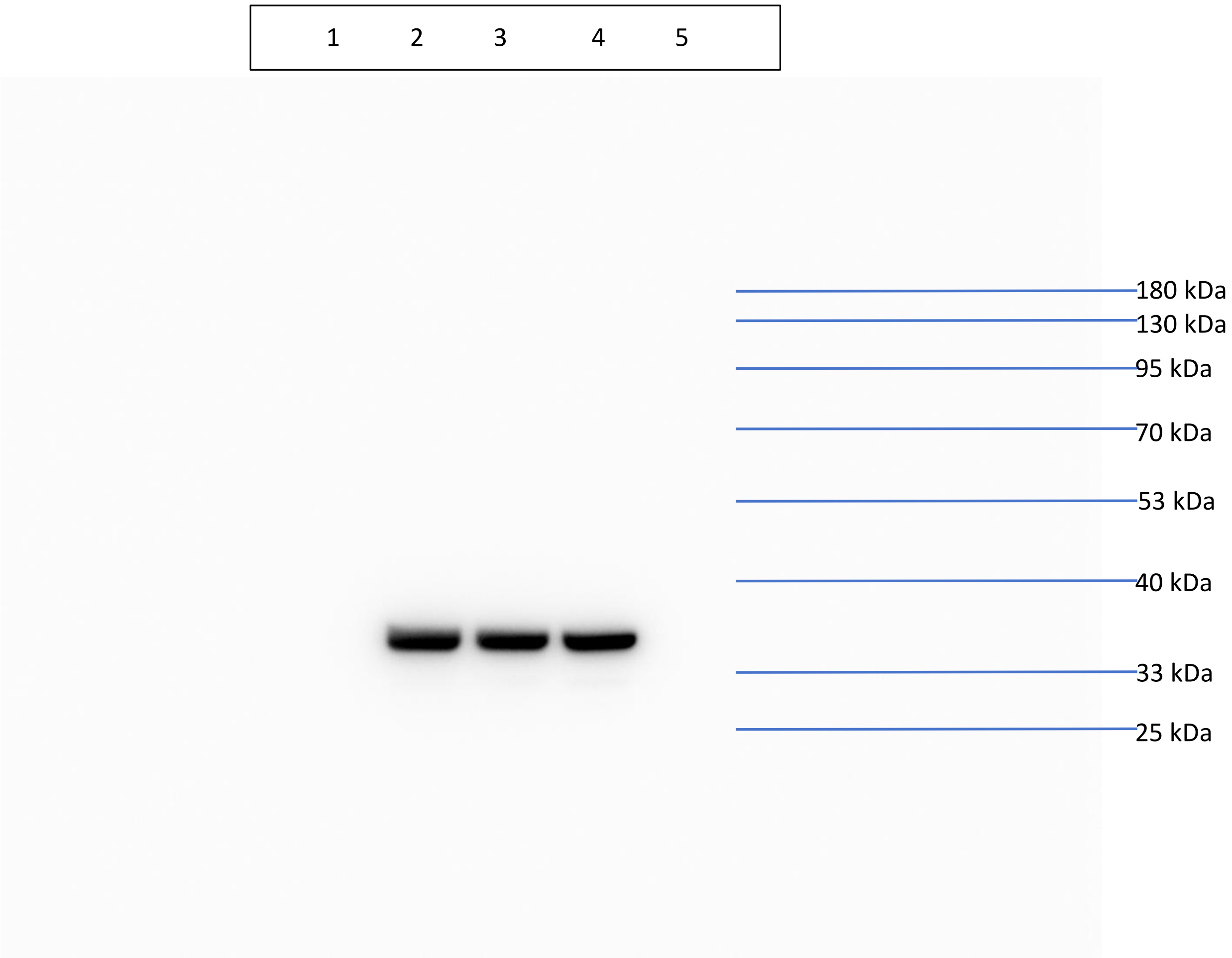

Lane1：Protein marker

Lane2：si-NC

Lane3：si-KIF18A-1

Lane4：si-KIF18A-2

Lane5：Protein marker

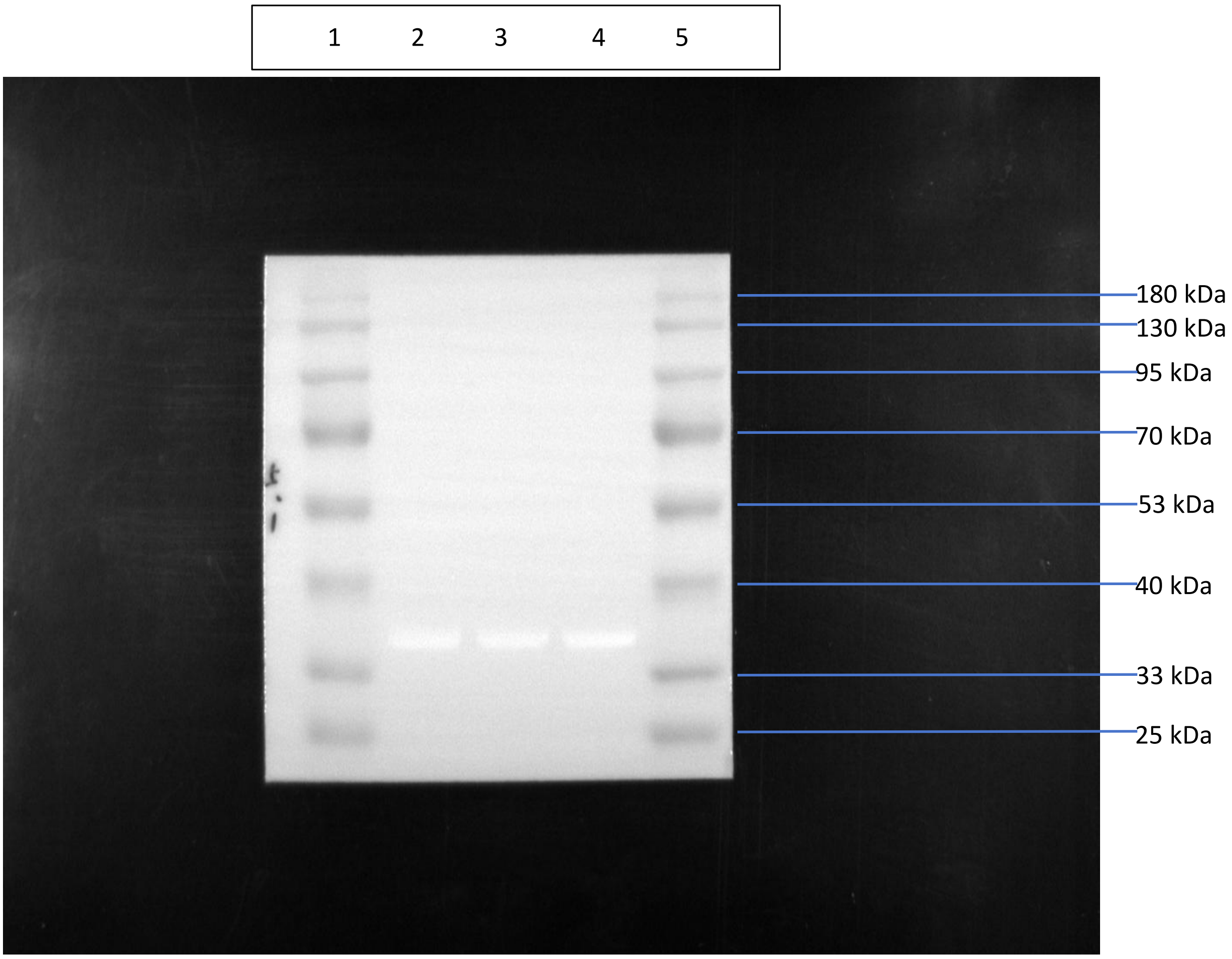

Lane1：Protein marker

Lane2：si-NC

Lane3：si-KIF18A-1

Lane4：si-KIF18A-2

Lane5：Protein marker

Figure6-E

5-LOX

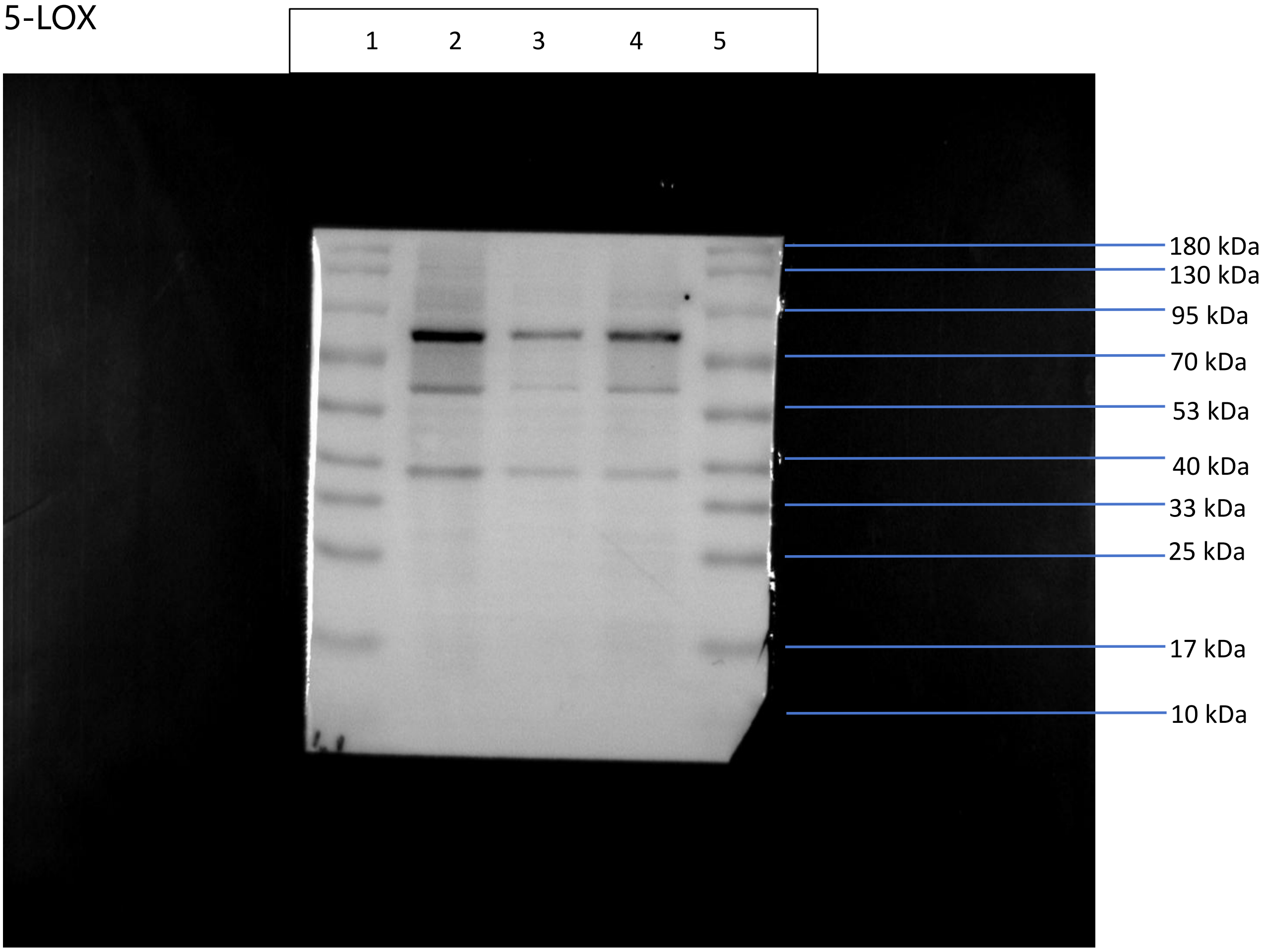

Lane1：Protein marker

Lane2：si-NC

Lane3：si-KIF18A-1

Lane4：si-KIF18A-1+oe5-LOX

Lane5：Protein marker

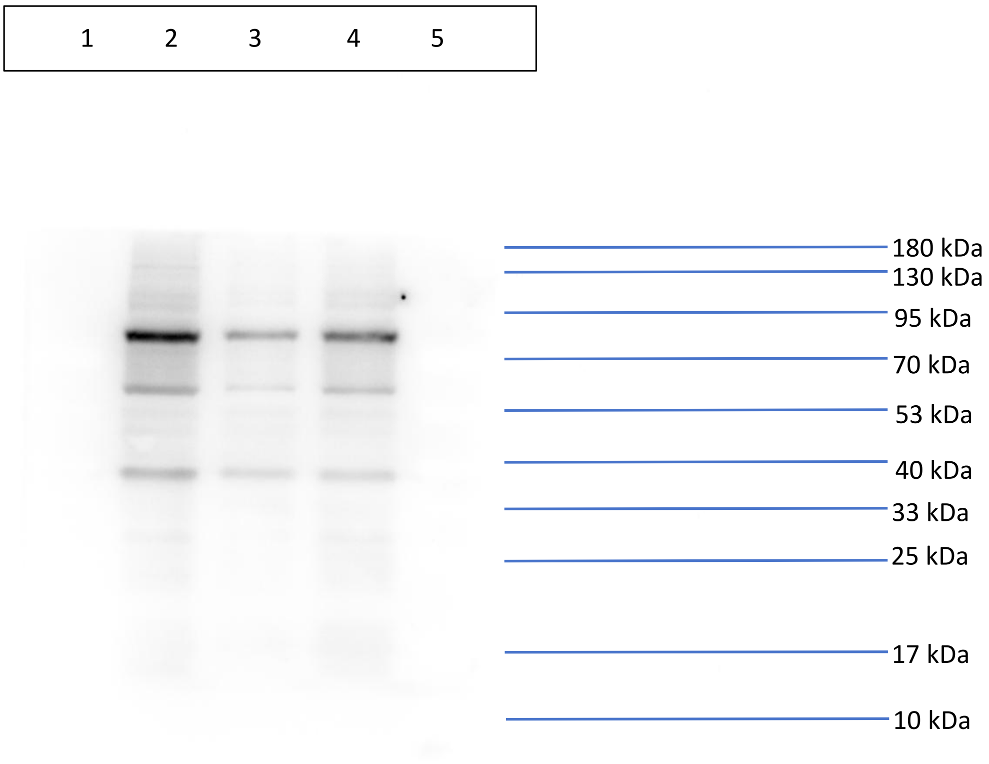

Lane1：Protein marker

Lane2：si-NC

Lane3：si-KIF18A-1

Lane4：si-KIF18A-1+oe5-LOX

Lane5：Protein marker

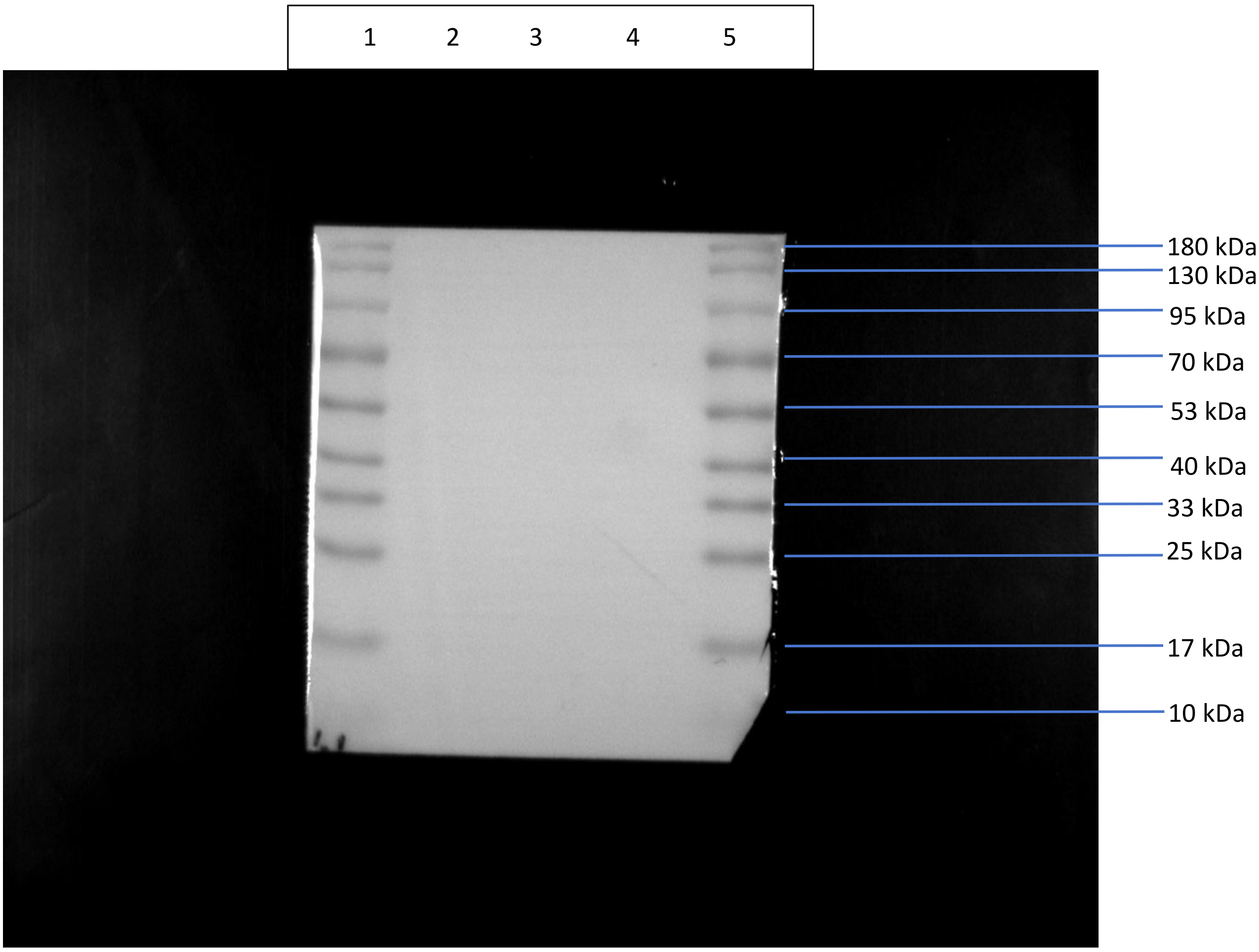

Lane1：Protein marker

Lane2：si-NC

Lane3：si-KIF18A-1

Lane4：si-KIF18A-1+oe5-LOX

Lane5：Protein marker

GAPDH

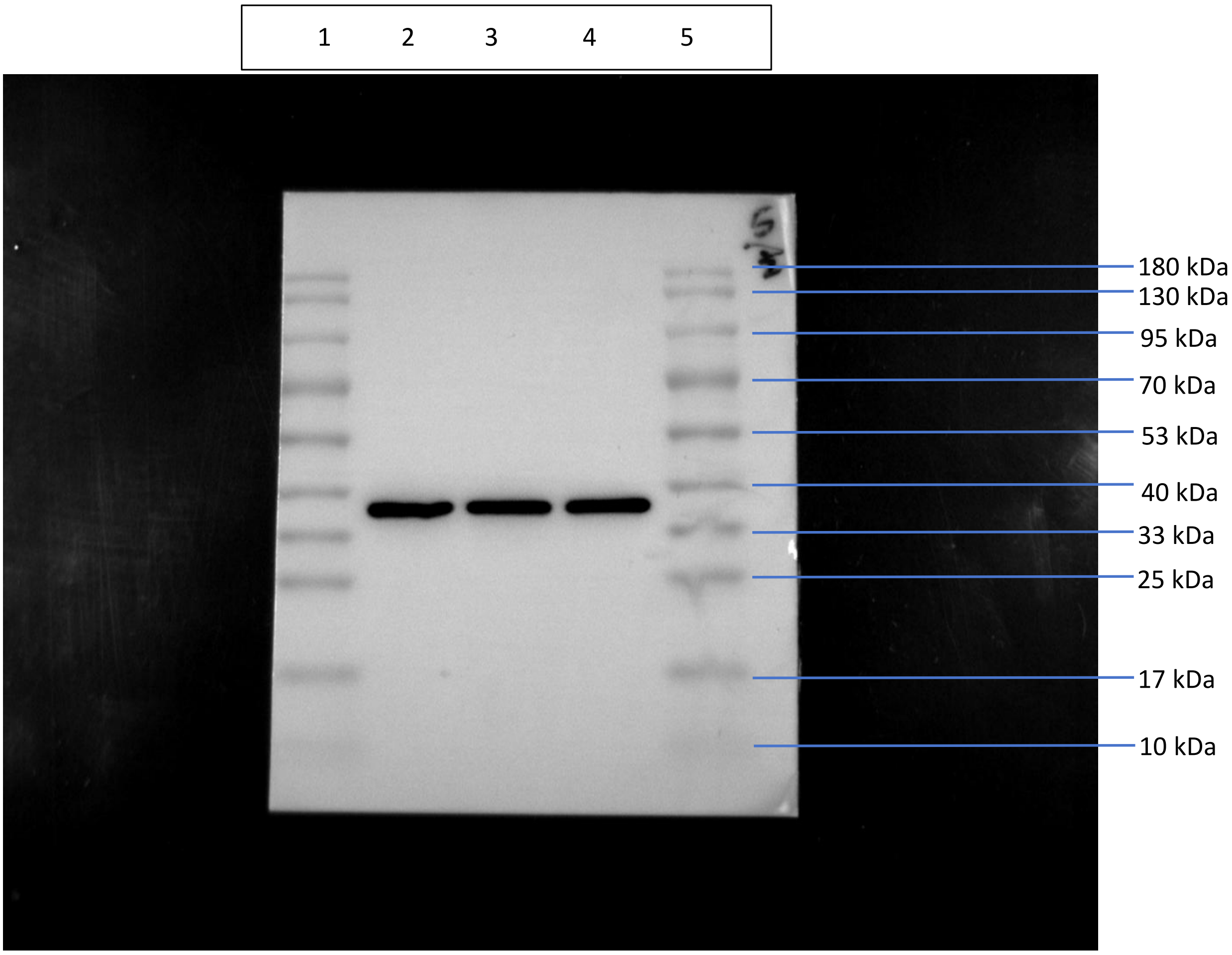

Lane1：Protein marker

Lane2：si-NC

Lane3：si-KIF18A-1

Lane4：si-KIF18A-1+oe5-LOX

Lane5：Protein marker

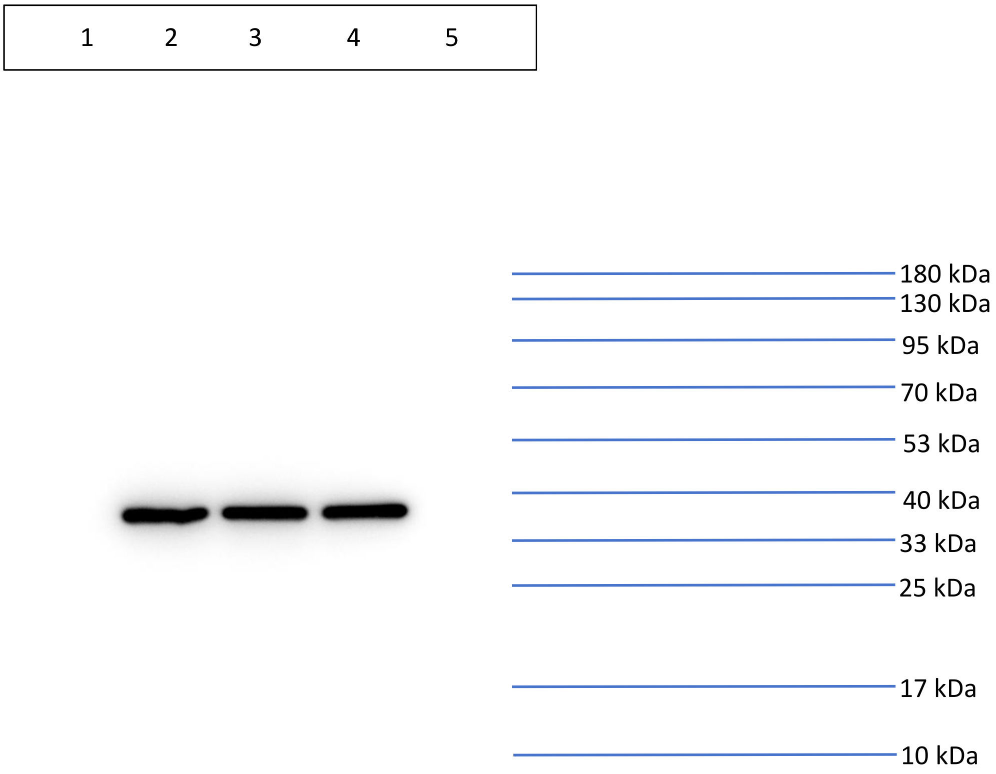

Lane1：Protein marker

Lane2：si-NC

Lane3：si-KIF18A-1

Lane4：si-KIF18A-1+oe5-LOX

Lane5：Protein marker

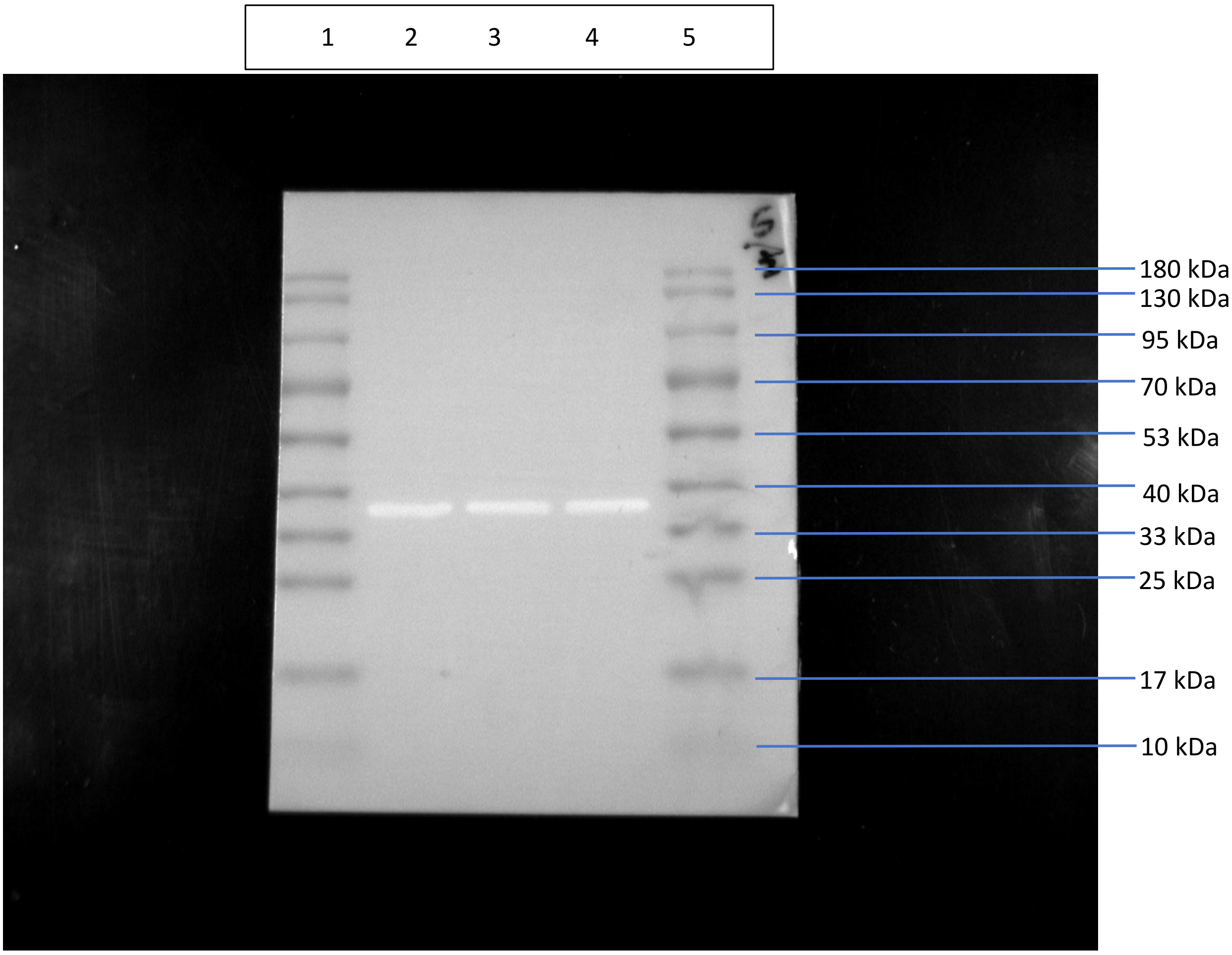

Lane1：Protein marker

Lane2：si-NC

Lane3：si-KIF18A-1

Lane4：si-KIF18A-1+oe5-LOX

Lane5：Protein marker
